# The role of borosilicate glass in Miller–Urey experiment

**DOI:** 10.1038/s41598-021-00235-4

**Published:** 2021-10-25

**Authors:** Joaquín Criado-Reyes, Bruno M. Bizzarri, Juan Manuel García-Ruiz, Raffaele Saladino, Ernesto Di Mauro

**Affiliations:** 1 Laboratorio de Estudios Cristalográficos, Instituto Andaluz de Ciencias de la Tierra, Consejo Superior de Investigaciones Científicas, Universidad de Granada, Avenida de las Palmeras 4, Armilla, 18100 Granada, Spain; 2 Ecological and Biological Sciences Department (DEB), University of Tuscia, Via S. Camillo de Lellis snc, 01100 Viterbo, Italy

**Keywords:** Biogeochemistry, Chemistry

## Abstract

We have designed a set of experiments to test the role of borosilicate reactor on the yielding of the Miller–Urey type of experiment. Two experiments were performed in borosilicate flasks, two in a Teflon flask and the third couple in a Teflon flask with pieces of borosilicate submerged in the water. The experiments were performed in CH_4_, N_2_, and NH_3_ atmosphere either buffered at pH 8.7 with NH_4_Cl or unbuffered solutions at pH ca. 11, at room temperature. The Gas Chromatography-Mass Spectroscopy results show important differences in the yields, the number of products, and molecular weight. In particular, a dipeptide, multi-carbon dicarboxylic acids, PAHs, and a complete panel of biological nucleobases form more efficiently or exclusively in the borosilicate vessel. Our results offer a better explanation of the famous Miller's experiment showing the efficiency of borosilicate in a triphasic system including water and the reduced Miller–Urey atmosphere.

## Introduction

The 1953’s publication of the Miller–Urey experiment opened the door to the scientific investigation of the origin of life^1^. In this brilliant experiment, Miller and Urey demonstrated that electrical sparking a mixture of methane, ammonia, and hydrogen in the presence of water produces amino acids within a variety of organic compounds. The impact of these results was so high that its mind-opening relevance hardly fades over time^2^. Different gas mixtures have been explored^3–7^, and the yielding and molecular diversity were confirmed with modern analytical techniques^8^, including original sample remnants of early Miller experiments^9,10^. Variations of the original Miller apparatus have been used, but the experiments were always performed within borosilicate flasks. Interestingly, the initial pH of most of the canonical mixtures aiming to mimic the early Earth atmosphere in Miller–Urey experiments is highly alkaline. As reported by Miller^1,3^, under these alkaline conditions, silica dissolves: the higher the pH and temperature, the higher the solubility of silica (Fig. S1). Therefore, it could be expected that upon contact of the alkaline water with the inner wall of the borosilicate flask, even this reinforced glass will slightly dissolve releasing silica and traces of other metal oxides, offering silanol groups to the gas phase and the liquid water and vapor. Motivated by the biomimetic role of silica in mineral self-organized structures, such as silica-carbonate biomorphs^11–13^ and its catalytic role in prebiotic chemistry^14,15^, we designed a set of experiments to test the possible influence of silica on the classical Miller experiments.

## Results

**Figure 1:**
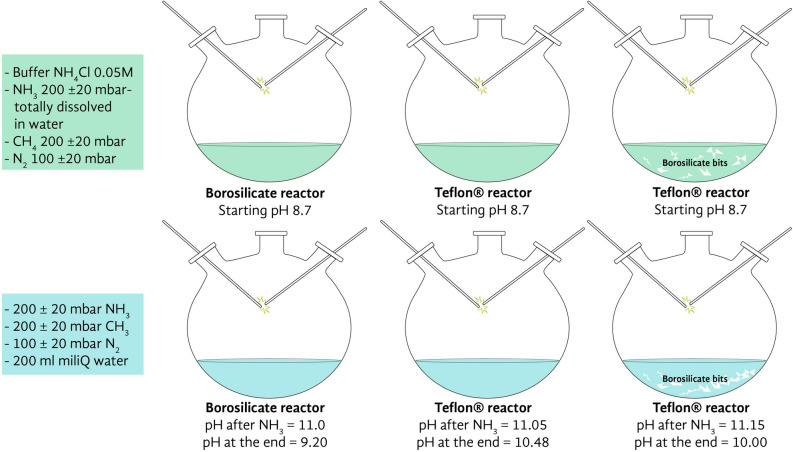
The experimental design. Six electric discharge experiments were performed in two different flasks, one made of borosilicate glass, the other of Teflon. Three experiments were performed to test the effect of the borosilicate glass. One in the borosilicate reactor, one in the Teflon reactor, a third one in the Teflon reactor containing pieces of borosilicate glass. The three experiments were repeated with NH_4_Cl buffer at pH 8.7 and without buffer at pH ca. 11.

Figure 1 shows the experimental concept. Three types of experiments were carried out under two different chemical conditions, one unbuffered with a starting pH value of ca. 11, the other buffered at pH 8.7. One of the experiments was performed in a borosilicate reactor (hereafter BSR unbuffered and BSRB buffered) as used in Miller-type experiments. A second was performed in a Teflon reactor (TFR unbuffered and TFRB buffered), a third in a Teflon reactor with centimeter pieces of borosilicate glass submerged in the water (TFBSR unbuffered and TFBSR/B buffered). After proceeding with the electrical discharges, the differences in color of the collected samples were visually evident (Fig. S2). In what follows, we describe the results of these experiments.

**Figure 2:**
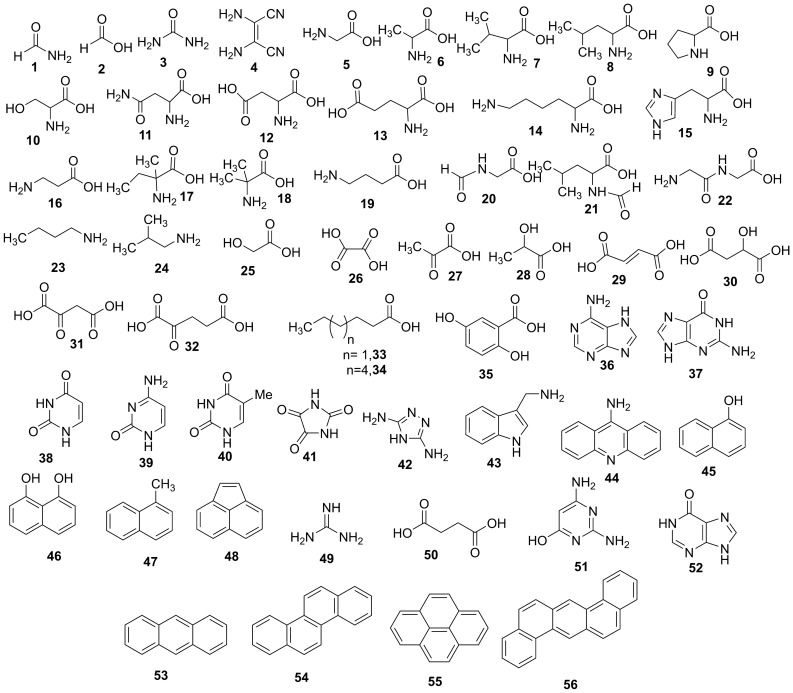
Overall view of the organic compounds produced during the six electric discharge experiments performed in borosilicate glass and Teflon flasks. Formamide **1**, formic acid **2**, urea **3**, diaminomaleonitrile **4**, glycine **5**, alanine **6**, valine **7**, leucine **8**, proline **9**, serine **10**, asparagine **11**, aspartic acid **12**, glutamic acid **13**, lysine **14**, histidine **15**, β-alanine **16**, iso-valine **17**, α-amino isobutyric **18**, γ-aminoisobutyric acid **19**, *N*-formyl glycine **20**, *N*-formyl leucine **21**, glycylglycine **22**, *n*-butanamine **23**, 2-methylpropanamine **24**, glycolic acid **25**, oxalic acid **26**, pyruvic acid **27**, lactic acid **28**, fumaric acid **29**, malic acid **30**, oxaloacetic acid **31**, α-ketoglutaric acid **32**, *n*-hexanoic acid **33**, *n*-nonanoic acid **34**, gentisic acid **35**, adenine **36**, guanine **37**, uracil **38**, cytosine **39**, thymine **40**, parabanic acid **41**, 3,5-diamino-1,2,4-triazole **42**, 1(H)-indole-3-methanamine **43**, 9-acridinamine **44**, 1-hydroxynaphtalene (naphtol) **45**, 1,8-dihydroxynaphtalene **46**, methylnaphthalene **47**, acenaphthylene **48**, guanidine **49**, succinic acid **50**, 2,4-diamino-6-hydroxypyrimidine **51**, hypoxanthine **52**, anthracene **53,** crysene **54**, pyrene **55**, and dibenz(*a,h*)anthracene **56**.

We used a single flask Miller-Urey apparatus where electrodes, water, and the components of the atmosphere were joined in one single reaction flask made either of borosilicate or Teflon. The borosilicate flask (Duran) had a volume of 3 L, the Teflon flask of 1.5 L (Fig. S3). A Tesla coil provided the 30 kV to ignite the electric arc between the tungsten electrodes. The flasks were filled with water to a volume of 200 mL, so the sparking took place in the gas phase (Figure S4 and S5). All the experiments were performed at room temperature, with a water vapor pressure of ca. 24 mbars, to remove thermal effects for a more effective comparison (see further details in SI# 1). We selected one of the most effective Miller atmospheres made of ammonia (200 ± 20 mbar), methane (200 ± 20 mbar), and nitrogen (100 ± 20 mbar). Ammonia and nitrogen are considered ubiquitous components of the early atmosphere^16,17^. The initial pH value of the water was ca. 11.1, and it decreased during the run by almost two units in the borosilicate flask and one unit in the Teflon reactor. The experiments with the NH_4_Cl buffer were aimed to keep the pH constant in the region where the speciation is not only H_4_SiO_4_^=^ silicic acid but also has a small contribution of H_3_SiO_4_^-^. They also mimic the presumptive presence of the ammonium ion NH_4_^+^ in the primitive ocean^18^ and optimize the synthesis of amino acids by the Strecker condensation^19^. The crude was analyzed by gas chromatography associated to mass spectrometry (GC–MS) after derivatization of the sample to corresponding trimethylsilyl ethers (TMS), the yield of reaction products was reported as both micrograms of product per 1.0 mg of crude and mg of product per total amount of crude (SI#1). The structure of reaction products was tentatively assigned by comparison of the mass fragmentation spectra with the original one deposited in the database and further confirmed, when the similarity index was lower than 98%, by the co-injection method with original standards (SI#3). The most abundant reaction products are described in Fig. 2 and Table S2 (buffered condition) and Table S3 (unbuffered condition), the mass to charge (*m/z*) ratio values and relative peak abundances of products are in SI#2 (Table S4), while GC chromatograms and original *m/z* fragmentation spectra are in SI#3 and SI#4, respectively. As shown in Fig. 2 and Tables S2-S3, a large panel of elemental prebiotic chemical precursors (ECP) **1**–**4**, amino acids and alkyl amines **5**–**24**, carboxylic acids **25**–**35**, RNA and DNA nucleobases **36**–**40**, and aromatic and heteroaromatic derivatives **41**–**48** were tentatively assigned in different yield and selectivity depending on the specific experimental conditions. The total yield of compounds **1**–**48** grouped per chemical class similarity is reported in Table 1. In spite of these circumstantial indications, in our opinion the possibility exists that there could still be in principle an effect exerted by the size and shape of the reactor, even though we consider unlikely that these could significantly modify the selectivity and efficiency of the observed reaction pathways. The same applies for the electrode gap variation. The correlation between product distribution and variation of electrode geometry has been discussed^7^.

**Table 1:** Total yield of products grouped for chemical class: ECP elemental prebiotic chemical precursors compounds, amino acids, carboxylic acids, nucleobases, aromatic miscellanea, amines.

Entry	Class	BSRB	TFRB	TFBSR/B*	BSR	TFR	TFBSR*
		Yield (µg product/1.0 mg of crude)					
1	ECP	122.96	31.29	100.91	129.64	35.46	90.18
2	Amino acids	159.45	27.1	60.53	111.19	51.20	87.41
3	Carboxylic acids	36.65	11.91	26.49	46.3	28.21	80.89
4	Nucleobases	14.01	7.34	5.83	16.3	4.69	14.02
5	Aromatic miscellanea	26.95	7.14	10.86	23.07	48.25	33.58
6	Amines	33.80	33.50	34.79	77.07	78.19	69.06
7	Total amount	393.82	118.28	239.41	403.57	246	375.14

*BSRB* borosilicate in buffer, *TFRB* Teflon in buffer, *TFBSR/B* Teflon in buffer in the presence of pieces of borosilicate, *BSR* borosilicate without buffer, *TFR* Teflon without buffer, *TFBSR* Teflon without buffer in the presence of pieces of borosilicate, *ECP* elemental prebiotic chemical precursors.

**Figure 3:**
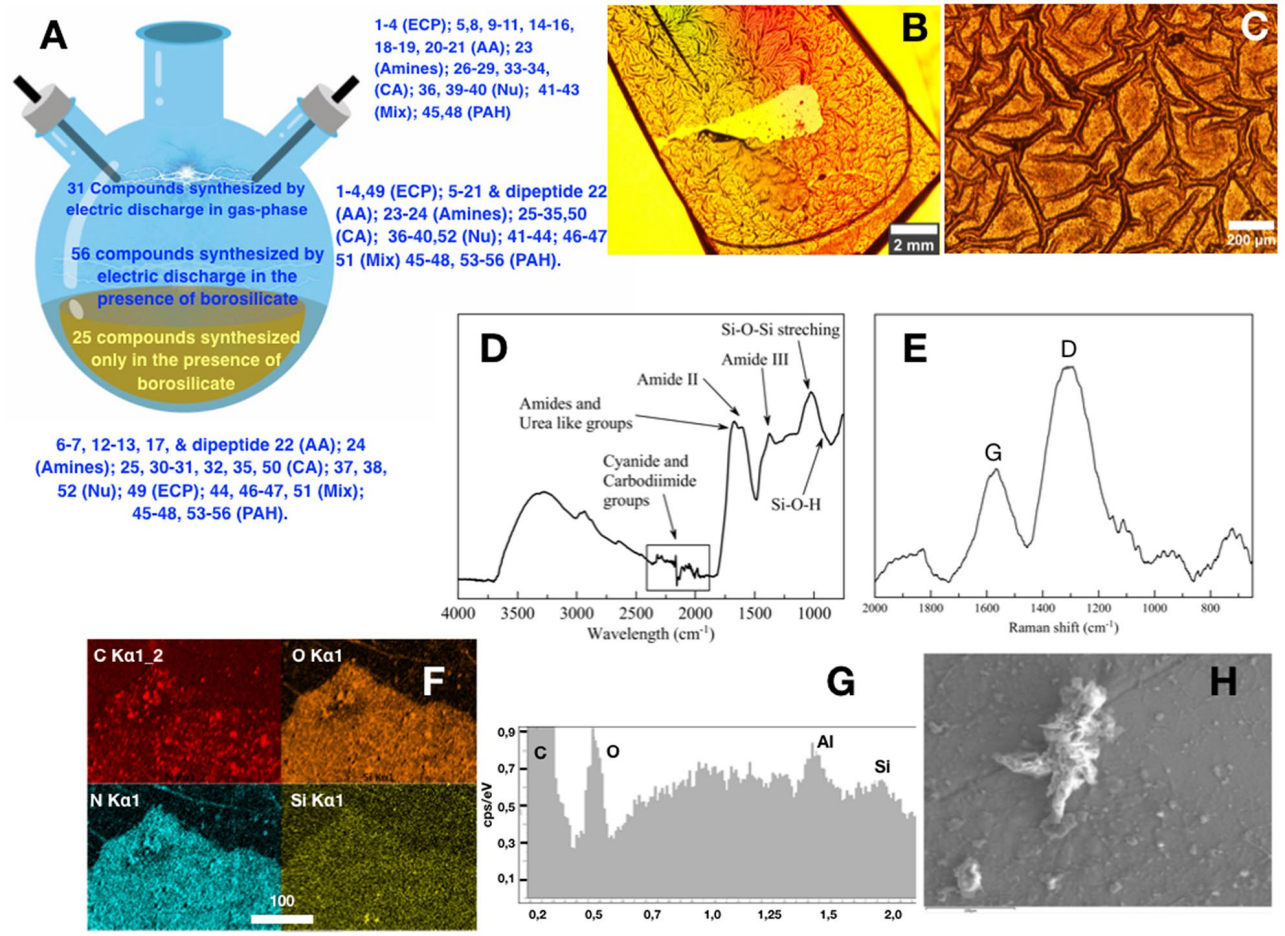
Reaction products obtained in the experiments. **(A)** Comparison of molecular diversity among the three experiments. Note that amino acids, carboxylic acids, and nucleobases were always produced in the presence of borosilicate in total percentage higher than other products (C-1 chemical precursors and amines), thus favoring the mass balance towards the formation of compounds that are, in principle, useful intermediates for molecular evolution. **(B,C)** Optical micrographs of the wet **(B)** and dry **(C)** organic film covering the inner wall of the borosilicate flask; **(D)** infrared spectra of the organic skin; **(E)** Raman spectra of the organic skin after carbonation, showing D and G peaks. **(F)** EDX mapping of the organic film; **(G)** EDX elemental composition of the particle shown in **(H)** showing the existence of silicon in the film.

Overall, these results confirm the visual assessment that the brown broth obtained in the borosilicate experiments contained much more organic compounds than those of the Teflon experiments, irrespective of the buffering (Fig. S2; Table 1, entry 7). A larger panel of reaction products was obtained in borosilicate with respect to Teflon alone (48 compounds versus 31; Tables S2-S3), and several amino acids, a dipeptide, carboxylic acids and aromatic miscellanea (for a total of 17 compounds) were produced only in the presence of borosilicate (Tables S2-S3) (Fig. 3A).

Borosilicate increased the yield of ECP **1–4** relative to Teflon alone (Table 1, entry 1). Ab initio atomistic simulation of the Miller-Urey experiment postulated the barrier-less formation of **1** and **2** from a reducing atmosphere^20^, and traces of these compounds of key importance in prebiotic chemistry^21^ were recently detected by mimicking a meteoritic impact in the pristine atmosphere^22^. DAMN **4** is a common intermediate in the synthesis of nucleobases from HCN and **1**, while **3** is a component of the organic pool in the primitive Earth^23^. A total of 17 amino acids **5**–**21**, a dipeptide **22**, and two simple amines **23**–**24**, were detected in the crude (Fig. 2). The total yield of amino acids was higher in the borosilicate flasks than in Teflon alone (Table 1, entry 2). In addition, amino acids **6**–**7**, **12**–**13**, and **17**, and the dipeptide **22**, formed exclusively in the presence of borosilicate (Fig. 2, Tables S2-S3).

The synthesis of **22,** as well as that of formylated amino acids **20**–**21** (Fig. 2), is of prebiotic relevance and was probably favored by the formation of carbodiimide from **1,** a borosilicate-catalyzed process^24^. Once formed, carbodiimide can activate amino acids towards the formation of the peptide bond with contemporaneous release of urea^25^. Carboxylic acids **25**–**35** (from C-1 to C-9) were also tentatively identified in the reaction mixture (Fig. 2), the highest total yield being obtained in the presence of borosilicate (Table S3, entry 3). Carboxylic acids **25**, **30**–**31**, **32,** and **35** were absent in the experiment performed in Teflon alone (Tables S1-S2). The beneficial role of borosilicate was further confirmed in the synthesis of nucleobases. In this latter case, borosilicate systems afforded the complete set of nucleobases **36**–**40**, while only **36**, **39,** and **40** were detected in the Teflon flask (Tables S2-S3). Again, the total yield of nucleobases was highest in the presence of borosilicate (Table 1, entry 4). A slightly different behavior was observed in the formation of aromatic miscellanea **41**–**48**, including polycyclic aromatic derivatives **45**–**48** (PAHs) (Fig. 2, Tables S2-S3). PAHs are important contributors to the overall pool of organic carbon in the universe and potential candidates in the “aromatic world” hypothesis^26^. Aromatic derivatives prevailed in the borosilicate flask under buffered conditions, but this trend was reversed in the absence of the buffer, in which case the highest total yield was obtained in Teflon alone (Table 1, entry 5). The effect of the buffer in the selectivity of the reactions and possible reaction pathways for the formation of compounds **1**–**48** are discussed in Supplementary text SI#5.

## Discussion

Our results demonstrate that the wall of the reactors plays a crucial role in the synthesis of organic compounds in the Miller-Urey experiment. As summarized in Fig. 3A, the molecular diversity is minimal in the Teflon reactor, increases when submerging pieces of borosilicate glasses in the water of the Teflon reactor, and it reaches a maximum in both molecular variety and yielding in the borosilicate reactor. Furthermore, few hours after sparking, the wall of the borosilicate flask is covered by a thin brown film of organic matter. Noticeably, this film only forms on the part of the wall above the water level of the reactor. The color of the solution in the borosilicate reactors is yellow–brown and is full of brown organic particles visible to the naked eye. In none of the Teflon reactors, the formation of this organic film was observed. However, in the experiment performed with a Teflon reactor “seeded” with pieces of borosilicate glass, brown particles were noticed inside the solution.

The silanol groups on the surface of the glass, and traces of metal that could be released by dissolution under the alkaline conditions of the experiment may contribute to the observed reactivity^27,28^. The presence of Si–O–H groups enhanced by the alkaline conditions facilitates the absorption of the organic molecules synthesized in the gas and the liquid water in contact with the glass^29^. This could explain the formation of the brown film covering the inner surface of the borosilicate flask. The film appears as a translucent orange matrix under the optical microscope (Fig. 3B,C). The infrared and Raman spectra of the freshly formed film (Fig. 3D,E) show the characteristic absorption bands for HCN oligomers^30^. GC–MS confirms that the film is mainly made of HCN oligomers, in accordance with previously reported data. It also shows that it works as a matrix embedding and concentrating organic molecules, including urea **3**, glycine **5**, lactic acid **28**, adenine **36**, cytosine **39**, guanidine **49**, succinic acid **50**, 2,4-diamino-6-hydroxypyrimidine **51**, hypoxanthine **52**, and four polycyclic aromatic hydrocarbons, namely anthracene **53,** chrysene **54**, pyrene **55**, and dibenz(*a,h*) anthracene **56** (Fig. 2, Table S5). Among them, **49**–**56** were not previously detected in the liquid fraction of the experiment. As a general trend, the total yield of these latter compounds was found to increase after acid hydrolysis^31^, highlighting the possibility that the treatment favored their extraction from the solid matrix (See supplementary information Table S5 condition A vs. condition B). The EDX analysis of the film reveals the existence of a significant amount of silica (Fig. 3F,H and Figure S8). The formation of organosilicon compounds is most likely responsible for the incomplete mass balance relative to the crude (Table 1). In addition, the highest total yield for the reaction products observed under unbuffered conditions is in accordance with a possible role of borosilicate as a catalyst for prebiotic processes (Table 1, entry 7).

From the initial bet of Bernal and Goldsmichdt for montmorillonite^32^, many other minerals have been proposed to speed up the synthesis of specific molecules required for life as we know it, namely other clays, zeolites, sulfides, iron oxide, layered hydroxides, silica, etc.^15,33,34^. Experimental and theoretical work has been published to support these claims^35^. In particular, simple variations in environmental mineral composition lead to differentiation of distinct chemical pathways^36^, encompassing the role of mineral surface in the prebiotic origin of amino acids^37^ and peptides^38^, mechanochemical solid-state transformations^39^, and borosilicate-mediated formose condensation in the synthesis and stabilization of biologically relevant four and six-carbon sugars^38^. However, we still miss a good understanding of the structural reasons why and how mineral surfaces catalyze reactions relevant to prebiotic chemistry and the origin of life^40^. The importance of our results lies in the fact that, for the first time, the role of borosilicate has been experimentally demonstrated in a type of synthesis of the utmost relevance for the inorganic generation of organic compounds from scratch. The famous Miller-Urey synthesis triggered by sparking would be highly efficient at any place of the universe, provided a mineral surface is available. Noteworthy, silica and silicates also trigger the formation of insoluble organic matrices that serve as niches for the preservation and concentration of forming prebiotic molecules. These abiotic organic films may have formed in Earth-like planets and moons as Mars and several moons of the solar system^41–43^. For instance, a large fraction of the organic matter found in Archean rocks and to be found in the robotic exploration of Mars might reasonably be of inorganic origin. The putative role of the organic film triggered by the borosilicate reactors as a milieu for absorption and concentration of organic molecules should be further investigated. And indeed, the formation and properties of these organic films must be explored with different mineral surfaces and different atmospheres.

## Conclusion

The experiment is especially important in the framework of the new ideas about the Hadean Earth in which the concomitance of a reduced atmosphere, electrical storms, silicate-rich rocky surfaces, and liquid water is expected^31,44^. Our results demonstrate that silica and silicates drastically enhance Miller’s prebiotic synthetic routes affording important differences in the yields, in the number of products, and in their increased chemical information described by the number of carbon and nitrogen atoms composing the molecules, which are obtained starting from 1-carbon atom and 1-nitrogen atom precursors. Irrespective of the possible lack of correspondence^45,46^ of the early Earth atmosphere with that originally proposed by Miller-Urey, these results show the efficiency and the prebiotic worth of the borosilicate/spark discharge system. The presence of high molecular weight products is exemplified by the presence of a dipeptide, of multi-carbon atoms dicarboxylic acids, of PAHs^47^, of a complete panel of biological nucleobases, and, markedly, by the rich variety of different classes of compounds. In summary, Miller recreated in his experiments the atmosphere and waters of the primitive Earth. The role of the rocks was hidden in the walls of the reactors.

## Methods

The electric discharge was performed under unbuffered and buffered solution (NH_4_Cl, 0.05 M, pH 8.7) in a Teflon apparatus and compared with a classical borosilicate reactor as a reference (these experiments will be indicated in the follow as TFR Teflon reactor unbufered, TFRB Teflon buffered, BSR borosilicate reactor unbuffered and BSRB borosiclicate reactor buffered) (detailed experimental set-up is in SI#1). Two more experimental conditions were studied: (i) the electric discharge in the absence of the buffer; and (ii) the electric discharge in the Teflon apparatus in the presence of borosilicate bits (17 g), under both buffered (TFBSR/B) and unbuffered (TFBSR) conditions. After the work-up, the reaction was lyophilized and immediately analyzed by GC–MS. The samples stored at −80 °C for one or two weeks (to replicate analysis) showed the same composition of freshly analyzed counterparts. This control was performed in order to rule out possible ageing-related variations of the reaction products at −80 °C, a possibility that was previously highlighted^48^. In detail, in a round bottom flask *N*,*N*-bis-trimethylsilyl trifluoroacetamide (420 µL; Merck > 99%) and a solution of pyridine (200 µL; Merck > 99%) were added to 10 mg of crude of the reaction. The mixture was left under magnetic stirring at 90 °C for 4 h. Thereafter the solution was cooled down to 25 °C and 2.0 µL of the solution were used for the GC–MS analysis. Chromatographic conditions: CP8944 column (WCOT fused silica, film thickness 0.25 μm, stationary phase VF-5 ms, Øί 0.25 mm, length 30 m), injection temperature 280 °C, detector temperature 280 °C, gradient 100 °C × 2 min, then 10 °C/min for 60 min. GC–MS fragmentation spectra were recovered by using a triple quadrupole MS analyzer as full scan and single ion research modes, and compared with commercially available electron mass spectrum libraries. The libraries we used (NIST 2020 libraries; NIST/EPA/NIH Mass Spectral Library: c3oh_ci, c4h10_ci, ch4_all, ch4_drug, ch4_fda, libr_gp, libr_tr, libr_tx) are settled to contain more than 1.3 million spectra including most of the compounds of biological relevance and known products deriving from the chemistry of HCN and formamide^49^. These libraries also include isomeric structures. They tentatively identify unknown structure on the basis of the crossing of multiple experimental parameter values (i.e., retention time, m/z distribution and intensity of the corresponding fragmentation peaks)^50^. All products have been recognized with a similarity index (S.I.) greater than 98%. In the case of valine (**7)** isovaline (**17),** α-NH_2_-isobutyric acid (**18**), γ-NH_2_-butiric acid (**19**), butanamine (**23)** and isobutylamine (**24)**, for which the similarity index was encompassed between 97 and 98%, the qualitative assignment was performed by co-injection method, repeating the GC–MS analysis after the addition of 0.1 µmol of appropriate standard compounds before the derivatization procedure (original co-injected chromatograms are in SI#3). The yield of reaction products was calculated in triplicate as micrograms of product per 1.0 mg of the crude and mg of product per total amount of the reaction crude, using the calibration line procedure, or in alternative (for compounds **17**–**19** and **23**–**24**) the internal standard method in the presence of betulinic acid (3β-hydroxy-20(29)-lupaene-oic acid) as internal standard (0.2 mg, 0.00045 mmol) (the general description of calibration line procedure and internal standard method is in SI#7).

## Supplementary Information

Supplementary Information.

## Data Availability

All data is available in the main text or the supplementary materials.

## References

[1] Miller SL (1953). A production of amino acids under possible primitive earth conditions. Science.

[2] Ball P (2015). Elegant Solutions: Ten Beautiful Experiments in Chemistry.

[3] Miller SL (1955). Production of some organic compounds under possible primitive earth conditions1. J. Am. Chem. Soc..

[4] Orò J (1963). Synthesis of organic compounds by electric discharges. Nature.

[5] Schlesinger GS, Miller SL (1983). Prebiotic synthesis in atmospheres containing CH_4_, CO, and CO_2_. J. Mol. Evol..

[6] Miyakawa S, Yamanashi H, Kobayashi K, Cleaves HJ, Miller SL (2002). Prebiotic synthesis from CO atmospheres: Implications for the origins of life. PNAS.

[7] Cooper GJT, Surman AJ, McIver J, Colón-Santos SM, Gromski PS, Buchwald S, Suárez Marina I, Cronin L (2017). Miller-Urey spark-discharge experiments in the deuterium world. Angew. Chem. Int. Ed..

[8] Wollrab E, Scherer S, Aubriet F, Carré V, Carlomagno T, Codutti L, Ott A (2016). Chemical analysis of a “Miller-type” complex prebiotic broth: Part I: Chemical diversity, oxygen and nitrogen based polymers. Orig. Life Evol. Biosph..

[9] Johnson AP, Cleaves HJ, Dworkin JP, Glavin DP, Lazcano A, Bada JL (2008). The Miller volcanic spark discharge experiment. Science.

[10] Parker ET, Cleaves HJ, Dworkin JP, Glavin DP, Callahan M, Aubrey A, Lazcano A, Bada JL (2011). Primordial synthesis of amines and amino acids in a 1958 Miller H2S-rich spark discharge experiment. PNAS.

[11] Garcia-Ruiz JM (2003). Self-assembled silica-carbonate structures and detection of ancient microfossils. Science.

[12] Garcia-Ruiz JM, Melero-Garcia E, Hyde ST (2009). Morphogenesis of self-assembled nanocrystalline materials of barium carbonate and silica. Science.

[13] Montalti M, Zhang G, Genovese D, Morales J, Kellermeier M, García-Ruiz JM (2017). Local pH oscillations witness autocatalytic self-organization of biomorphic nanostructures. Nat. Commun..

[14] Saladino R, Carota E, Botta G, Kapralov M, Timoshenko GN, Rozanov AY, Krasavin E, Di Mauro E (2015). Meteorite-catalyzed syntheses of nucleosides and of other prebiotic compounds from formamide under proton irradiation. PNAS.

[15] Saladino R, Di Mauro E, García-Ruiz JM (2019). A universal geochemical scenario for formamide condensation and prebiotic chemistry. Chem. Eur. J..

[16] Brandes JA, Boctor NZ, Cody GD, Cooper BA, Hazen RM, Yoder HS (1998). Abiotic nitrogen reduction on the early Earth. Nature.

[17] Zahnle K, Schaefer L, Fegley B (2010). Earth’s earliest atmospheres. Cold Spring Harbor Perspect. Biol..

[18] Bada JL, Miller SL (1968). Ammonium ion concentration in the primitive ocean. Science.

[19] Miller, S. L. & Van Trump, J. E. *Origin of Life. *135–141 (Springer, 1981).

[20] Saitta AM, Saija F (2014). Miller experiments in atomistic computer simulations. PNAS.

[21] Saladino R, Botta G, Pino S, Costanzo G, Di Mauro E (2012). Genetics first or metabolism first? The formamide clue. Chem. Soc. Rev..

[22] Ferus M, Pietrucci F, Saitta AM, Knížek A, Kubelík P, Ivanek O, Shestivska V, Civiš S (2017). Formation of nucleobases in a Miller–Urey reducing atmosphere. PNAS.

[23] Ferus M, Nesvornỳ D, Šponer J, Kubelík P, Michalčíková R, Shestivská V, Šponer JE, Civiš S (2015). High-energy chemistry of formamide: A unified mechanism of nucleobase formation. PNAS.

[24] Saladino R, Barontini M, Cossetti C, Di Mauro E, Crestini C (2011). The effects of borate minerals on the synthesis of nucleic acid bases, amino acids and biogenic carboxylic acids from formamide. Orig. Life Evol. Biosph..

[25] Costanzo G, Saladino R, Crestini C, Ciciriello F, Di Mauro E (2007). Formamide as the main building block in the origin of nucleic acids. BMC Evol. Biol..

[26] Ehrenfreund P, Rasmussen S, Cleaves J, Chen L (2006). Experimentally tracing the key steps in the origin of life: The aromatic world. Astrobiology.

[27] Iler RK (1979). The Chemistry of Silica.

[28] Rimola A, Costa D, Sodupe M, Lambert JF, Ugliengo P (2013). Silica surface features and their role in the adsorption of biomolecules: Computational modeling and experiments. Chem. Rev..

[29] Parida SK, Dash S, Patel S, Mishra BK (2006). Adsorption of organic molecules on silica surface. Adv. Colloid Interface Sci..

[30] Ruiz-Bermejo M, De la Fuente JL, Rogero C, Menor-Salván C, Osuna-Esteban S, Martín-Gago JA (2012). New insights into the characterization of ‘insoluble black HCN polymers’. Chem. Biodivers..

[31] Zahnle K, Arndt N, Cockell C, Halliday A, Nisbet E, Selsis F, Sleep NH (2007). Emergence of a habitable planet. Space Sci. Rev..

[32] Ferris JP (2006). Montmorillonite-catalysed formation of RNA oligomers: the possible role of catalysis in the origins of life. Philos. Trans. R. Soc. B.

[33] Cairns-Smith A (1987). Genetic Takeover and the Mineral Origins of Life.

[34] Ferris JP (1999). Prebiotic synthesis on minerals: Bridging the prebiotic and RNA worlds. Biol. Bull..

[35] Lambert JB, Senthil AG-T, Kuangbiao M (2010). The silicate-mediated formose reaction: Bottom-up synthesis of sugar silicates. Science.

[36] Surman AJ (2019). Environmental control programs the emergence of distinct functional ensembles from unconstrained chemical reactions. PNAS.

[37] Vinogradoff V (2020). Impact of phyllosilicates on amino acid formation under asteroidal conditions. ACS Earth Space Chem..

[38] Erastova V (2017). Mineral surface chemistry control for origin of prebiotic peptides. Nat. Commun..

[39] Haas M (2020). Mineral-mediated carbohydrate synthesis by mechanical forces in a primordial geochemical setting. Commun. Chem..

[40] Hazen RM, Sverjensky DA (2010). Mineral surfaces, geochemical complexities, and the origins of life. Cold Spring Harb. Perspect. Biol..

[41] Franz HB (2020). Indigenous and exogenous organics and surface–atmosphere cycling inferred from carbon and oxygen isotopes at Gale crater. Nat. Astron..

[42] Vago JL (2017). Habitability on early Mars and the search for biosignatures with the ExoMars Rover. Astrobiology.

[43] Raulin F, Brassé C, Poch O, Coll P (2012). Prebiotic-like chemistry on Titan. Chem. Soc. Rev..

[44] García-Ruiz JM, Van Zuilen MA, Bach W (2020). Mineral self-organization on a lifeless planet. Phys. Life Rev..

[45] Trail D, Watson EB, Tailby ND (2011). The oxidation state of Hadean magmas and implications for early Earth’s atmosphere. Nature.

[46] Cleaves HJ, Chalmers JH, Lazcano A, Miller SL, Bada JL (2008). A reassessment of prebiotic organic synthesis in neutral planetary atmospheres. Orig. Life Evol. Biosph..

[47] Bizzarri BM, Manini P, Lino V, D'Ischia M, Kapralov M, Krasavin E, Mráziková K, Šponer J, Šponer JE, Di Mauro E, Saladino R (2020). High-energy proton-beam-induced polymerization/oxygenation of hydroxynaphthalenes on meteorites and nitrogen transfer from urea: Modeling insoluble organic matter?. Chemistry.

[48] Miyakawa S, Cleaves HJ, Miller SL (2002). The cold origin of life: A implications based on the hydrolytic stabilities of hydrogen cyanide and formamide. Orig. Life Evol. Biosph..

[49] Lai Z, Fiehn O (2018). Mass spectral fragmentation of trimethylsilylated small molecules. Mass Spectrom. Rev..

[50] Buch A, Sternberg R, Szopa C, Freissinet C, Garnier C, Bekri EJ, Rodier C, Navarro-Gonzalez R, Raulin F, Cabane M, Stambouli M, Glavin DP, Mahaffy PR (2009). Development of a gas chromatography compatible sample processing system (SPS) for the in-situ analysis of refractory organic matter in martian soil: Preliminary results. Adv. Space Res..

