# Supplementary material for: The role of borosilicate glass in Miller–Urey experiment
Source: Sci Rep. 2021 Oct 25;11:21009. doi: 10.1038/s41598-021-00235-4 (PMC8545935; doi:10.1038/s41598-021-00235-4)
Supplement: Supplementary file 1 — Supplementary Information. [file 41598_2021_235_MOESM1_ESM.pdf]

THE ROLE OF BOROSILICATE GLASS IN MILLER-UREY EXPERIMENT

Joaquín Criado[1], Bruno M. Bizzarri[2], Juan Manuel García-Ruiz[1]*, Raffaele Saladino[2]*, Ernesto di Mauro[2]

[1]Laboratorio de Estudios Cristalográficos, Instituto Andaluz de Ciencias de la Tierra, Consejo Superior de Investigaciones Científicas–Universidad de Granada, Avenida de las Palmeras 4, Armilla, Granada 18100, Spain.

[2]Ecological and Biological Sciences Department (DEB), University of Tuscia, Via S. Camillo de Lellis snc, 01100, Viterbo (Italy).

*Correspondence to juanmanuel.garcia@csic.es and saladino@unitus.it

SUPPLEMENTARY MATERIALS

Figure S1. Plot on the solubility of silica versus pH

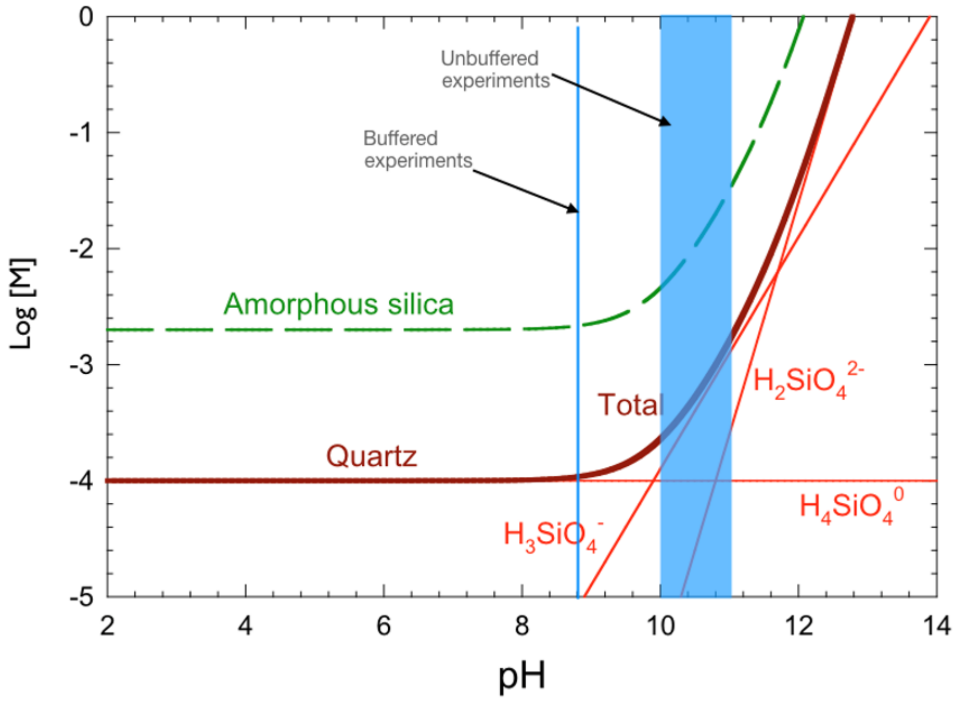

Figure S1. Solubility of silica versus pH. The blue line and the blue band show the pH values of the buffered and unbuffered experiments, respectively.

Figure S2. Samples after the electrical discharge experiments

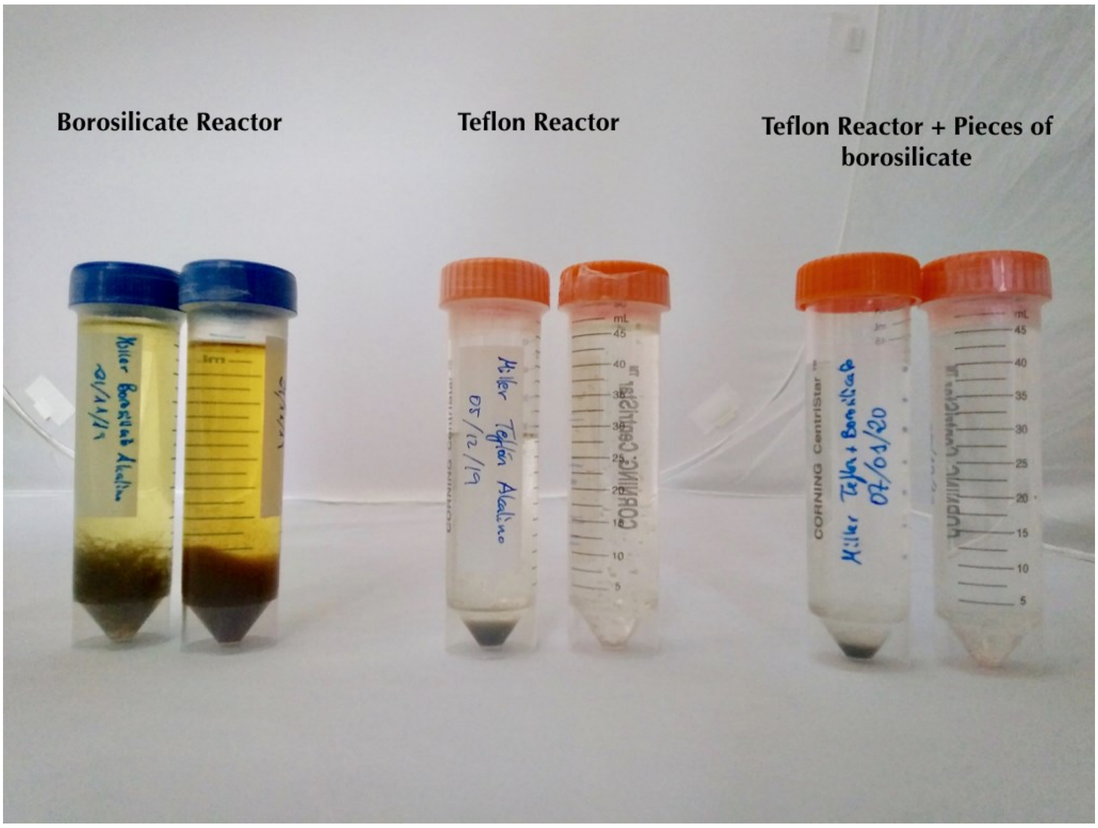

Figure S2. Differences in color of the collected samples after the electrical discharge experiments. The difference in color between the two pairs of solutions (which is more evident in the borosilicate solution) are due to the fact that the one on the left (in each pair of tubes) corresponds to the reaction solution and the other is the washing solution.

SI#1. Technical information

1.1. Experimental set-up

The system is shown in Figures S3-S4. The set-up consists of a reaction flask (blue), which is made either of borosilicate or Teflon. A Tesla coil (purple) is used to provide the necessary voltage for the formation of the electric arc between the tungsten electrodes inside the reaction flask. The injection manifold (black) is connected to the reaction flask via valve number 1. This is the via for introducing the gases into the reaction flask. It also has connected two manometers, one analogue (valve 2) to work with pressures between 40mbar and 1000mbar, and one digital (valve 3), to measure pressures lower than 100mbar. In order to increase the system volume, there is 3-L round flask connected through valve 4. The system of gases (orange) is connected via valve 5 to the injection manifold. Each cylinder of the different gases has its own valve: v7 for ammonia, v8 for methane, and v9 for nitrogen. Finally, the vacuum pump is connected to the injection manifold via valve 6. All the system components are connected using inert rubber tubes.

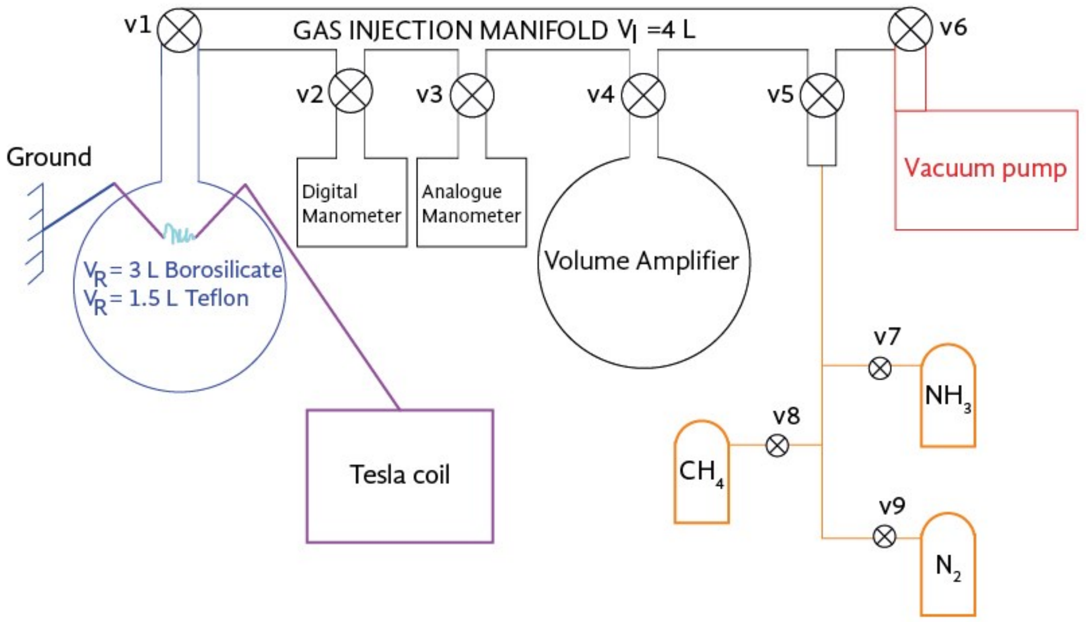

Figure S3. Schetch of the experimental set-up

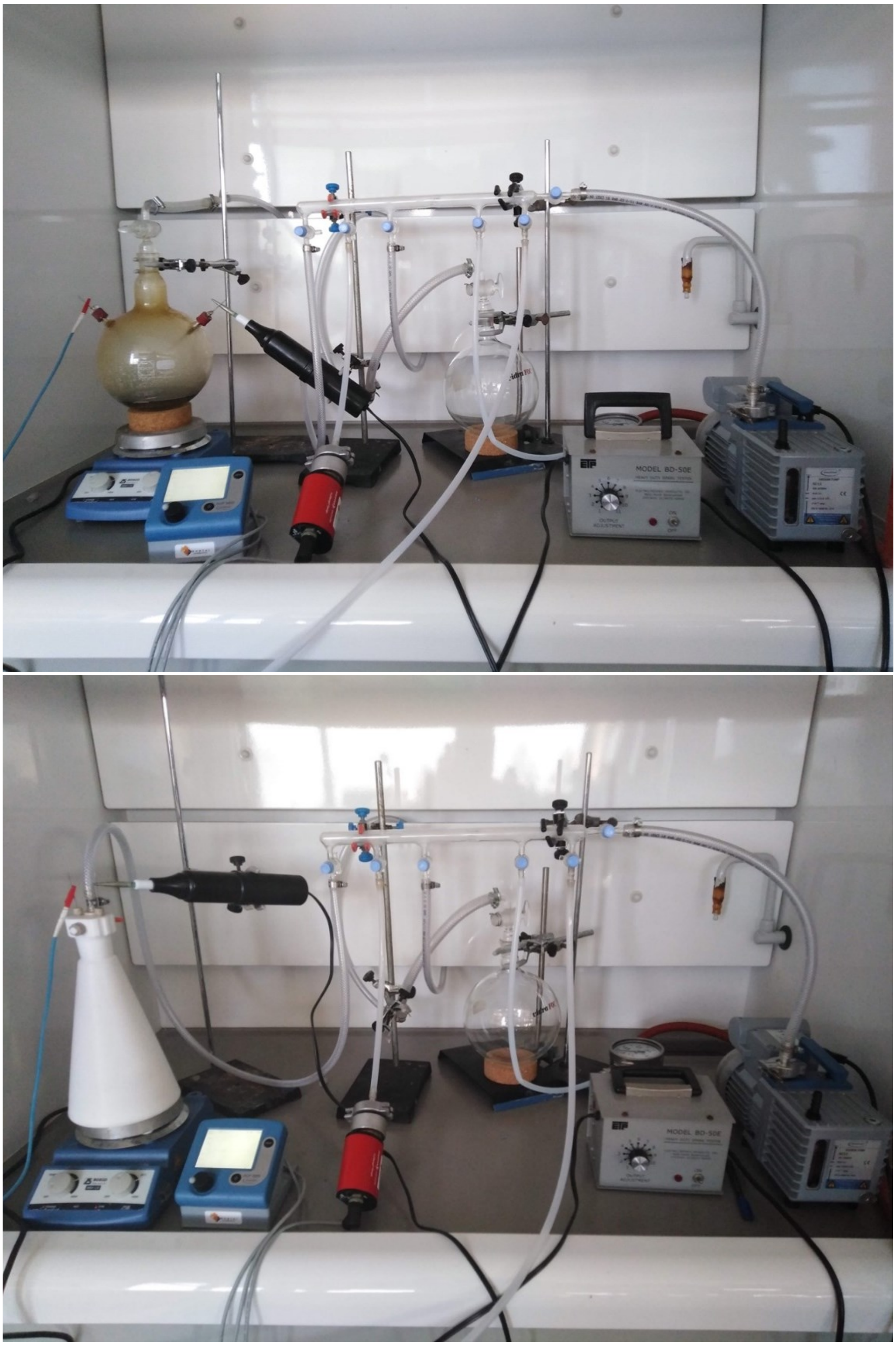

Figure S4. Pictures of the experimental devices showing the borosilicate and the Teflon reactors.

1.2. General equipment cleaning protocol

All the borosilicate and Teflon components have been subjected to 200ºC heat treatment in an oven for 24 hours. They have then been washed with 10 ml of methane, followed by 10 ml of toluene, then with another 10 ml of methane and finally with 30 ml of Milli-Q water, and are allowed to dry.

1.3. Measuring the volume of the injection manifold and procedure for introducing the gas

In order to measure the volume of the injection manifold, we follow the ensuing steps (Figure S4): i) Leave all the valves open except the gas cylinder valves. Then create a vacuum in the system. ii) Once a stable vacuum of less than 1 mbar is attained, close valves v6 and v1 in that order. iii) Introduce 500 mbar of N_2 into the injection chamber (all the valves must be closed except v2, v3, v4 and v5). Close valve v5 after introducing adequate N_2 pressure. iv) Open the injection valve that connects the injection chamber with the reaction flask of known volume (3L borosilicate reaction flask). By measuring the pressure difference, we can calculate the volume of the injection chamber using the following formula:

$P_1 V_1 = P_2 (V_1 + V_R)$

Where P_1 is the initial pressure in the injection chamber (600 mbar), V_1 is the volume of the injection chamber to be solved, P_2 is the pressure in both chambers after opening the injection valve, and V_2 is the total system volume (reaction flask volume + injection chamber volume). In the experiment carried out in the facilities of the Laboratorio de Estudios Cristalográficos (Crystallography Studies Laboratory), we obtained the following pressure and volume values:

P_1= 600 mbar; P_2= 440 mbar; V_1= 4.125 L; V_R=1.5 L (Teflon)

Which means that the volume of the injection manifold is 4.125 L. To prepare the buffer solution, we use 200ml of Milli-Q water, to which we add 0.535 g of NH_4Cl (0.05M). Once we have introduced the solution into the reaction flask, we again create a vacuum in the whole system with all the valves open. The solution will start to bubble, freeing the gases, such as CO_2, O_2 and N_2, dissolved in the solution. We wait for the pressure to stabilize to the vapour pressure of water at the experiment temperature. v) Once the pressure is established, close valve 1, isolating the reaction flask from the injection manifold. vi) Continue to create a vacuum in the gas injection manifold until the pressure is lower than 1 mbar. When this occurs, we will clean the injection manifold, purging twice with the gas that we are going to introduce into the reaction flask, in this case, the ammonia. To do this, close valve 5 that separates the injection manifold from the gas cylinders. vii) Open valve 7, of the cylinder of ammonia, and fill the gas system with ammonia with a pressure no higher than 3 atm. ix) Open valve 5, connecting the gas system with the injection manifold, while we continue creating a vacuum until attaining a pressure lower than 1.0 mbar again. x) Repeat the operation. This procedure is the general procedure for purging the injection manifold. It is similar for all the gases, only changing which gas cylinder valve is opened. Once the injection manifold is purged, we move on to introducing the gas desired. To introduce the desired pressure of ammonia into the reaction flask, we first have to calculate the pressure needed to introduce into the injection manifold. For this we use the formula:

$P_{1,NH3}V_I+P_{1,H2O}V_R=V_T (P_{2,NH3} +P_{2,H2O})$

where the final partial pressures are calculated:

$P_{2,NH3}=P_{1,NH3}V_I/V_T$

$P_{2, H2O}=P_{1, H2O}V_R/V_T$

If we wish to introduce 200 mbar of ammonia into the reaction flask, we must introduce mbar into the injection manifold. The vapour pressure of water ($P1,H_2O$) in the reaction flask is 32 mbar at 25ºC. To introduce the calculated pressure into the injection manifold, we again close valve 5 once the system pressure is lower than 1.0 mbar. xi) Open valve 7, corresponding to the ammonia gas cylinder, and create a pressure in the gas system no higher than 3.0 atm. to prevent the joints of the rubber tubes with the glass from coming loose. xii) Close valve 7 again. xiii) Close valve 6, which connects the vacuum pump to the injection manifold, and open valve 5 to connect the gas system with the injection manifold. Repeat the operation from point xi) until reaching the calculated pressure. xiv) Once the calculated pressure has been attained, close valve 5 and open valve 1 to connect the injection manifold with the reaction flask. xv) Close valve 1 after 3 seconds. This way we ensure that only the ammonia contained in the volume of the reaction flask dissolves into the solution. We must wait 1 hour until the ammonia is almost completely dissolved into the solution. Once 60 minutes have passed, ventilate the whole system and proceed to sample 5.0 ml of solution to measure the initial pH. xvi) To introduce the methane, proceed in the same way as for the ammonia, from point v) to xv). In this case the calculation of the pressure is different to that for ammonia. To calculate it we have to bear in mind that afterwards the nitrogen has to be introduced as well, and that, unlike the ammonia, neither dissolve in the aqueous solution. xvi) To introduce the nitrogen, proceed in the same way as for the ammonia, from point from point v) to xv).

1.4. Calculating the pressures

After the dissolution of the ammonia, ventilate the system and create a vacuum again. Our starting point is a system where the reaction flask has 32 mbar in its atmosphere (water vapour pressure at 25ºC). To calculate the pressures, we have to make a retrospective calculation, starting from the final pressure of the system (with the partial pressures that constitute it) to then calculate what pressures are necessary at each step. First we calculate the pressure of nitrogen in the injection manifold and methane in the reaction flask necessary for having desired pressures at the end:

$$V_T\left(P_{3,CH_4} + P_{3',H_2O} + P_{3,N_2}\right) = V_R\left(P_{2,CH_4} + P_{2,H_2O}\right) + V_I P_{2,N_2}$$

where the partial pressures of the nitrogen in the injection chamber and the methane in the reaction flask can be deduced:

$$P_{2,N_2} = P_{3,N_2}V_T/V_I$$

$$P_{2,CH_4} = P_{3,CH_4}V_T/V_R$$

where the final partial pressures are defined with the subscript 3, and those from the previous step on opening valve 1 that connects the reaction flask (subscript R) and the injector (subscript I) are defined with the subscript 2. For a final pressure of nitrogen of 100 mbar, we must introduce a pressure of - 172 mbar into the injection manifold using the borosilicate reaction flask. For the experiment in Teflon, introduce 136 mbar into the injector. We then need to calculate what pressure of methane is needed to be introduced into the injection manifold (P_{1,CH_4}) in order to obtain the partial pressure of methane in the reaction flask with the calculated value (P_{2,CH_4}) to obtain the final partial pressure of methane in the whole system (P_{3,CH_4}).

$$V_T\left(P_{2,CH_4} + P_{2\prime,H_2O}\right) = V_R P_{1,H_2O} + V_I P_{1,CH_4}$$

where the pressure of methane in the injector needed to obtain a final partial pressure (P_{3,CH_4}) is:

$$P_{1,CH_4} = P_{2,CH_4}V_T/V_I$$

Substituting the partial pressure of methane (P_{2,CH_4}) from equation ix) for equation vi), we are left with:

$$P_{1,CH_4} = P_{3,CH_4}V_T^2/V_I V_R$$

For a final pressure of methane of 200 mbar, an initial pressure of 820 mbar is needed in the injection manifold in the experiment with the borosilicate reaction flask. For the Teflon reaction flask, add 1022 mbar of methane into the injector.

The final total pressure of the system is:

$$P_T = P_{3,CH_4} + P_{3,H_2O} + P_{3,N_2}$$

It should be pointed out that the partial pressure of the water varies constantly throughout the process. It lessens when the pressures of the reaction flask and the injector are made equal, and returns to equilibrium with the vapour pressure when the reaction flask is isolated. The partial pressures below the value of equilibrium are differentiated by a comma with respect to the values that correspond to the vapour pressure of water. That is to say $P_{1,H_2O} = P_{2,H_2O} = P_{3,H_2O} = 32\ mbar$ and therefore

$P_{2',H_2O} = P_{3',H_2O} = 8.5\ mbar$ in the Teflon reaction flask, and 14.7 mbar in the borosilicate reaction flask. These calculations differ from the calculations carried out previously in other Miller experiments[28], which do not take into account the equilibrium of partial pressures but rather the total pressure of the system.

1.5. Operating the high-voltage generator

Before carrying out the introduction of the gases, we must ensure that the electrodes have a separation of c.a. 1.0 cm. In the case of Teflon reactor the distance of electrodes was fixed at 1.0 cm. In the case of the borosilicate reactor, the electrodes are not set to a fixed position. They were adjusted to have a separation to1.0 cm, even though precision can vary of few millimeters. Connect one of the electrodes to earth and the other in contact with the high-voltage generator. With the use of an analogue timer, the generator is set to run intermittently on and off at one hour intervals. The experiment should last 14 days, which means that the total running time of the electric arc will be 7 days. The high-voltage generator should be set to give 30000 volts.

1.6. Sample collection

A 20-ml plastic pipette has been used for the solution sample. We fill 50-ml Falcon tubes with the final solution of the experiment, using 4 Falcon tubes per experiment. Every one is for analytical purposes. One is analysed for gas-mass chromatography to learn the composition of the organic elements produced in the experiment (Grupo de Raffaelle Saladino, Italia). The second is used for elemental analysis through ICP (Zaidin). The third is used to measure the final pH of the solution (LEC). The fourth is stored. Once all the liquid has been sampled, a washing of the walls of the borosilicate reactor is necessary to recover the membrane that forms on the walls. To do this, 50 ml of milliQ water are introduced into the reactor, the walls are washed until the membrane is detached. Once most of the membrane is recovered, it is centrifuged to separate the membrane from the solution. The solid part is allowed to dry at room temperature.

1.7. Fourier-Transform Infrared Spectroscopy (FTIR)

The organic film was analysed by FTIR using a JASCO FT/ IR equipment in the region of the medium infrared spectrum (400-4000 cm^{-1}), using the attenuated total reflectance (ATR) technique, with a spectral resolution of 2.0 cm^{-1}.

1.8. Raman spectroscopy

The organic film was subjected to a pyrolytic treatment at 500 ºC for 5 hours in a muffle furnace. The pyrolyzed membrane was analysed by Raman spectroscopy on a JASCO 5100 NRS equipment. The Raman spectrum was obtained using a Red laser (785.11 nm) attenuated to 25%. The measurements were made by striking the laser for 25 seconds and making 5 accumulations between 500 and 2500 cm^{-1}.

1.9. Scaning Electron Microscopy (SEM) Energy Difusive X-Ray Scatering (EDX)

The organic film was studied by field emission scanning electron microscopy. A ZEISS SUPRA 40 VP, FESEM-EDS equipped with an Oxford energy-dispersive X-ray spectrometer (EDS), operating at 5 keV-20 KeV was used for the textural and chemical characterization of the membrane. For the elemental analyses operating conditions were set at 15 kV accelerating voltage and 7.5 mm working distance and the AZtec 3.0 SP1 EDS software was used.

1.10. Inductively coupled plasma optical emission spectrometry (ICP-OES)

The elemental composition of the collected solutions was determined by ICP-OES in a PERKIN-ELMER OPTIMA 8300 with an automatic sampler PERKIN-ELMER S10. Table S1 shows the elemental composition of the different experiments and blank solutions. Two experiment without sparking were performed in both borosilicate (BSRblank) and Teflon (TFRblank) reactors. These two experiment were used as blank.

Table S1. Chemical analysis of the solution from electrical discharge experiments.

Element	Units	BSRblank	TFRblank	BSRB	TFRB	TFBSR/B	BSR	TFR	TFBSR
Ag	µg/L	<0.005	0.007	0.247	1.03	0.142	0.053	0.046	0.408
Al	µg/L	<3	<3	<3	10	20	53	18	40
As	µg/L	<0.05	0.07	3.23	0.1	0.18	0.09	0.13	0.22
Au	µg/L	<0.002	0.002	0.003	0.016	0.002	0.018	<0.002	0.06
B	µg/L	3	<3	203	9	55	210	<3	118
Ba	µg/L	<0.05	<0.05	93	18	<0.05	9	<0.05	<0.05
Be	µg/L	<0.005	<0.005	<0.005	<0.005	<0.005	0.233	<0.005	<0.005
Bi	µg/L	<0.01	0.01	0.01	0.09	0.01	0.01	<0.01	0.02
Ca	mg/L	<0.02	0.57	7.39	0.29	0.06	0.09	0.04	0.1
Cd	µg/L	<0.005	0.021	0.927	0.336	0.37	0.018	0.02	0.185
Ce	µg/L	<0.005	0.043	0.298	0.635	0.039	4.84	0.055	0.55
Co	µg/L	0.052	0.465	0.391	0.696	0.403	0.444	0.3	1.075
Cr	µg/L	<0.5	<0.5	0.7	0.7	<0.5	0.5	0.5	<0.5
Cs	µg/L	<0.005	<0.005	0.108	<0.005	0.005	0.085	<0.005	0.049
Cu	µg/L	0.1	0.5	1.9	5.6	2.6	2.7	6.2	3.9
Fe	mg/L	<0.003	<0.003	0.077	0.028	0.006	0.012	0.011	0.007

Ga	µg/L	<0.05	<0.05	<0.05	<0.05	<0.05	<0.05	<0.05	0.18
Hf	µg/L	<0.005	0.005	0.023	0.041	0.008	0.04	<0.005	0.068
Hg	µg/L	<0.05	0.12	9.31	5.65	8.7	4.49	0.63	2.18
In	µg/L	<0.01	<0.01	0.01	0.01	0.01	0.01	0.01	0.01
K	mg/L	<0.01	0.05	0.31	0.11	0.02	0.1	0.01	3.69
La	µg/L	<0.005	0.406	5.49	3.43	0.789	102.5	0.192	1.755
Li	µg/L	<0.1	4.7	2.3	0.7	3.7	88.5	4.7	10.3
Mg	mg/L	<0.005	0.173	1.295	0.089	0.015	0.082	0.035	0.043
Mn	µg/L	0.21	0.65	4.35	0.82	0.81	1.31	0.37	1.93
Mo	µg/L	0.16	0.15	9.81	2.51	2.06	1.08	0.65	0.63
Na	mg/L	0.01	0.12	10.6	0.34	0.09	0.47	1.88	0.8
Nb	µg/L	<0.005	0.005	0.191	0.116	0.019	0.123	0.037	0.064
Ni	µg/L	<0.2	0.4	4.1	1.5	1.8	1.1	6.6	1.6
P	mg/L	<0.005	0.008	0.029	0.051	0.009	0.034	0.028	0.167
Pb	µg/L	0.07	0.08	0.39	0.3	0.06	0.96	0.06	0.17
Pd	µg/L	<0.005	<0.005	<0.005	2.2	0.097	<0.005	0.019	0.017
Pt	µg/L	<0.005	<0.005	0.011	0.085	0.035	0.016	0.014	0.027
Rb	µg/L	<0.01	0.05	0.96	0.09	0.02	0.59	<0.01	0.6
Re	µg/L	<0.002	0.005	0.027	0.08	0.013	0.405	0.015	0.084
S	mg/L	<0.2	<0.2	<0.2	<0.2	<0.2	0.2	<0.2	<0.2
Sb	µg/L	<0.01	0.01	0.09	0.06	0.03	0.07	0.07	0.02
Sc	µg/L	<0.01	<0.01	1.64	0.39	0.13	0.37	0.13	0.25
Se	µg/L	<0.05	<0.05	1.81	1.96	0.81	1.82	0.2	0.6
Si	mg/L	0.04	0.08	39.3	n.d.	0.7	2.6	n.d.	1.44
Sn	µg/L	<0.05	0.12	0.76	1.12	0.2	0.22	0.1	6.89
Sr	µg/L	0.05	2.97	9.41	1.9	0.54	1.73	0.4	1.23
Ta	µg/L	<0.01	<0.01	0.07	0.06	0.02	0.02	0.06	0.04
Te	µg/L	<0.01	<0.01	0.08	0.03	0.03	0.01	0.11	0.05
Th	µg/L	<0.005	0.018	0.029	0.291	0.006	0.135	0.008	0.16
Ti	µg/L	<0.2	<0.2	10	<0.2	<0.2	<0.2	<0.2	<0.2
Tl	µg/L	<0.002	0.032	0.017	0.005	0.005	0.012	<0.002	0.181
U	µg/L	<0.002	0.031	0.057	0.522	0.029	0.33	0.011	0.216
V	µg/L	<0.05	<0.05	0.38	0.2	0.19	0.21	0.08	0.13
W	µg/L	9.43	204	1210	3640	672	18450	756	3760
Y	µg/L	<0.005	0.008	0.037	0.037	<0.005	0.067	<0.005	0.027
Zn	µg/L	1.6	11.3	30	15.5	36.8	104.5	32.6	81.1
Zr	µg/L	<0.02	0.04	1.0	1.55	0.58	1.74	0.03	1.56

BSR-blank: Borosilicate reactor in unbuffered conditions without sparking; TFRblank: Teflon reactor in unbuffered conditions without sparking; BRS and BRSB: Borosilicate reactor in unbuffered and buffered conditions, respectively; TFR and TFRB: Teflon reactor in unbuffered and buffered conditions, respectively; TFBSR and TFBSR/B: Teflon reactor in unbuffered and buffered conditions, respectively, and in the presence of borosilicate bits.

Table S2. Synthesis of prebiotic chemical precursors, amino acids, carboxylic acids, nucleobases and aromatic and heteroaromatic miscellanea by Urey-Miller electric-discharge in buffered conditions; Products are grouped by chemical class similarity and reactor type.

Entry	Class	Compound	BRSB			TFRB			TFBSR/B		
			Rt (min)	Yield (µg/mg)	Total yield[h]	Rt (min)	Yield (µg/mg)	Total yield[i]	Rt (min)	Yield (µg/mg)	Total yield[l]
1	**CCP**	Formamide (**1**)	5.836 [b]	44,18(e)	1,85	5.927[b]	13,90(g)	0,32	5.751[b]	57,24(e)	1,77
2		Formic acid (**2**)(m)	3.341[a]	66,81(e)	2,80	3.273[a]	14,03(f)	0,32	3.265[a]	27,24(e)	0,84
3		Urea (**3**)	10.286[b]	11,97(g) (1,91(g))	0,50 (0,08)	10.173[b]	3,36(g)	0,07	10.178[b]	16,43(f)	0,51
4		DAMN (**4**)	-	-	-	-	-	-	-	-	-
5	**Amino acids and derivatives**	Glycine (**5**)	2.664[a]	17,04(f) (13,40(g))	0,71 (0,56)	-	-	-	2.668[a]	0,62(g)	0,02
6		Alanine (**6**)	2.977[b]	37,43(e)	1,57	-	-	-	-	-	-
7		Valine (**7**)	17.268[b]	4,93(g)	0,20	-	-	-	17.264[b]	1,87(g)	0,05
8		Leucine (**8**)	13.868[b]	6,23(g)	0,26	-	-	-	13.869[b]	0,55(g)	0,02
9		Proline (**9**)	12.967[b]	17,95(f)	0,75	13.045[b]	0,42(g)	0,01	12.944[b]	9,89(g)	0,31
10		Serine (**10**)	14.101[c]	2,63(g)	0,11	-	-		14.086[c]	0,63(g)	0,02
11		Asparagine (**11**)	16.692[c]	3,18(g)	0,13	16.650[c]	0,03(g)	0,001	16.671[c]	0,17(g)	0,005
12		Aspartic ac. (**12**)	17.329[c]	4,86(g)	0,20	-	-		17.322[c]	1,59(g)	0,05
13		Glutamic ac. (**13**)	17.962[c]	1,64(g)	0,07	-	-		17.959[c]	1,16(g)	0,03
14		Lysine (**14**)	18.915[d]	10,86(g)	0,46	18.910[d]	2,88(g)	0,07	18.902[d]	2,39(g)	0,07
15		Histidine (**15**)	25.004[b]	2,99(g)	0,12	24.990[b]	3,95(g)	0,09	25.000[b]	2,64(g)	0,08
16		β-Alanine (**16**)	10.659[b]	3,21(g)	0,13	-	-	-	10.607[b]	0,53(g)	0,02
17		Isovaline (**17**)	17.329[b]	Traces	Traces	-	-	-	17.322[b]	Traces	Traces
18		α-NH_2-isobutyric ac. (**18**)	17.363[b]	Traces	Traces	-	-	-	17.347[b]	Traces	Traces
19		γ-NH_2-butiric ac. (**19**)	15.499[c]	8,44(g)	0,35	15.482[c]	17,08(f)	0,39	15.481[c]	2,63(g)	0,08
20		*N*-fGlycine (**20**)	8.348[b]	25,58(e)	1,07	8.339[b]	2,74(g)	0,06	8.300[b]	16,06(f)	0,49
21		*N*-fLeucine (**21**)	12.204[a]	8,37(g)	0,35	-	-	-	12.173[a]	13,75(g)	0,42
22		Glycylglycine (**22**)	9.258[a]	7,11(g)	0,29	-	-	-	9.196[a]	6,05(g)	0,18
23		1-Butanamine (**23**)	2.469	33,21(e)	1,39	2.431	33,50(e)	0,77	2.434	34,53(e)	1,07
24		Isobutylamine (**24**)	10.930	0,59(g)	0,02	-	-	-	10.924	0,26(g)	0,008
25	**Carboxylic acids**	Glycolic ac. (**25**)	6.307[b]	7,49(g)	0,31	-	-		6.224[b]	0,43(g)	0,01
26		Oxalic ac. (**26**)	7.104[b]	11,69(g)	0,49	6.984[b]	1,04(g)	0,02	6.962[b]	7,77(g)	0,24
27		Pyruvic ac. (**27**)	5.775[b]	5,45(g)	0,23	4.415 [a]	7,66(g)	0,17	5.555[b]	8,37(g)	0,25
28		Lactic ac. (**28**)	8.214[c]	0,52(g)(0, 81(g)) [1,04(g)]	0,02 (0,03) [0,04]	8.024[c]	1,28(g)	0,03	8.016[c]	1,15(g)	0,03
29		Maleic ac. (**29**)	3.542[b]	1,60(g)	0,06	3.485[b]	0,90(g)	0,02	3.519[b]	6,31(g)	0,19
30		Malic ac. (**30**)	6.685[c]	0,87(g)	0,04	-	-	-	6.675[c]	0,31(g)	0,01
31		Oxaloacetic ac. (**31**)	6.181[c]	1,63(g)	0,07	-	-	-	6.183[c]	0,07(g)	0,002
32		2-Ketoglutaric ac. (**32**)	11.361[c]	0,49 (g)	0,02	-	-	-	11.291[c]	1,35(g)	0,04
33		Hexanoic ac (**33**)	7.848[a]	0,38(g)	0,01	7.797[a]	traces	-	7.756[a]	traces	traces
34		Nonanoic ac. (**34**)	16.253[a]	3,33(g)	0,134	16.256[a]	1,03(g)	0,02	16.252[a]	0,3(g)	0,01
35		Gentisic ac. (**35**)	24.752[c]	3,20(g)	0,13	-	-		24.783[c]	0,43(g)	0,01
36	**Nucleobases**	Adenine (**36**)	5.382[a]	5,00(g) (3,53(g)) [7,15(g)]	0,21 (0,15) [0,30]	5.269[a]	3,29(g)	0,07	5.319[a]	2,55(g)	0,08
37		Guanine (**37**)	8.609[c]	0,33(g)	0,01	-	-	-	8.552[c]	0,91(g)	0,03
38		Uracil (**38**)	12.872[b]	5,14(g)	0,21	-	-	-	12.859[b]	0,79(g)	0,02
39		Cytosine (**39**)	4.326[a]	3,29(g) (3,39(g)) [7,88(g)]	0,13 (0,14) [0,33]	4.247[a]	2,73(g)	0,06	4.222[a]	1,58(g)	0,05
40		Thymine (**40**)	9.870[b]	0,25(g)	0,01	9.863[b]	1,32(g)	0,03	-	-	

41	**Miscellanea**	Parabanic ac. (**41**)	12.612[b]	4,38(g)	0,18	12.595[b]	0,08(g)	0,001	12.595[b]	2,32(g)	0,07
42		3,5-diNH2-1,2,4-triazole (**42**)	11.063[c]	3,70(g)	0,15	11.050[c]	1,03(g)	0,02	11.016[c]	3,55(g)	0,11
43		1H-Indole-3-methanamine (**43**)	5.590	0.08(g)	0,003	5.587	3,71(g)	0,08	-	-	-
44		9-Acridinamine (**44**)	14.484	2,18(g)	0,09	-	-	-	14.484	0,62(g)	0,02
45		Hydroxy-naphthalene (**45**)	10.795[a]	1,10(g)	0,05	-	-	-	10.740[a]	0,21(g)	0,006
46		1,8 Dihydroxy-naphthalene (**46**)	13.764[b]	0,32(g)	0,01	-	-	-	13.762[b]	0,14(g)	0,004
47		Methyl-naphtalene (**47**)	14.296	10,87(g)	0,45	-	-	-	14.279	4,02(g)	0,12
48		Acenaphthylene (**48**)	5.128	4,32(g)	0,18	5.052	2,32(g)	0,05	-	-	-

BRSB: Borosilicate reactor in buffered conditions; TFRB: Teflon reactor in buffered conditions; TFBSR/B: Teflon reactor in buffered conditions and in the presence of borosilicate bits. DAMN: CCP: C-1 chemical precursors. Diaminomaleonitrile. *N*-fGlycine: *N*-formylglycine. *N*-fLeucine: *N*-formylleucine. The yield is defined as μg of product per 1.0 mg of the reaction crude. Rt retention time (min). Products have been detected with a different degree of silylation: [a] mono-silyl derivative; [b] di-silyl derivative; [c] tri-silyl derivative; [d] tetra-silyl derivative. Values in round brackets are referred to the yields of the products in the organic film without acidic treatment. Values in square brackets are referred to the yields of the products in the organic film after acidic treatment. (e) Data are the mean values of three experiments with SD of ± 0.03μg. (f) Data are the mean values of three experiments with SD of ± 0.02 μg. (g) Data are the mean values of three experiments with SD of ± 0.01 μg. (h) Mg of product *per* 38 mg of crude. (i) Mg of product *per* 23 mg of crude. (l) Mg of product *per* 31 mg of crude. (m) Note that the yield of formic acid may be altered by the freeze-drying process.

Table S3. Synthesis of prebiotic chemical precursors, amino acids, carboxylic acids, nucleobases and aromatic and heteroaromatic miscellanea by Urey-Miller electric-discharge in unbuffered conditions; Products are grouped by chemical class similarity and reactor type.

Entry	Class	Compound	BRS			TFR			TFBSR		
			Rt (min)	Yield (μg/mg)	Total yield[h]	Rt (min)	Yield (μg/mg)	Total yield[i]	Rt (min)	Yield (μg/mg)	Total yield[l]
1	**CCP**	Formamide (**1**)	5.738[b]	38,89(e)	1,63	6.039[b]	3,4(g)	0,09	5.923[b]	24,59(f)	0,93
2		Formic acid (**2**)(m)	3.267[a]	44,25(e)	1,85	3.268[a]	20,96(f)	0,58	3.381[a]	23,52(f)	0,89
3		Urea (**3**)	10.134[b]	38,32(e)	1,61	10.141[b]	7,39(g)	0,21	10.348[b]	37,77(e)	1,43
4		DAMN (**4**)	9.325[a]	8,18(g)	0,34	9.332[a]	3.70(g)	0,10	9.327 [a]	4,30(g)	0,16
5	**Amino acids and derivatives**	Glycine (**5**)	11.497[b]	4,02(g)	0,17	2.664[a]	2,82(g)	0,07	11.583[b]	4,39(g)	0,16
6		Alanine (**6**)	2.931[b]	2,92(g)	0,12	-	-	-	3.008[b]	3,48(g)	0,13
7		Valine (**7**)	-	-	-	-	-	-	-	-	-
8		Leucine (**8**)	13.811[b]	2,99(g)	0,12	13.824[b]	5,29(g)	0,15	-	-	-
9		Proline (**9**)	12.915[b]	6,79(g)	0,28	12.911[b]	4,15(g)	0,12	13.002[b]	9,19(g)	0,35
10		Serine (**10**)	14.042[c]	2,99(g)	0,12	14.053[c]	2,31(g)	0,06	14.124[c]	12,48(g)	0,47
11		Asparagine (**11**)	16.790[c]	5,29(g)	0,22	-	-	-	16.792[c]	0,5(g)	0,02
12		Aspartic ac. (**12**)	-	-	-	-	-	-	-	-	-
13		Glutamic ac. (**13**)	17.859[c]	1,20(g)	0,05	-	-	-	17.851[c]	0,5(g)	0,02
14		Lysine (**14**)	18.872[d]	30,71(f)	1,29	18.876[d]	12,33(g)	0,35	18.931[d]	15,17(g)	0,57
15		Histidine (**15**)	24.974[b]	7,46(g)	0,31	24.986[b]	7,18(g)	0,20	25.020[b]	8,21(g)	0,31
16		β-Alanine (**16**)	10.588[b]	2,52(g)	0,10	10.571[b]	0,5(g)	0,01	10.685[b]	5,88(g)	0,22

17		Isovaline (**17**)	17.281[b]	Traces	Traces	-	-	-	17.331[b]	Traces	Traces
18		α-NH_2-isobutyric ac. (**18**)	17.306[b]	Traces	Traces	17.322[b]	Traces	Traces	-	-	-
19		γ-NH_2-butiric ac. (**19**)	-	-	-	-	-	-	-	-	-
20		*N*-fGlycine (**20**)	8.258[b]	27,02(f)	1,13	8.262[b]	13,11(g)	0,36	8.387[b]	5,36(g)	0,20
21		*N*-fLeucine (**21**)	12.141[a]	17,28(f)	0,72	12.144[a]	3,51(g)	0,09	12.244[a]	4,87(g)	0,18
22		Glycylglycine (**22**)	9.169[a]	10,50(g)	0,44	-	-	-	9.291[a]	14,68(g)	0,56
23		1-Butanamine (**23**)	2.469	77,07(e)	3,23	2.440	78,19(e)	2,18	2.506	69,06(e)	2,62
24		Isobutylamine (**24**)	-	-	-	-	-	-	-	-	-
25	**Carboxylic acids**	Glycolic ac. (**25**)	6.225[b]	0,27(g)	0,01	-	-	-	6.354[b]	5,87(g)	0,22
26		Oxalic ac. (**26**)	6.961[b]	4,75(g)	0,19	6.962[b]	6,94(g)	0,19	7.140[b]	9,97(g)	0,37
27		Pyruvic ac. (**27**)	5.722 [b]	8,29(g)	0,35	-	-	-	5.752 [b]	6,86(g)	0,26
28		Lactic ac. (**28**)	8.206[c]	6,33(g)	0,26	8.001[c]	6,87(g)	0,19	8.228[c]	6,07(g)	0,23
29		Maleic ac. (**29**)	3.510[b]	4,53(g)	0,19	3.519[b]	1,93(g)	0,05	3.637[b]	4,95(g)	0,18
30		Malic ac. (**30**)	-	-	-	-	-	-	-	-	-
31		Oxaloacetic ac. (**31**)	6.183[c]	1,49(g)	0,06	-	-	-	6.218[c]	8,20(g)	0,31
32		2-Ketoglutaric ac. (**32**)	-	-	-	-	-	-	11.441[c]	12,25(g)	0,46
33		Hexanoic ac (**33**)	-	-		-	-	-	-	-	-
34		Nonanoic ac. (**34**)	16.208[a]	18,94(f)	0,79	16.218[a]	12,47(g)	0,35	16.275[a]	19,98(f)	0,76
35		Gentisic ac. (**35**)	24.785[c]	1,70(g)	0,07	-	-	-	24.791[c]	6,74(g)	0,26
36	**Nucleobases**	Adenine (**36**)	5.386[a]	4,62(g)	0,19	5.246[a]	4,69(g)	0,13	5.403[a]	3,87(g)	0,15
37		Guanine (**37**)	8.569[c]	0,82(g)	0,03	-	-	-	-	-	-
38		Uracil (**38**)	12.903[b]	2,44(g)	0,10	-	-	-	12.992[b]	2,94(g)	0,11
39		Cytosine (**39**)	4.345[a]	7,87(g)	0,33	4.206[a]	Traces	Traces	4.332[a]	7,21(g)	0,27
40		Thymine (**40**)	9.871[b]	0,55 g)	0,02	-	-	-	-	-	-
41	**Miscellanea**	Parabanic ac. (**41**)	-	-	-	12.541[b]	25,58(f)	0,72	-	-	-
42		3,5-diNH_2-1,2,4-triazole (**42**)	11.001[c]	5,74(g)	0,24	11.035[c]	3,76(g)	0,10	11.105[c]	6,30(g)	0,24
43		1H-Indole-3-methanamine (**43**)	-	-	-	-	-	-	-	-	-
44		9-Acridinamine (**44**)	-	-	-	-	-	-	14.519	4,57(g)	0,17
45		Hydroxy-naphthalene (**45**)	10.812[a]	5,32(g)	0,22	10.800[a]	7,50(g)	0,21	10.814[a]	3,30(g)	0,12
46		1,8 Dihydroxy-naphthalene (**46**)	-	-	-	-	-	-	-	-	-
47		Methyl-naphtalene (**47**)	-	-	-	-	-	-	-	-	-
48		Acenaphthylene (**48**)	5.041	12,01(g)	0,50	5.040	11,41(g)	0,32	5.201	19,41(f)	0,74

BRS: Borosilicate reactor in unbuffered conditions; TFR: Teflon reactor in unbuffered conditions; TFBSR: Teflon reactor in unbuffered conditions and in the presence of borosilicate bits. DAMN: CCP: C-1 chemical precursors. Diaminomaleonitrile. *N*-fGlycine: *N*-formylglycine. *N*-fLeucine: *N*-formylleucine. Products have been detected with a different degree of silylation: [a] mono-silyl derivative; [b] di-silyl derivative; [c] tri-silyl derivative; [d] tetra-silyl derivative. The yield is defined as μg of product per 1.0 mg of crude and as mg of product per total amount of the reaction crude. Rt retention time (min). (e) Data are the mean values of three experiments with SD of ± 0.03 μg. (f) Data are the mean values of three experiments with SD of ± 0.02 μg. (g) Data are the mean values of three experiments with SD of ± 0.01 μg. (h) Mg of product *per* 42 mg of crude. (i) Mg of product *per* 28 mg of crude. (l) Mg of product *per* 38 mg of crude. (m) Note that the yield of formic acid may be altered by the freeze-drying process.

SI #2 Mass to charge (m/z) ratio values and relative peak abundances of products (1-48).

Table S4: Ion abundance and MS fragmentation profiles of compounds **1-48.**

Product	m/z
Formamide[(b)] (**1**)	189 [M+ 2TMS] (43), 174 [M+ 2TMS-Me] (10), 116 [M+ TMS] (5), 101 [M+ TMS-Me] (8)
Formic acid[(a)] (**2**)	118 [M+ TMS] (25)
Urea[(b)] (**3**)	204 [M+ 2TMS] (5), 189 [M+ 2TMS-Me] (73), 174 [M+ 2TMS-2Me] (10), 132 [M+ TMS] (7)
DAMN[(a)] (**4**)	180 [M+ TMS] (18)
Glycine[(a)] (**5**)	147 [M+ TMS] (100), 132 [M+ TMS-Me] (7)
Glycine[(b)] (**5**)	219 [M+ 2TMS] (48), 204 [M+ 2TMS-Me] (23), 147 [M+ TMS] (100), 132 [M+ TMS-Me] (8)
Alanine[(b)] (**6**)	218 [M+ 2TMS-Me] (3), 146 [M+ TMS-Me] (40)
Valine[(b)] (**7**)	246 [M+ 2TMS-Me] (2), 218 [M+ 2TMS-3Me] (7), 189 [M+ TMS] (37)
Leucine[(c)] (**8**)	275 [M+ 2TMS] (2), 260 [M+ 2TMS-Me] (5), 203 [M+ TMS] (6), 188 [M+ TMS-Me] (7)
Proline[(b)] (**9**)	259 [M+ 2TMS] (2), 244 [M+ 2TMS-Me] (2), 187 [M+ TMS] (3).
Serine[(c)] (**10**)	306 [M+ 3TMS-Me] (5), 219 [M+ 2TMS-2Me] (20), 204 [M+ 2TMS-3Me] (53)
Asparagine[(c)] (**11**)	348 [M+ 3TMS] (9), 232 [M+ 2TMS-3Me] (20), 188 [M+ TMS-Me] (40)
Aspartic ac.[(c)] (**12**)	349 [M+ 3TMS] (2), 334 [M+ 3TMS-Me] (3), 232 [M+ 2TMS-3Me] (42), 205[M+ TMS] (5)
Glutamic ac.[(c)] (**13**)	363 [M+ 3TMS] (2)
Lysine[(d)] (**14**)	434 [M+ 4TMS] (10), 317 [M+ 3TMS-3Me] (11)
Histidine[(b)] (**15**)	299 [M+ 2TMS] (5)
β-Alanine[(b)] (**16**)	233 [M+ 2TMS] (2)
Isovaline[(b)] (**17**)	261 [M+ 2TMS] (4), 231 [M+ 2TMS-2Me] (5), 189 [M+ TMS] (4), 159 [M+ TMS-2Me] (23)
α-NH_2-isobutyric ac. [(b)] (**18**)	217 [M+ 2TMS-2Me] (5)
γ-NH_2-butiric ac. [(c)] (**19**)	304 [M+ 3TMS-Me] (13), 246 [M+ 2TMS] (7), 189 [M+ 2TMS-2Me] (5), 174 [M+ TMS] (100)
N-fGlycine[(b)] (**20**)	247 [M+ 2TMS] (8), 232 [M+ 2TMS-Me] (12), 175 [M+ TMS] (100)
N-fLeucine[(a)] (**21**)	231 [M+ TMS] (90), 216 [M+ TMS-Me] (12)
Glycylglycine[(a)] (**22**)	204 [M+ TMS] (47), 189 [M+ TMS-Me] (10), 132 [M] (32)
1-Butanamine(**23**)	73 [M] (100)
Isobutylamine (**24**)	73 [M] (100)
Glycolic ac. [(b)] (**25**)	221 [M+2TMS] (30), 191 [M+ 2TMS-2Me] (100)
Oxalic ac.[(b)] (**26**)	219 [M+ 2TMS-Me] (10)
Pyruvic ac.[(b)] (**27**)	217 [M+ 2TMS-Me] (40)

Lactic ac.[c] (**28**)	291 [M+3TMS-Me] (12), 234 [M+ 2TMS] (7), 219 [M+ 2TMS-Me] (30)
Maleic ac.[b] (**29**)	245 [M+ 2TMS-Me] (23)
Malic ac.[c] (**30**)	335 [M+ 3TMS-Me] (14)
Oxaloacetic ac.[c] (**31**)	348 [M+ 3TMS] (2), 333[M+ 3TMS-Me] (90)
2-Ketoglutaric ac.[c] (**32**)	362 [M+ 3TMS] (5), 347[M+ 3TMS-Me] (92)
Hexanoic ac[a] (**33**)	188 [M+ TMS] (25), 173[M+ TMS-Me] (43)
Nonanoic ac.[a] (**34**)	230 [M+ TMS] (75)
Gentisic ac.[c] (**35**)	370 [M+ 3TMS] (67), 355[M+ 3TMS-Me] (18), 283[M+ 2TMS-Me] (10)
Adenine[a] (**36**)	207 [M+ TMS] (42), 192[M+ TMS-Me] (100)
Guanine[c] (**37**)	367 [M+ 3TMS] (38), 352[M+ 3TMS-Me] (100)
Uracil[b] (**38**)	256 [M+ 2TMS] (58), 241 [M+ 2TMS-Me] (100), 112 [M] (12)
Cytosine[a] (**39**)	183 [M+ TMS] (40), 168[M+ TMS-Me] (100)
Thymine[b] (**40**)	270 [M+ 2TMS] (45), 255 [M+ 2TMS-Me] (100)
Parabanic[b] ac. (**41**)	258 [M+ 2TMS] (18), 243 [M+ 2TMS-Me] (65)
3,5-diNH_2-1,2,4-triazole[c] (**42**)	315 [M+ 3TMS] (100), 300 [M+ 3TMS-Me] (62), 243 [M+ 2TMS] (20), 228 [M+ 2TMS-Me] (34), 213 [M+ 2TMS-2Me] (61), 171 [M+ TMS] (63), 156 [M+ TMS-Me] (17), 141[M+ TMS-2Me] (12), 99[M] (28)
1H-Indole-3-methanamine(**43**)	146 [M] (100)
9-Acridinamine(**44**)	194 [M] (78)
Hydroxy-naphthalene[a] (**45**)	216 [M+ TMS] (100), 201 [M+ TMS-Me] (75), 186 [M+ TMS-2Me] (50)
1,8-Dihydroxy naphthalene[b] (**46**)	304 [M+ 2TMS] (100), 289 [M+ 2TMS-Me] (7), 217 [M+ TMS-Me] (23)
Methyl-naphtalene (**47**)	142 [M] (100)
Acenaphthylene (**48**)	152 [M] (100)

DAMN: Diaminomaleonitrile. *N*-fGlycine: *N*-formylglycine. *N*-fLeucine: *N*-formylleucine. The abundance of ions is shown in round brackets. Products have been detected with a different degree of silylation: [a] mono-silyl derivative; [b] di-silyl derivative; [c] tri-silyl derivative; [d] tetra-silyl derivative.

SI #3 Gas-chromatograms of the electric-discharge experiments and representative standards

BSRB: Borosilicate reactor in buffered conditions.

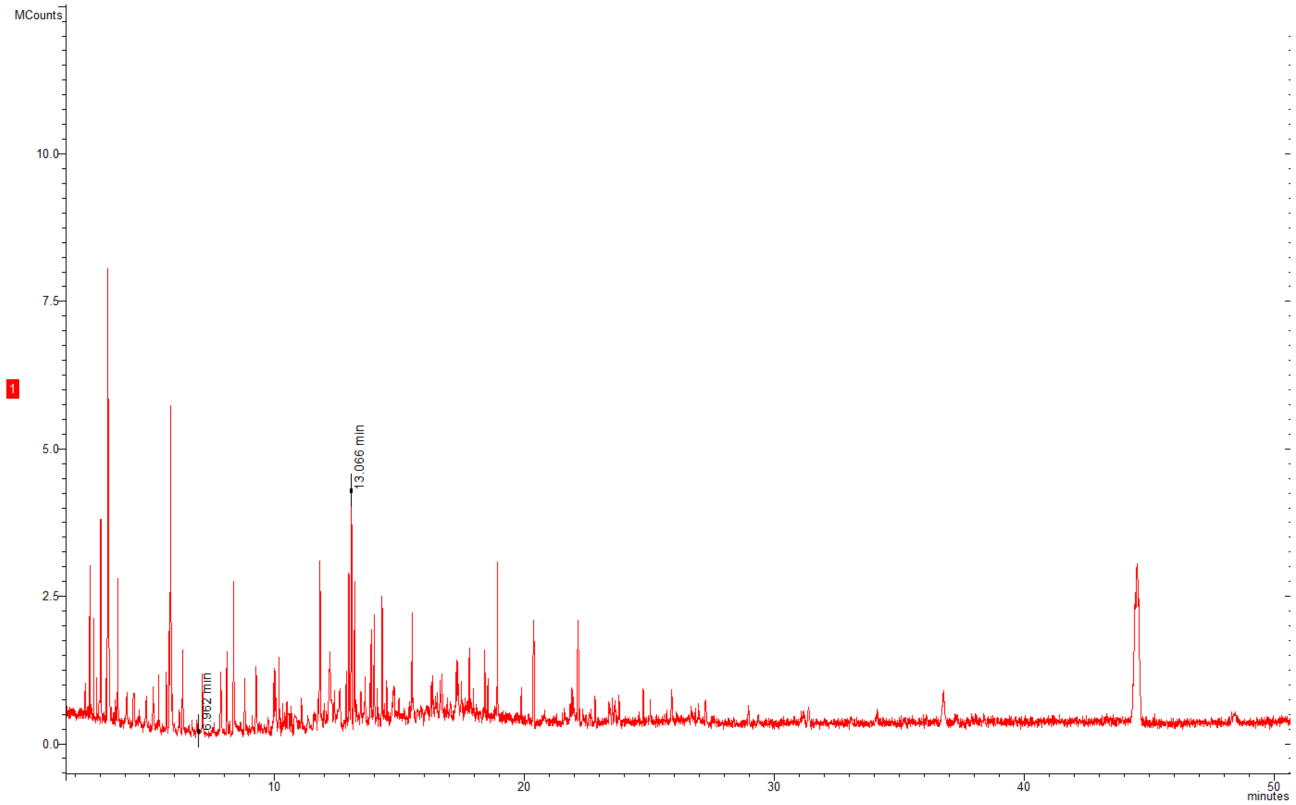

TFRB: Teflon reactor in buffered conditions.

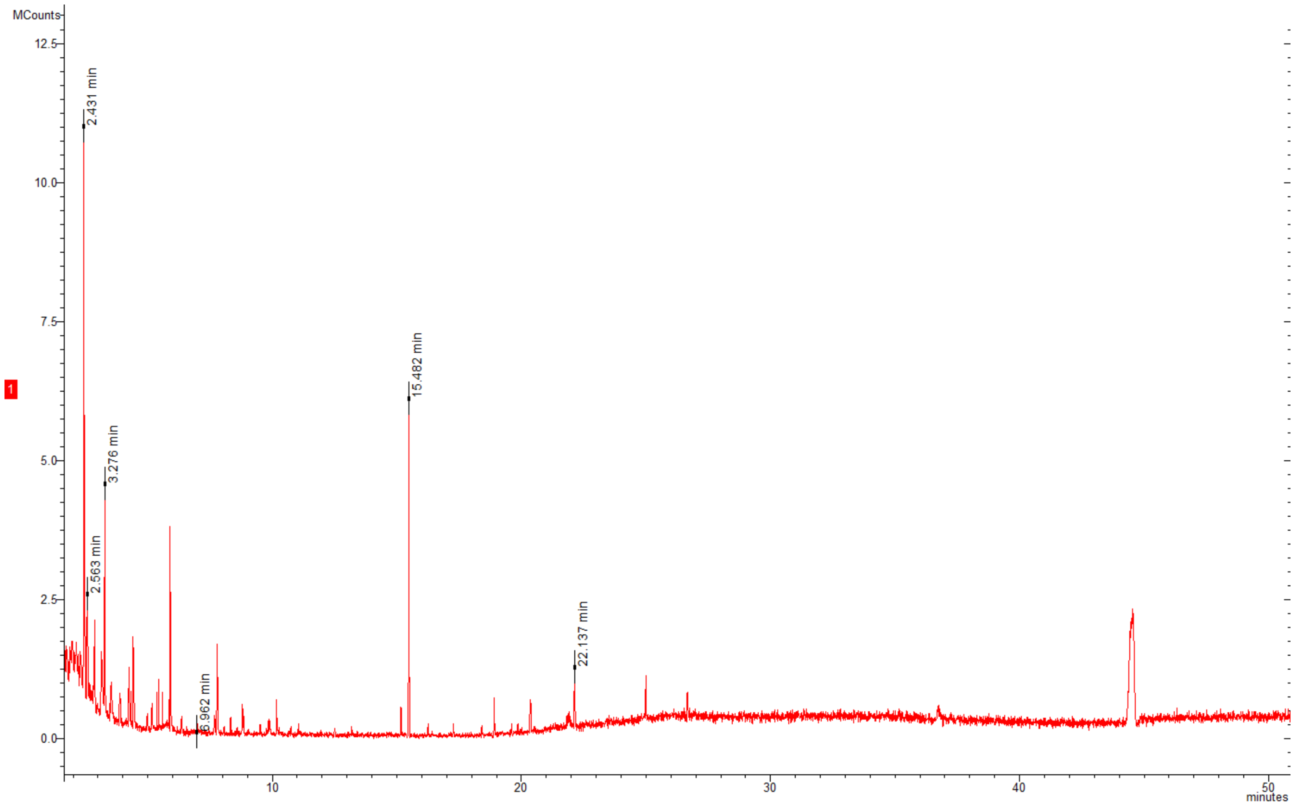

TFBSR/B: Teflon reactor in buffered conditions and in the presence of borosilicate bits.

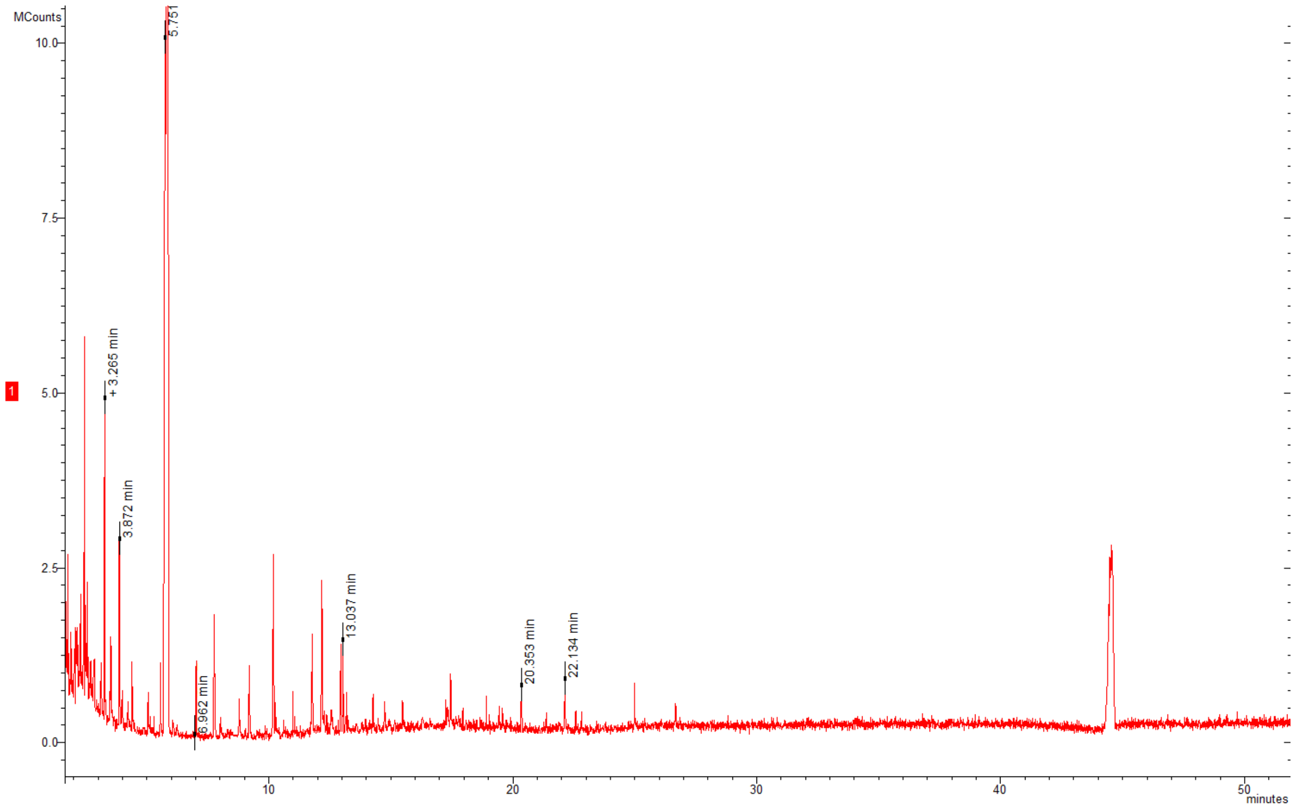

BSR: Borosilicate reactor in unbuffered conditions

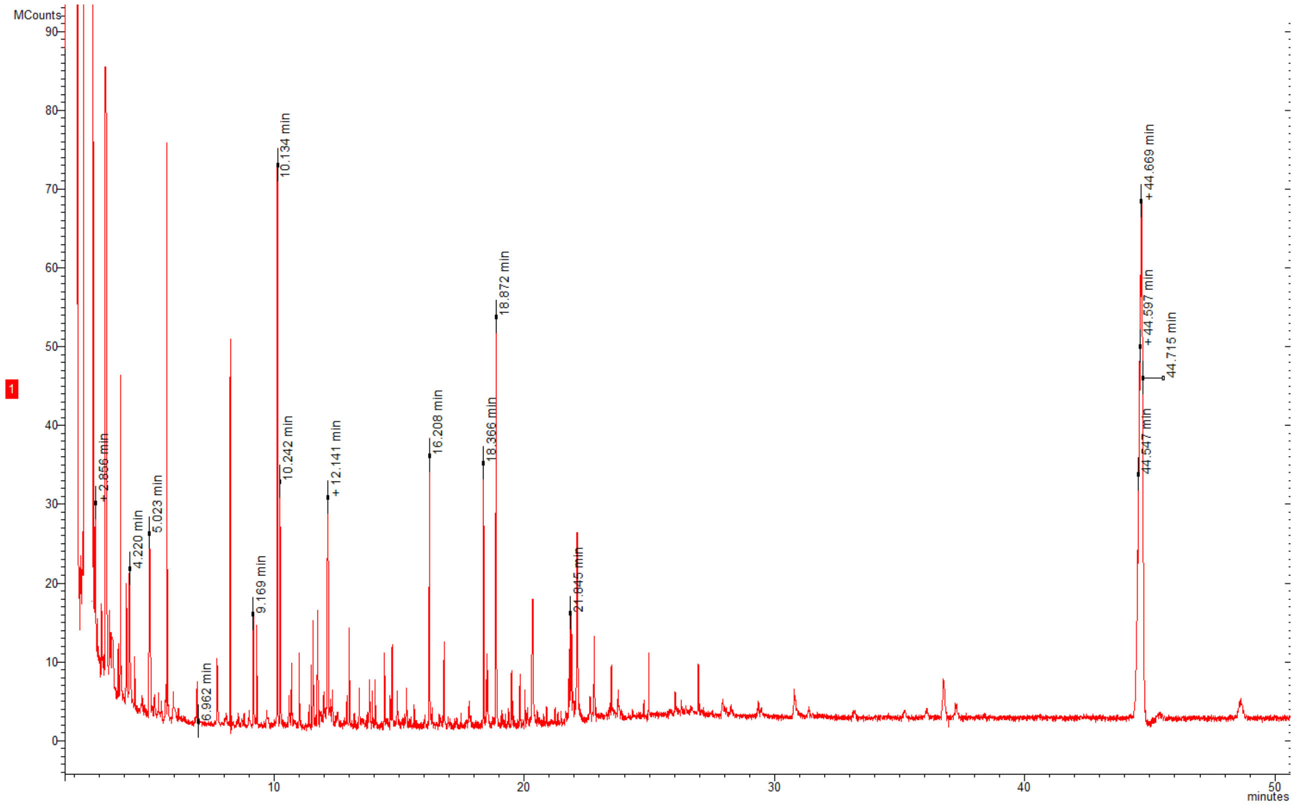

TFR: Teflon reactor in unbuffered conditions

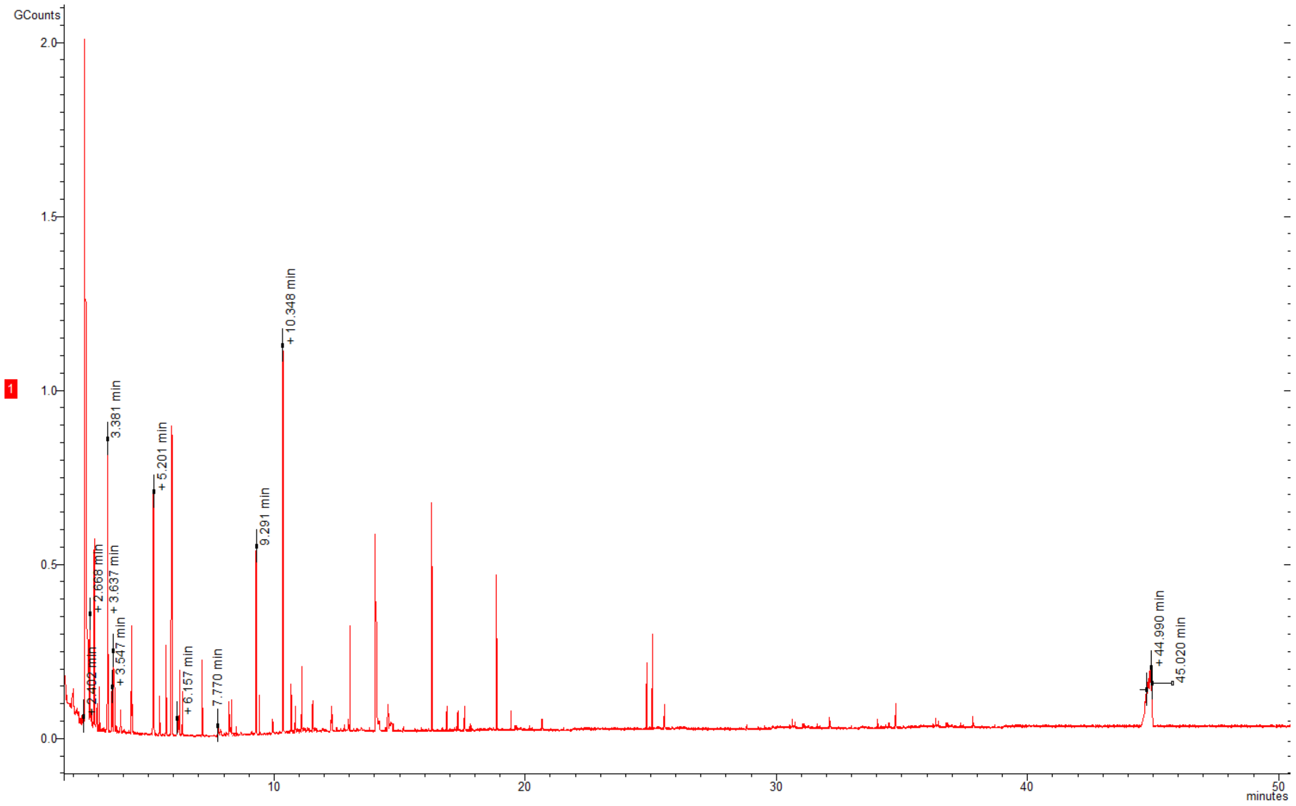

TFBSR: Teflon reactor in unbuffered conditions and in the presence of borosilicate bits.

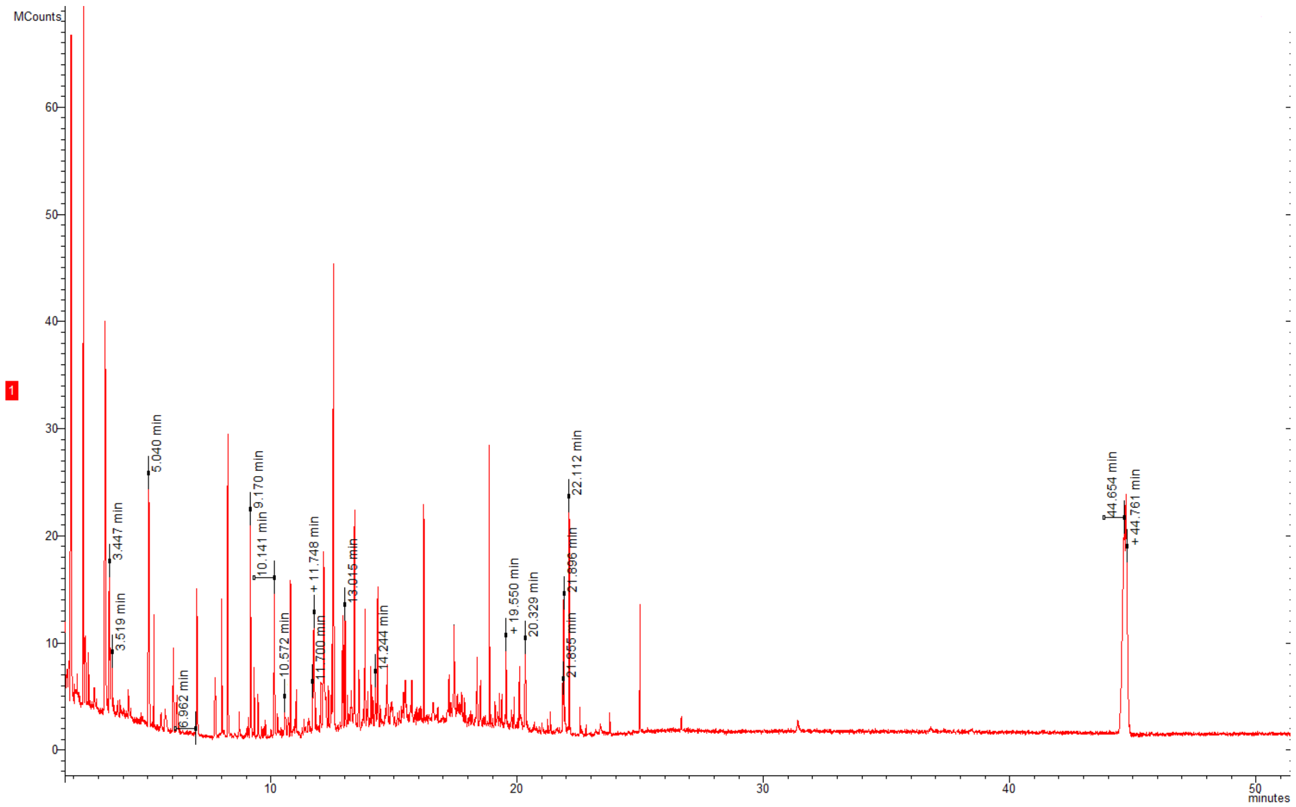

BSRB before co-injection run (magnification from 17.0 min to 18.2 min).

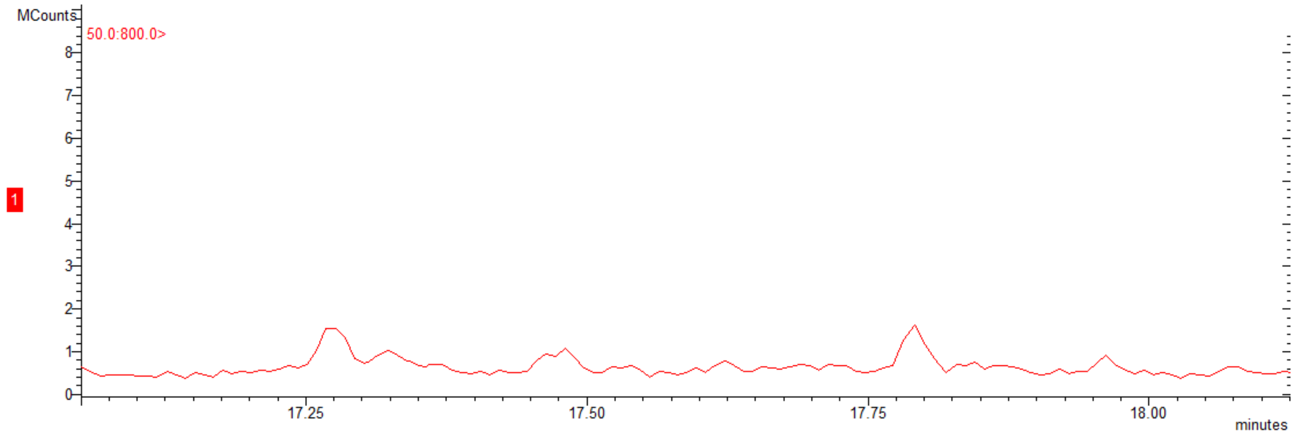

Co-injection run of BSRB+ 0.1 µmol of valine std. (magnification from 16.5 min to 18.5 min).

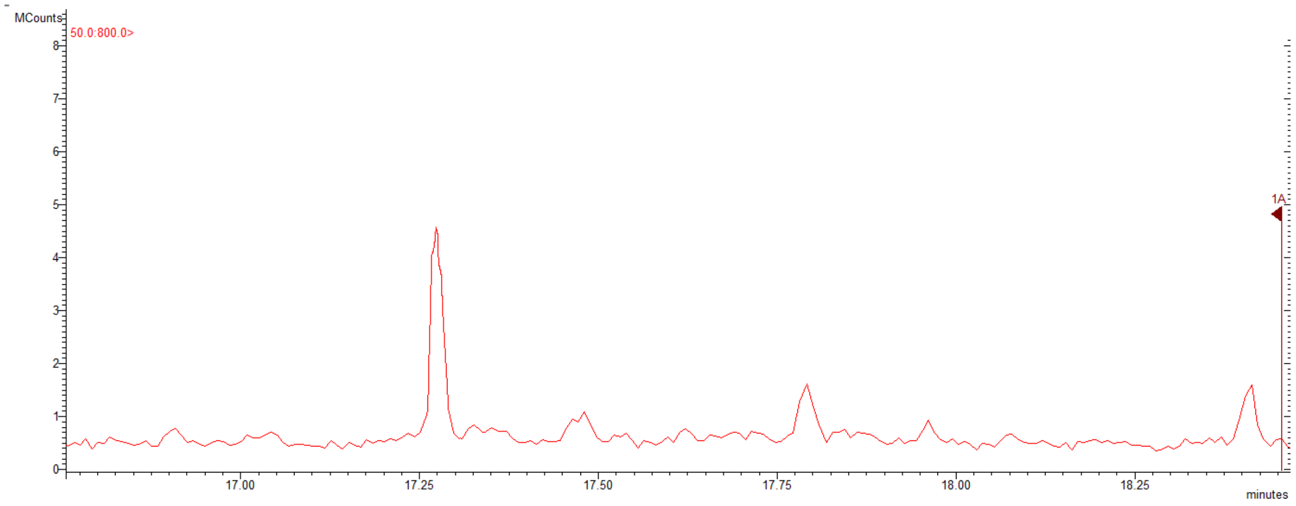

Co-injection run of BSRB+ 0.1 µmol isovaline std. (magnification from 16.5 min to 18.5 min).

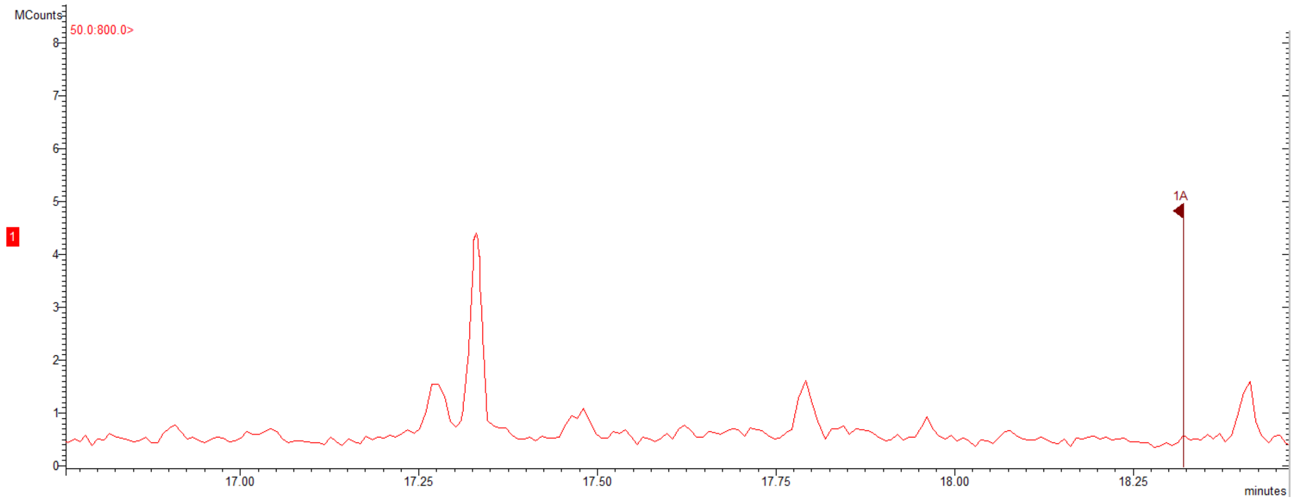

Co-injection run of BSRB+ 0.1 μmol α–NH_2-isobutyric ac. std. (magnification from 16.5 min to 18.5 min).

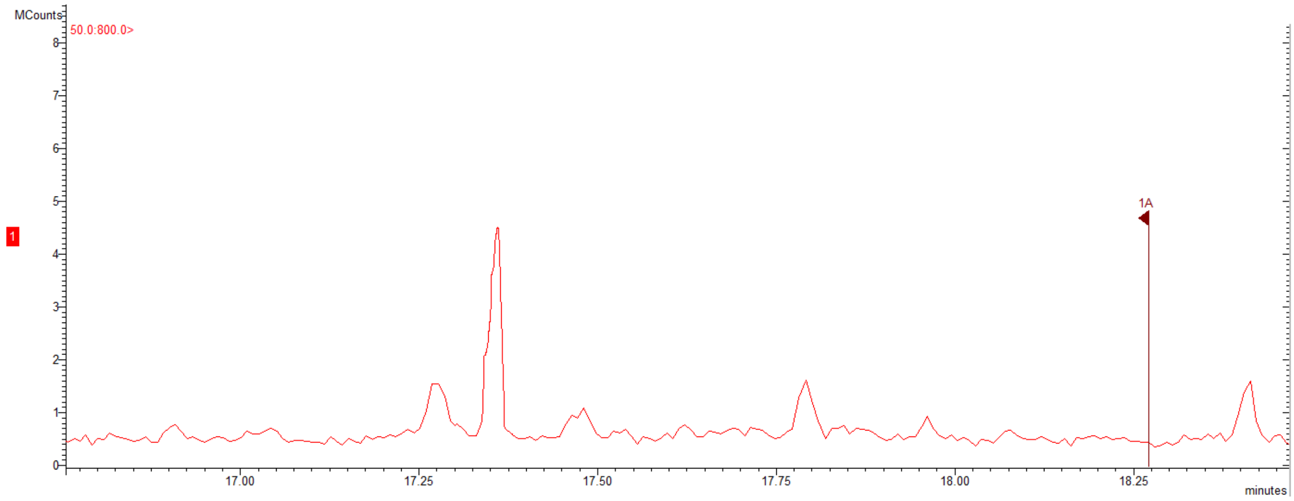

BSRB before co-injection run (magnification from 14.50 min to 16.75 min).

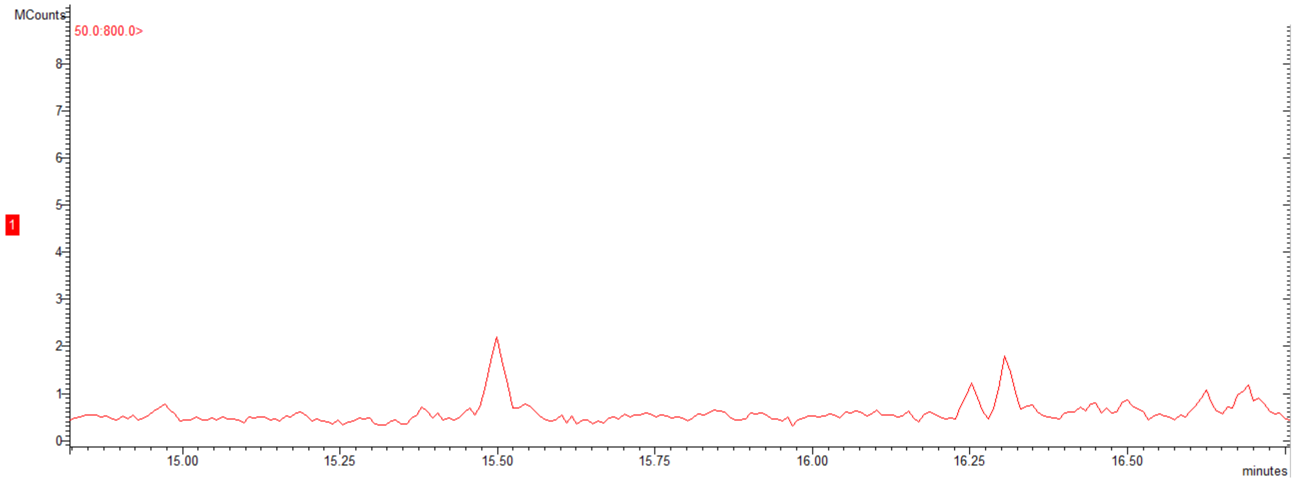

Co-injection run**.** BSRB+ 0.1 μmol γ-NH_2-butiric ac. std**.** (magnification from 14.5 min to 16.7 min).

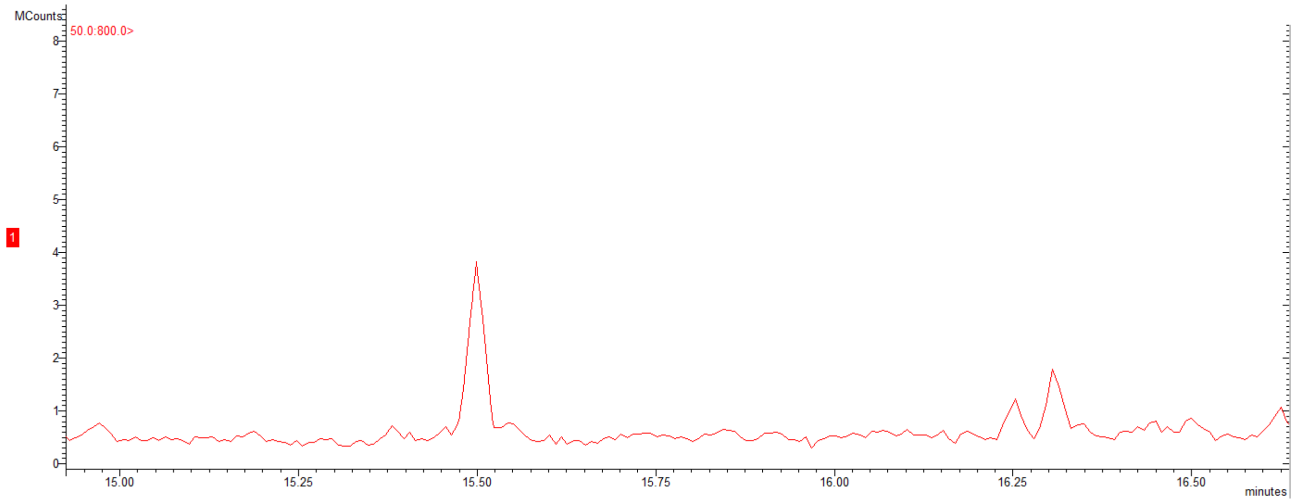

BSRB before co-injection run (magnification from 2.2 min to 2.9 min).

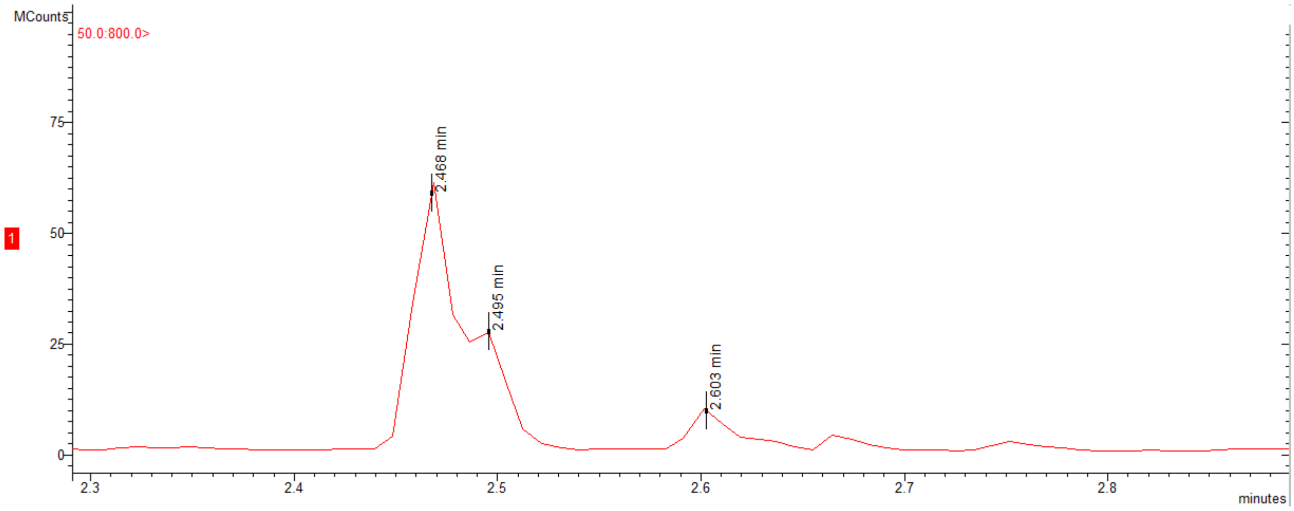

Co-injection run of BSRB+ 0.1 µmol butanamine std**.** (magnification from 2.2 min to 2.9 min).

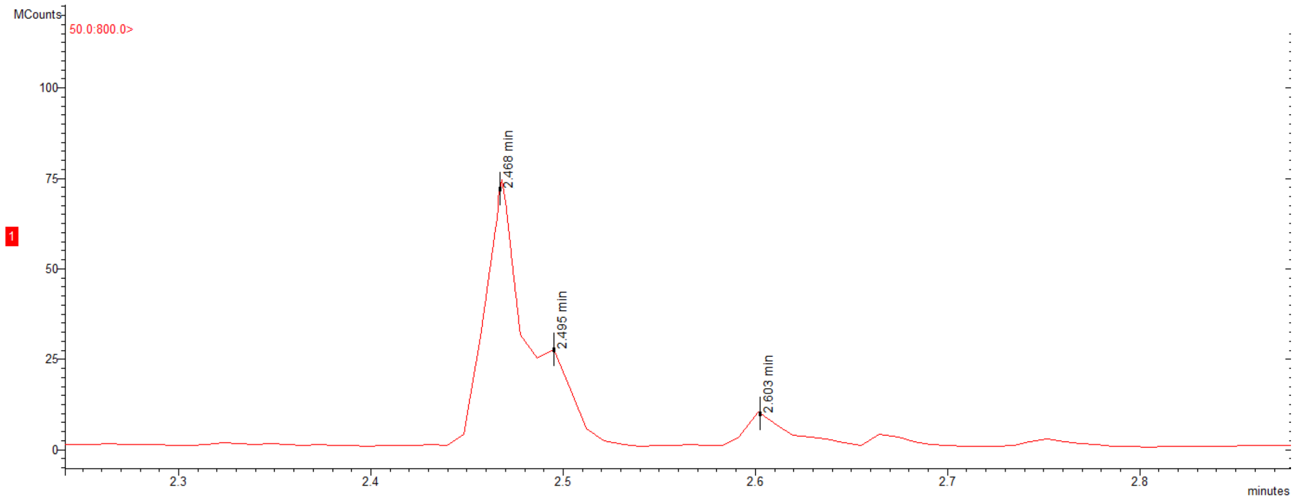

BSRB before co-injection run (magnification from 10.6 min to 11.3 min).

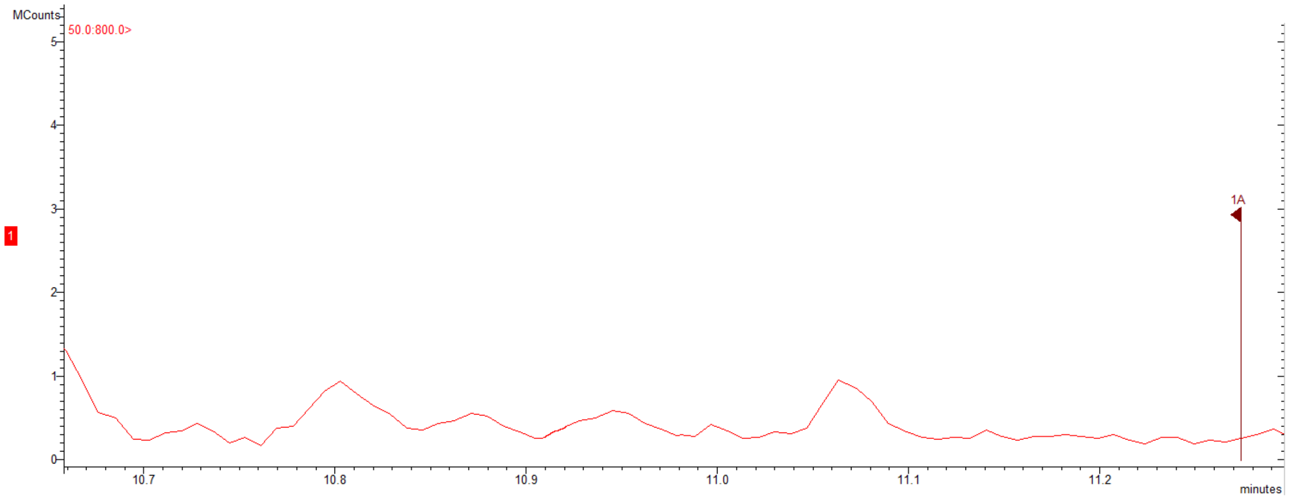

Co-injection run. BSRB+ 0.1 μmol isobutylamine std**.** (magnification from 10.6 min to 11.3 min).

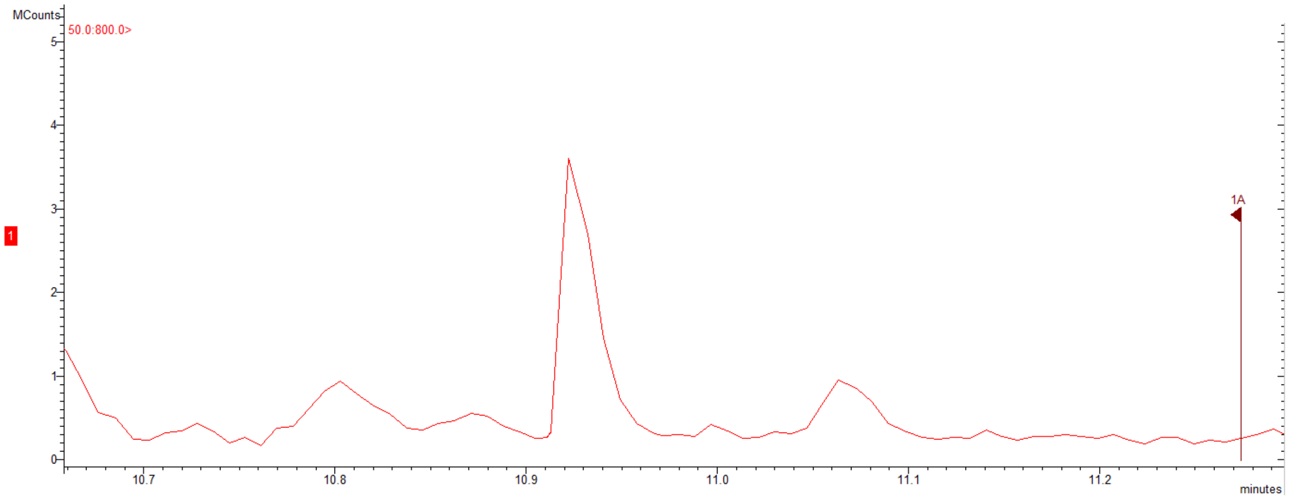

GC-Chromatogram of standard *N*-formylglycine (**20**) after derivatization in the presence of betulinic acid

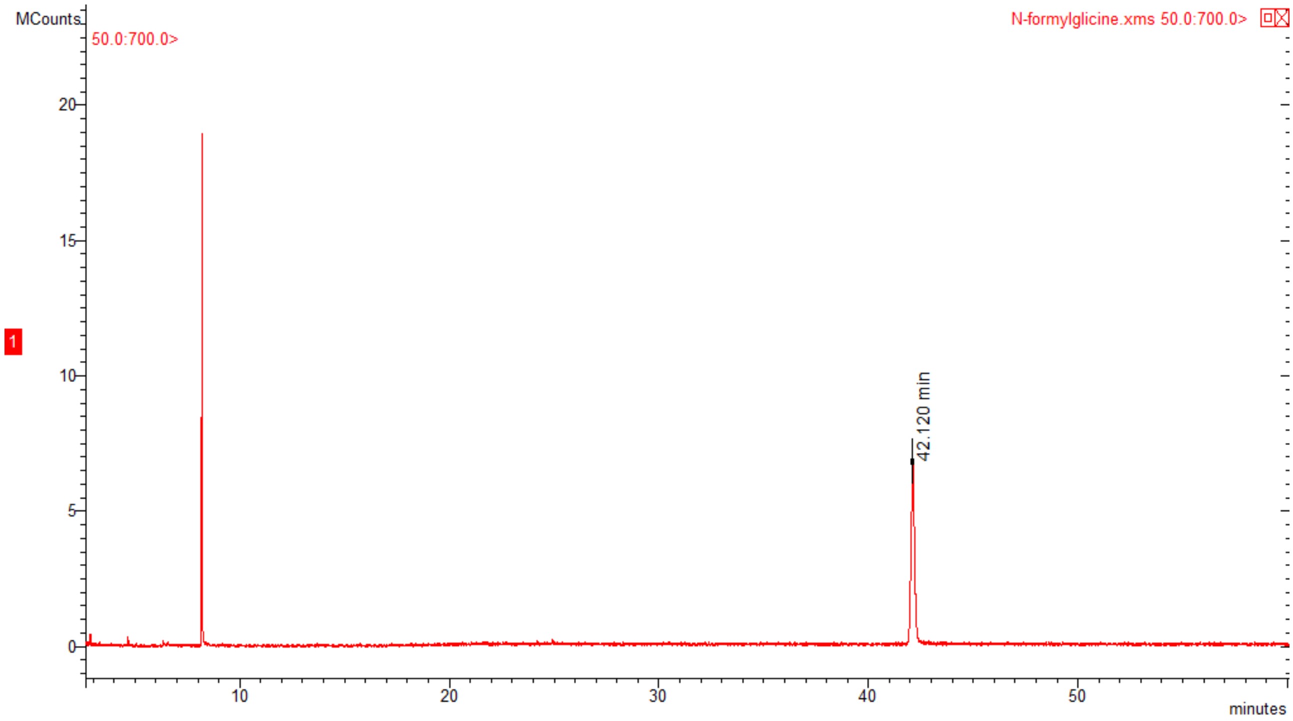

GC-Chromatogram of standard oxalic acid (**26**) after derivatization in the presence of betulinic acid

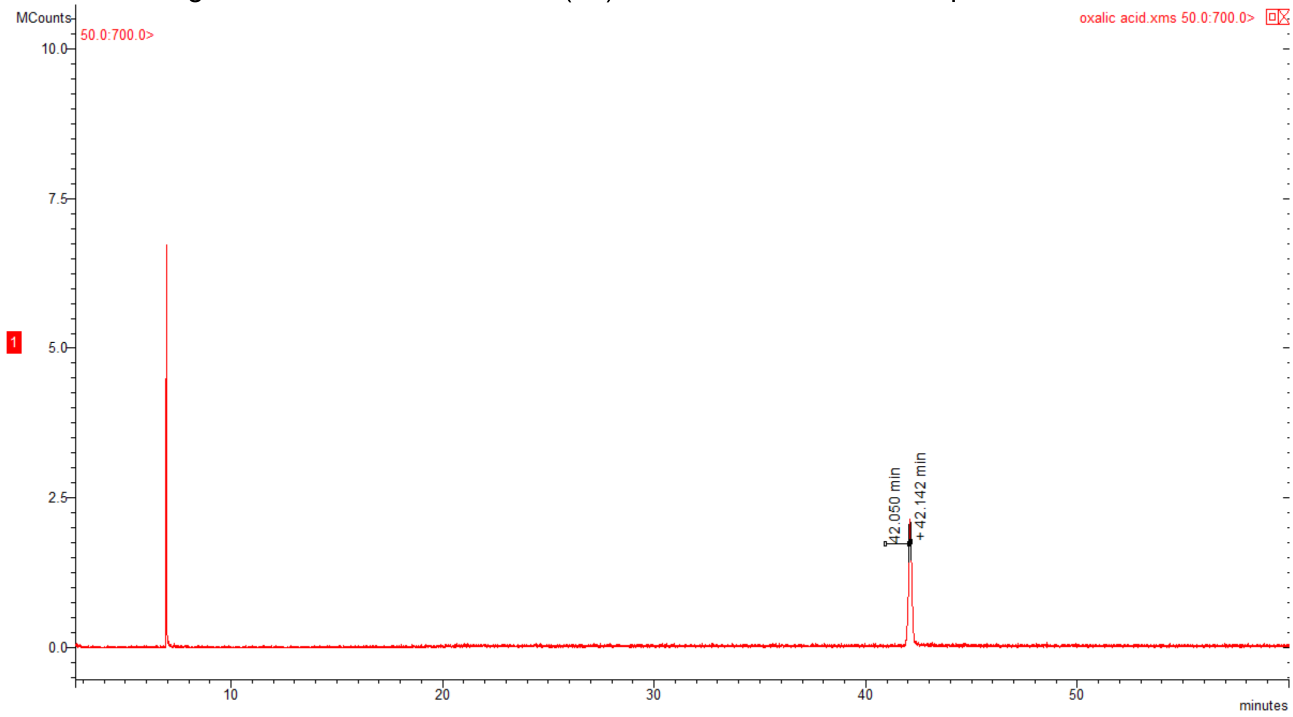

GC-Chromatogram of standard lactic acid (**28**) after derivatization in the presence of betulinic acid

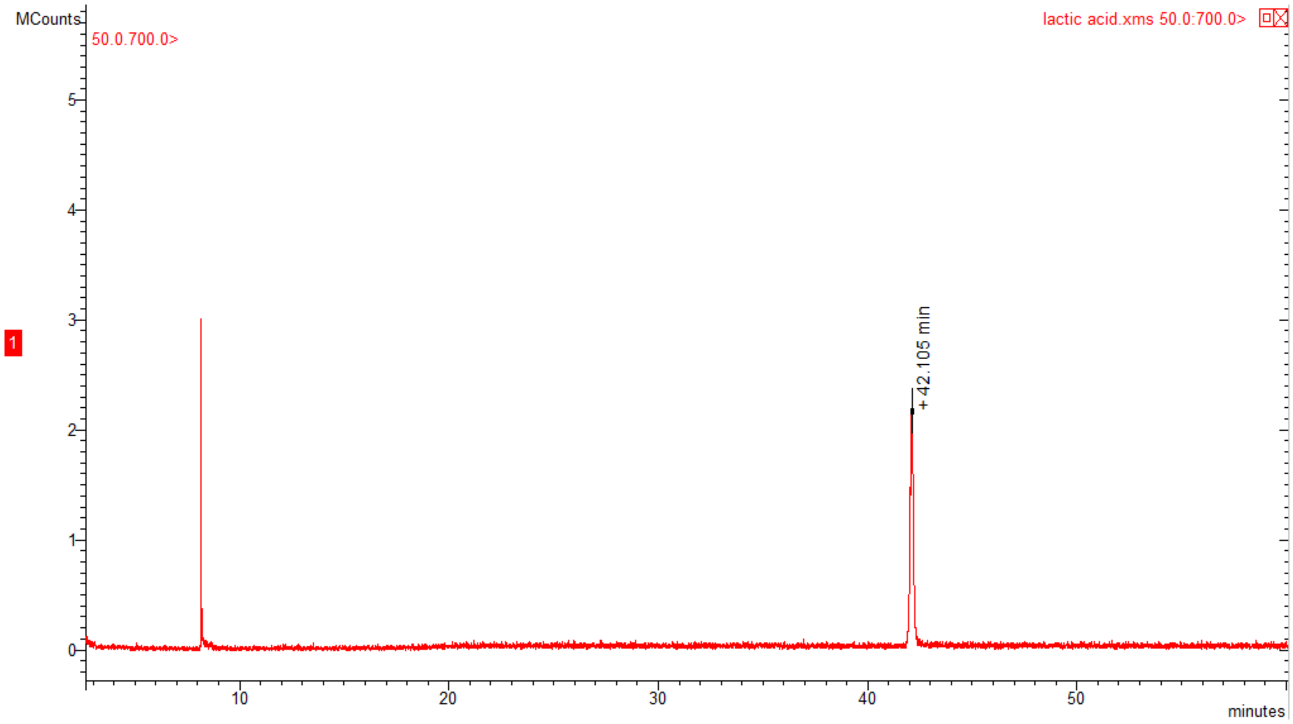

GC-Chromatogram of standard guanine (**37**) after derivatization in the presence of betulinic acid

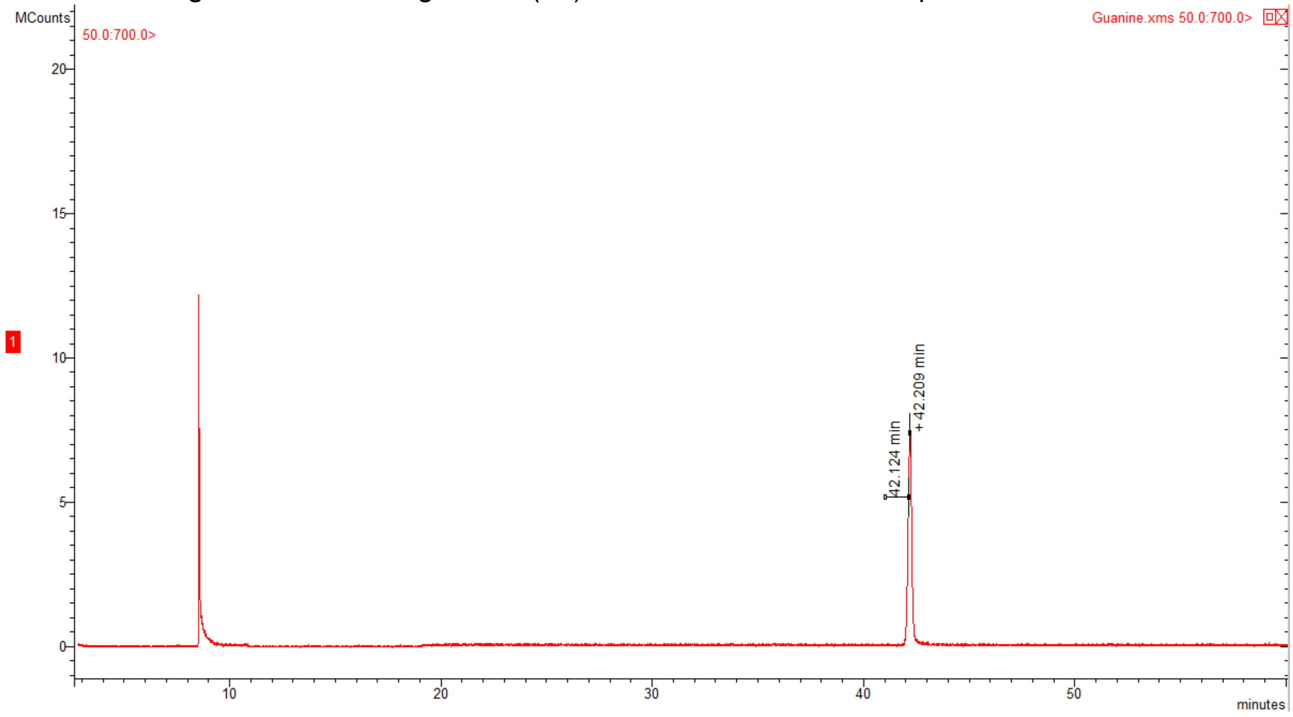

GC-Chromatogram of standard uracil (**38**) after derivatization in the presence of betulinic acid

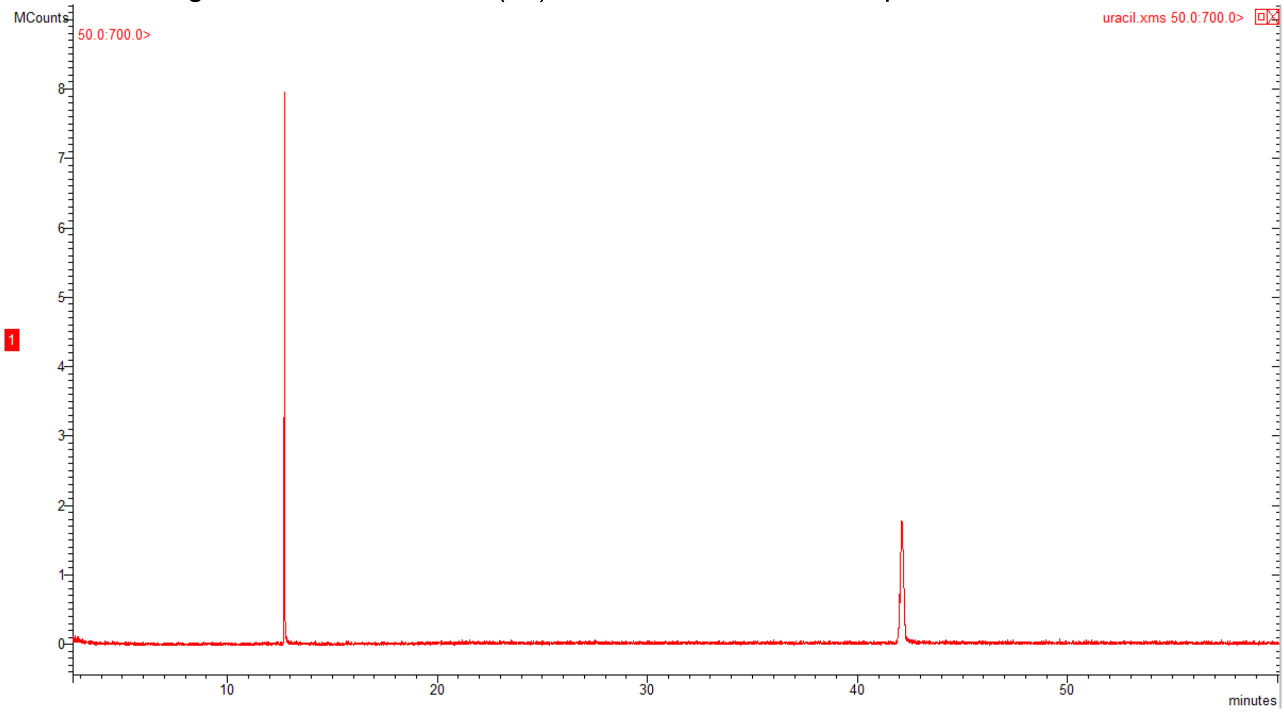

GC-Chromatogram of standard hydroxy-naphthalene (**45**) after derivatization in the presence of betulinic acid

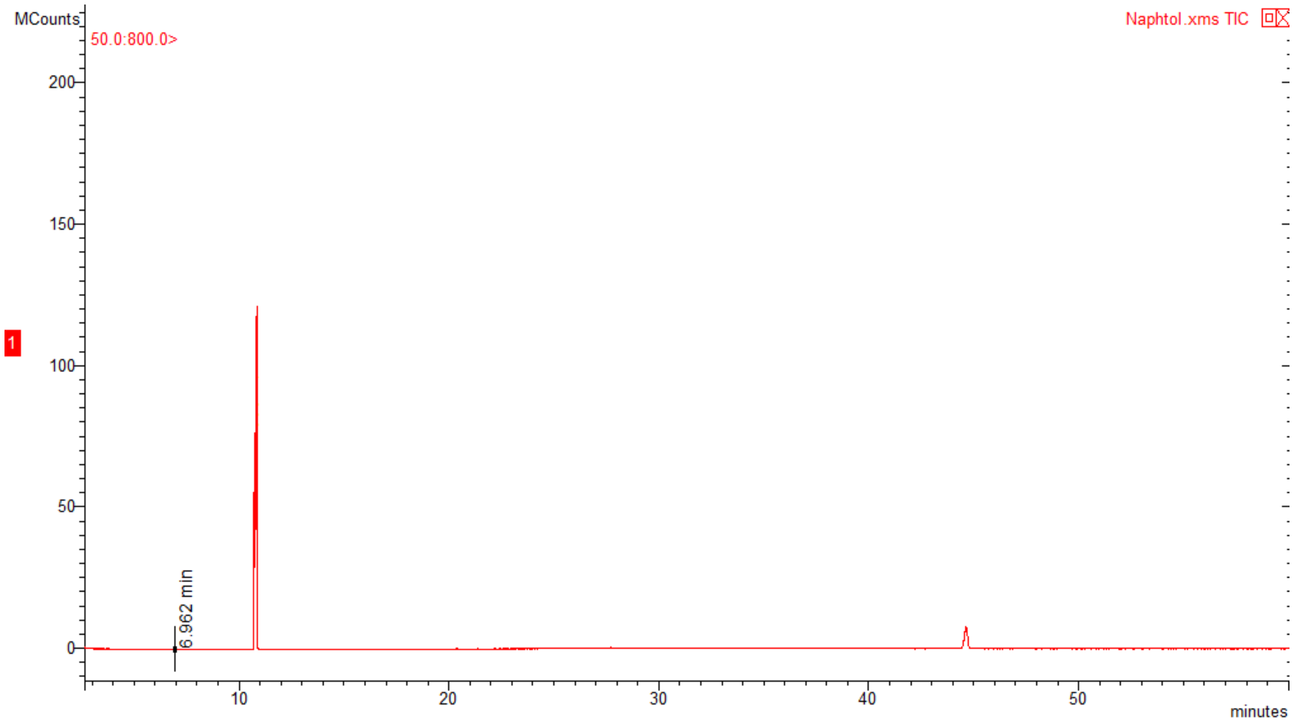

SI #4 Original m/z fragmentation spectra of compounds (1-48) and selected spectra of standards.

Formamide[(b)] (**1**)

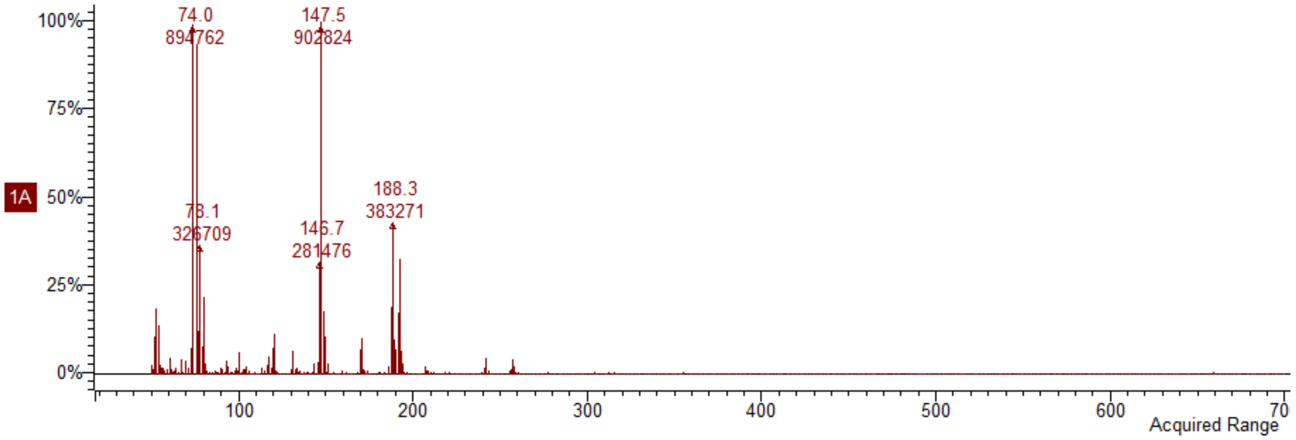

Formic acid[(a)] (**2**)

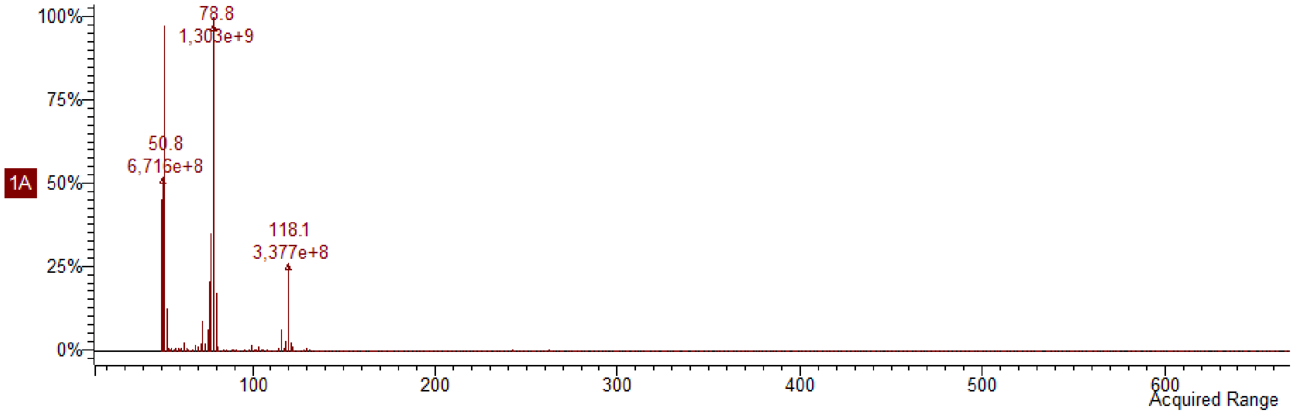

Urea[b] (**3**)

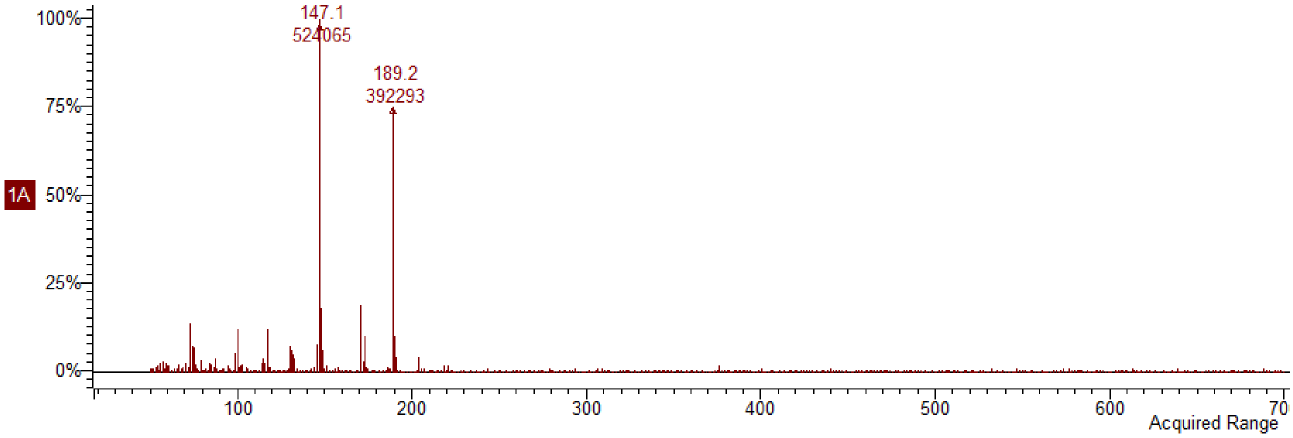

DAMN[a] (**4**)

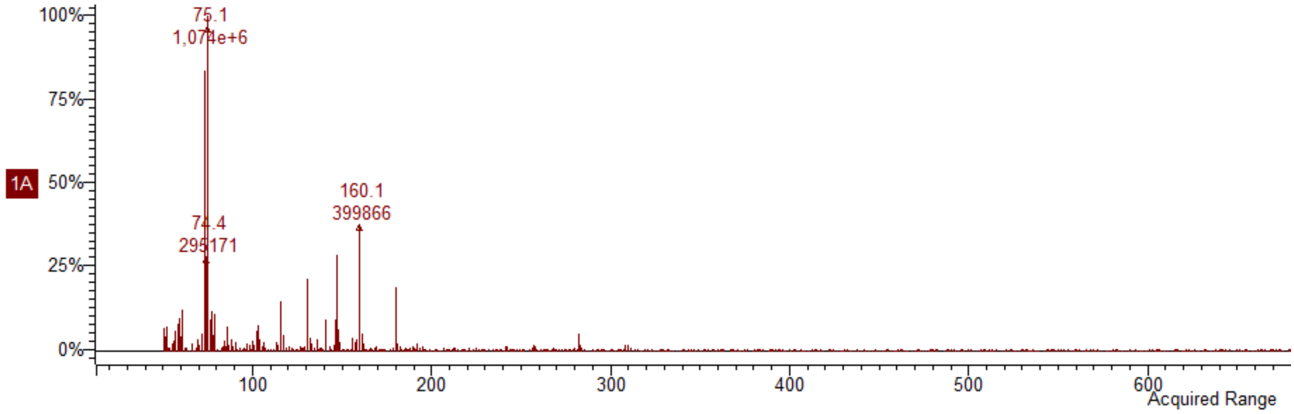

Glycine[a] (**5**)

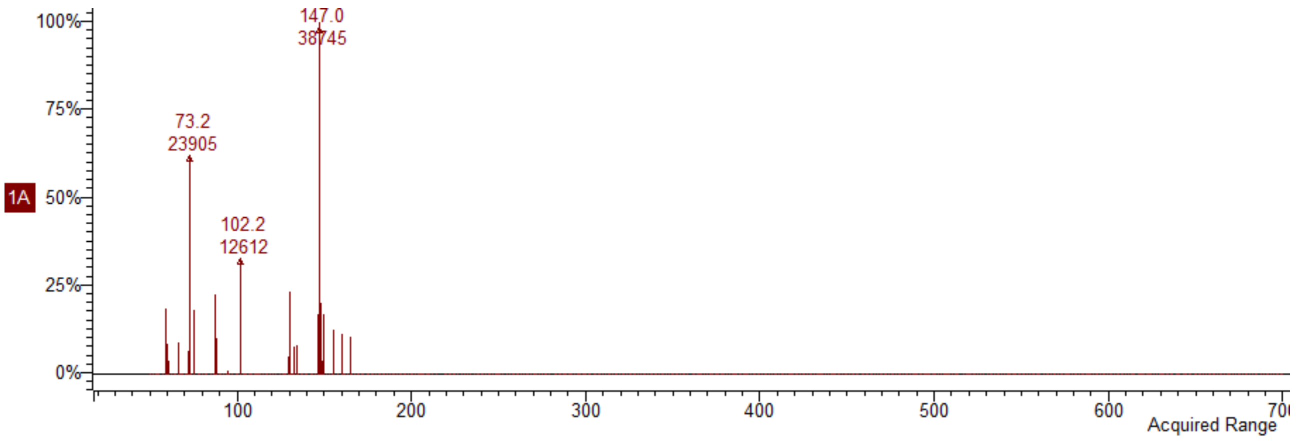

Glycine(b) (**5**)

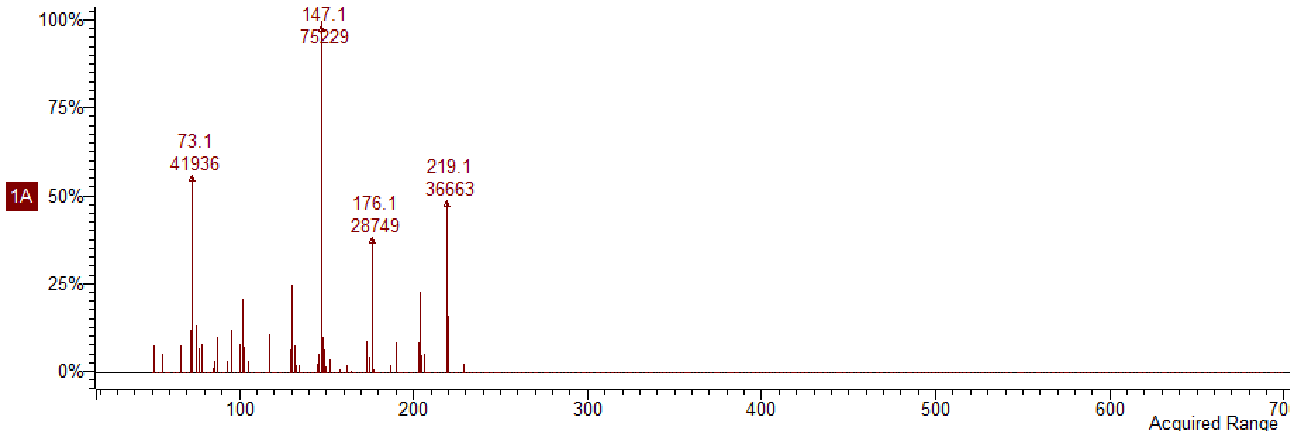

Alanine(b) (**6**)

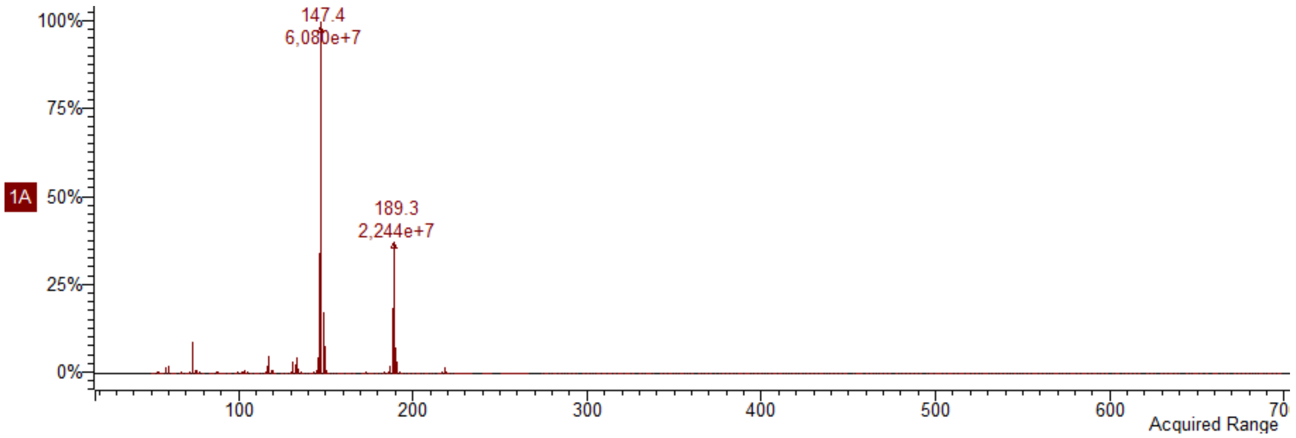

Valine[(b)] (**7**)

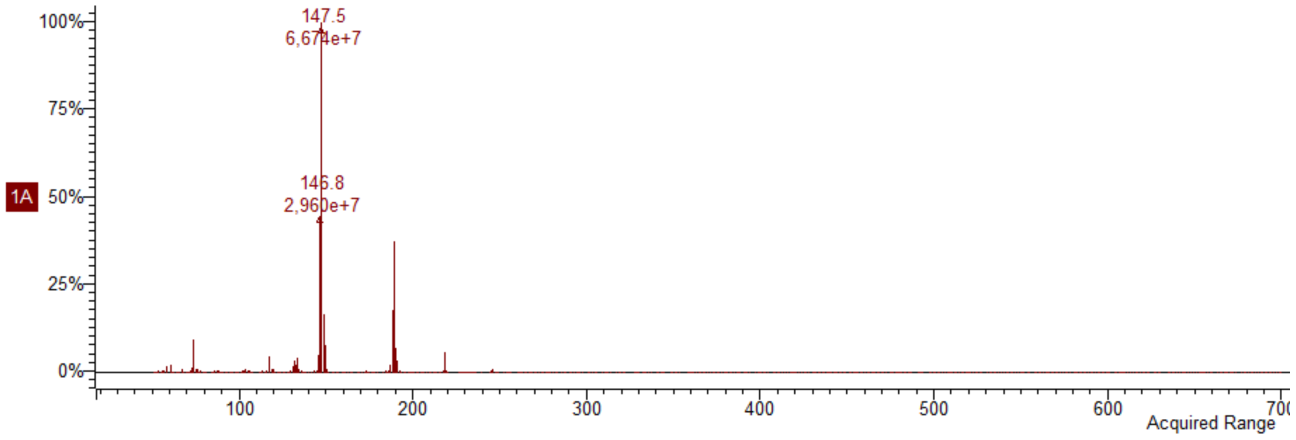

Leucine[(b)] (**8**)

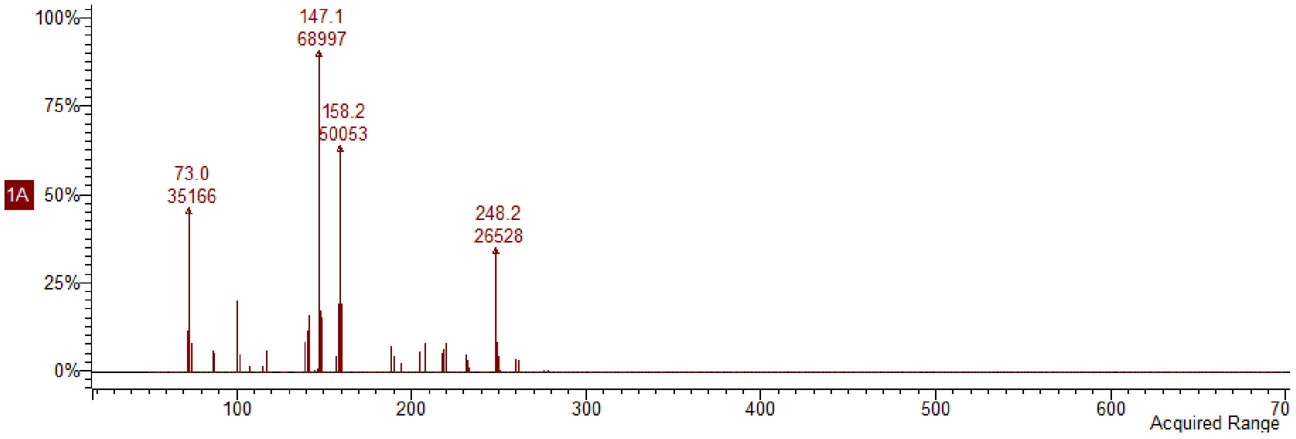

Proline[(b)] (**9**)

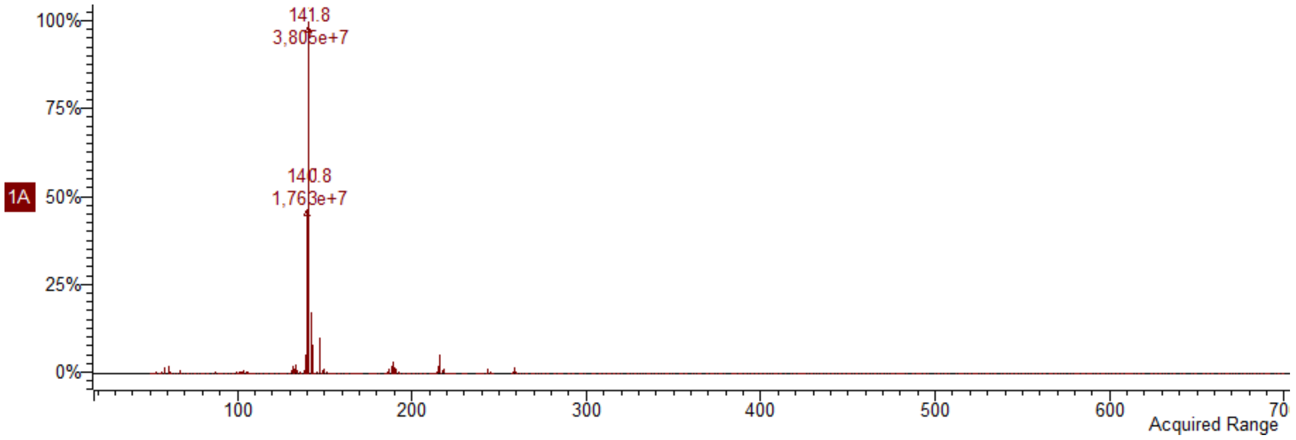

Serine[(c)] (**10**)

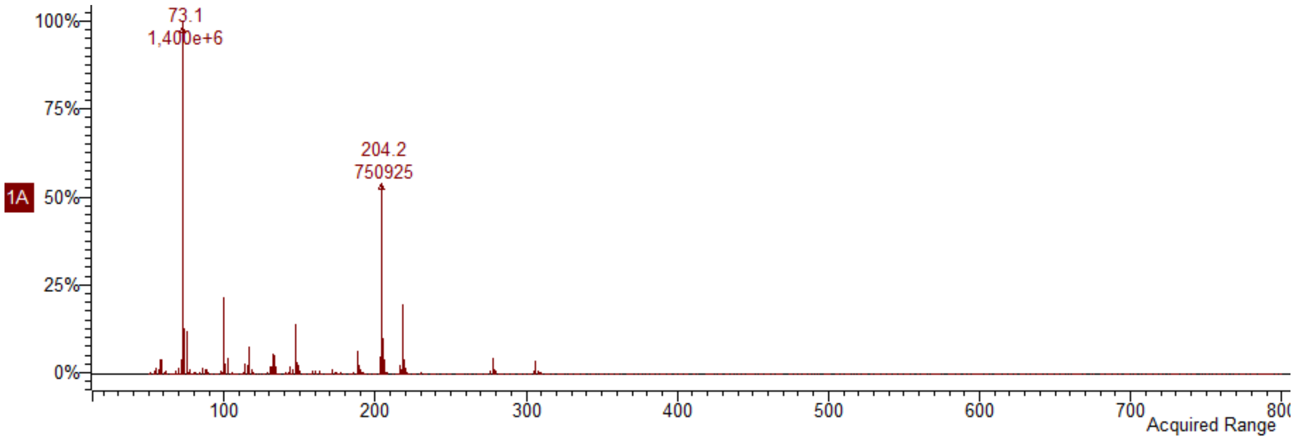

Asparagine[c] (**11**)

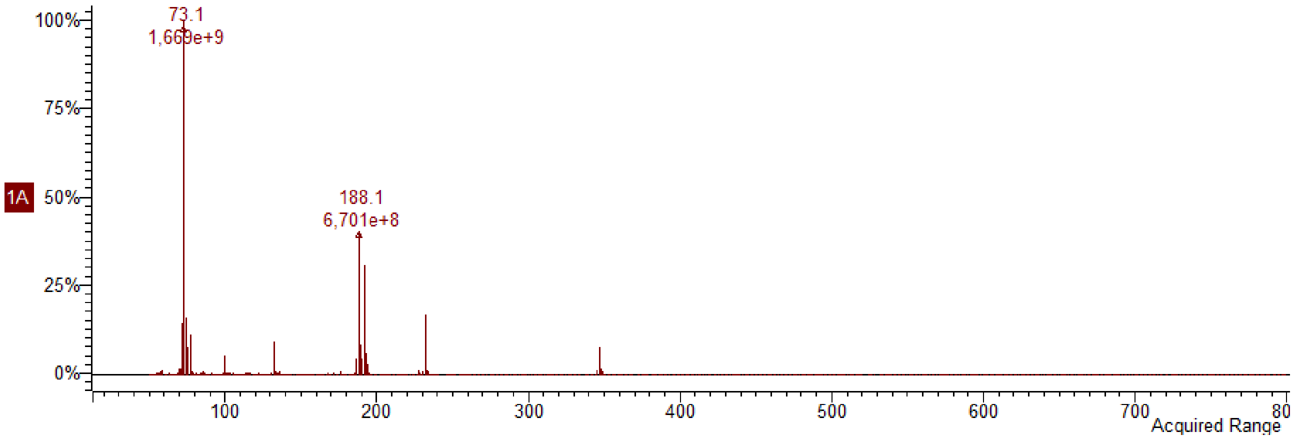

Aspartic ac.[c] (**12**)

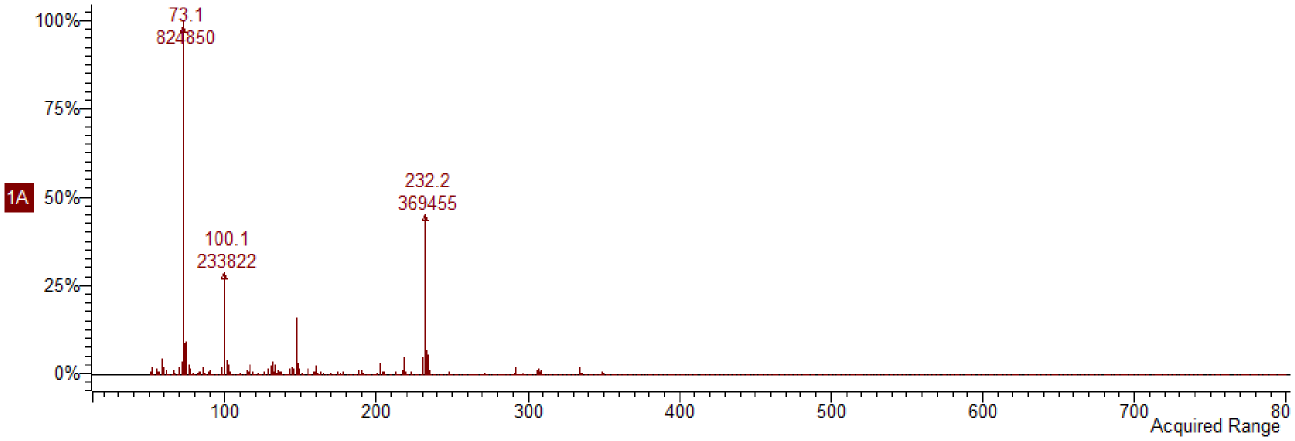

Glutamic ac.[c] (**13**)

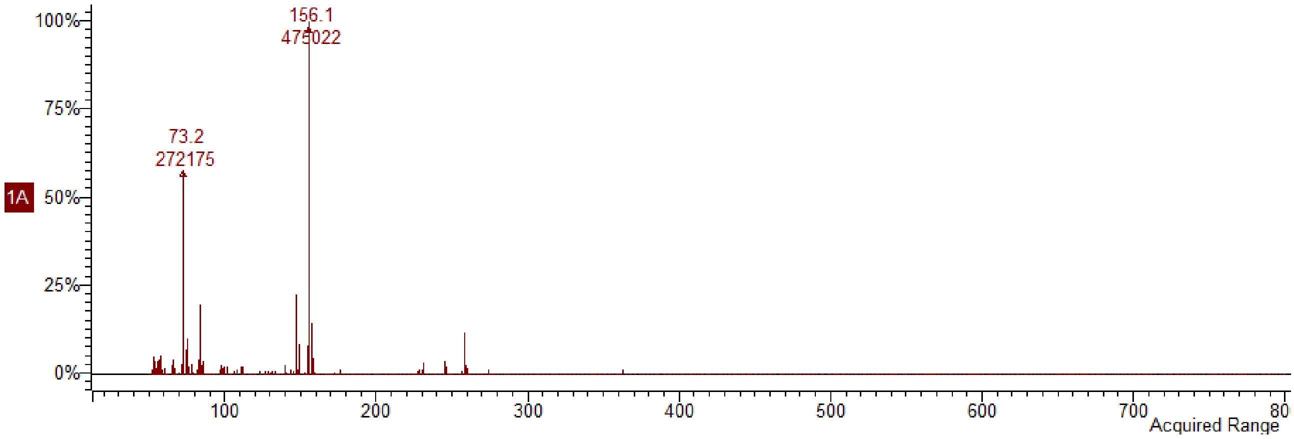

Lysine[d] (**14**)

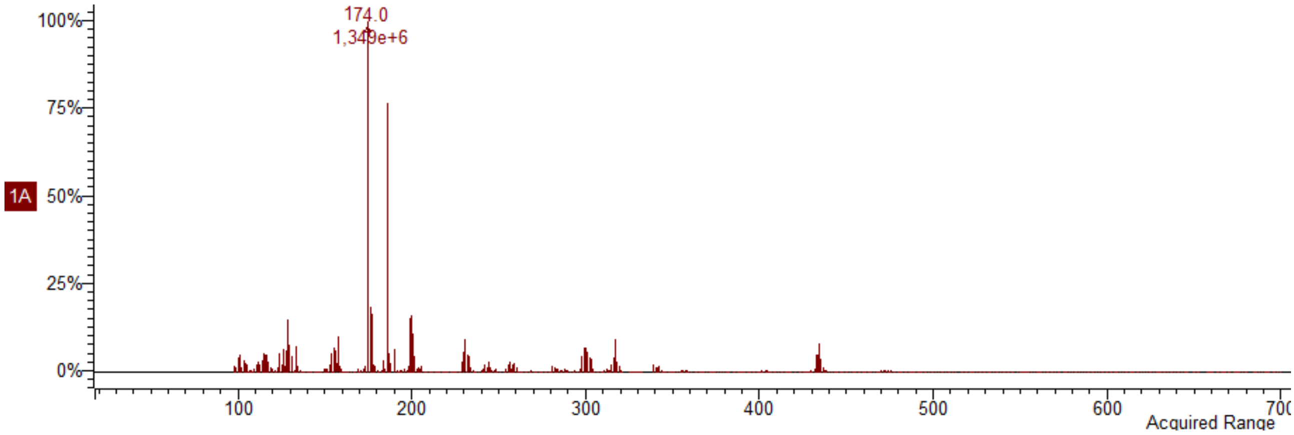

Histidine[b] (**15**)

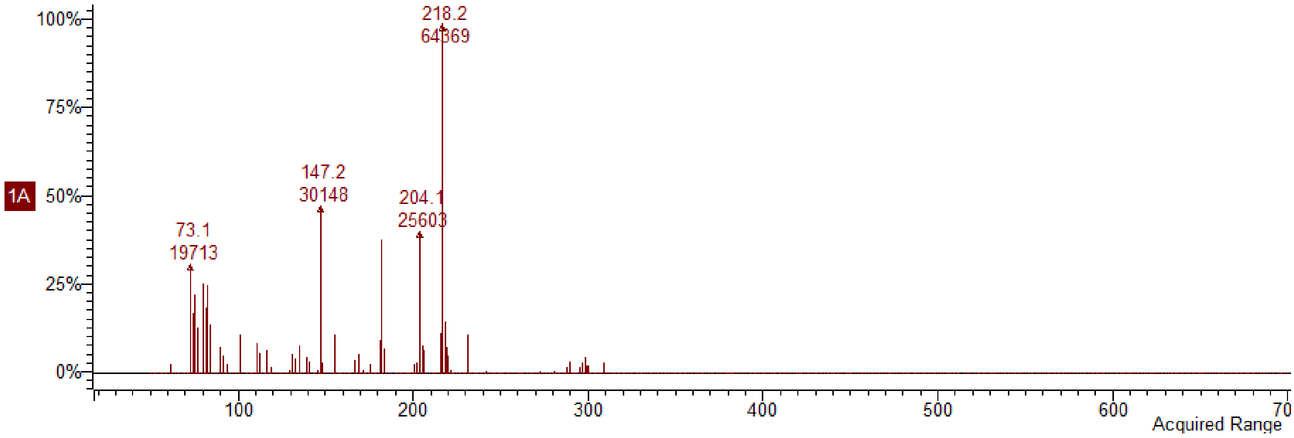

β-Alanine[b] (**16**)

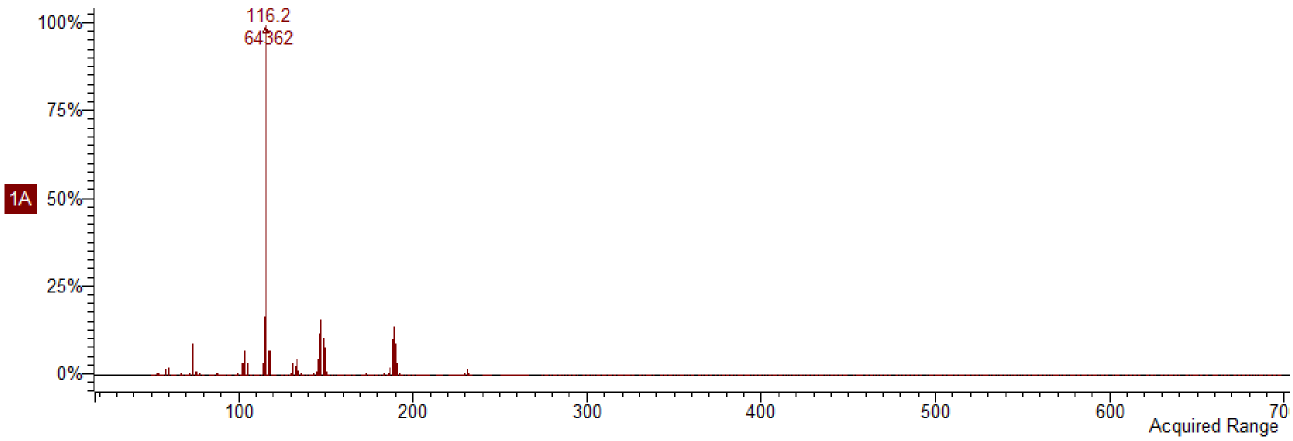

Isovaline[(b)] (**17**)

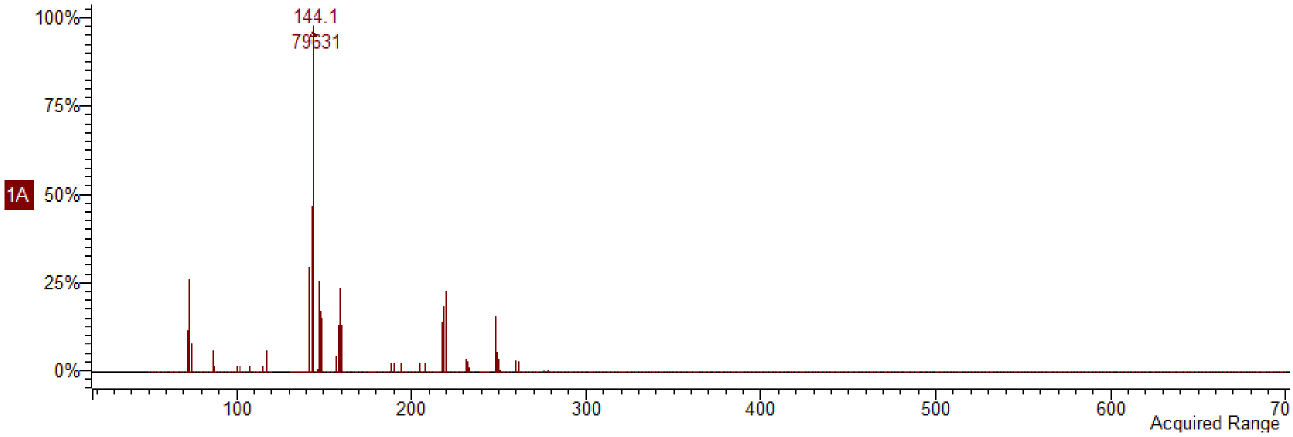

α-NH_2-isobutyric ac.[(b)] (**18**)

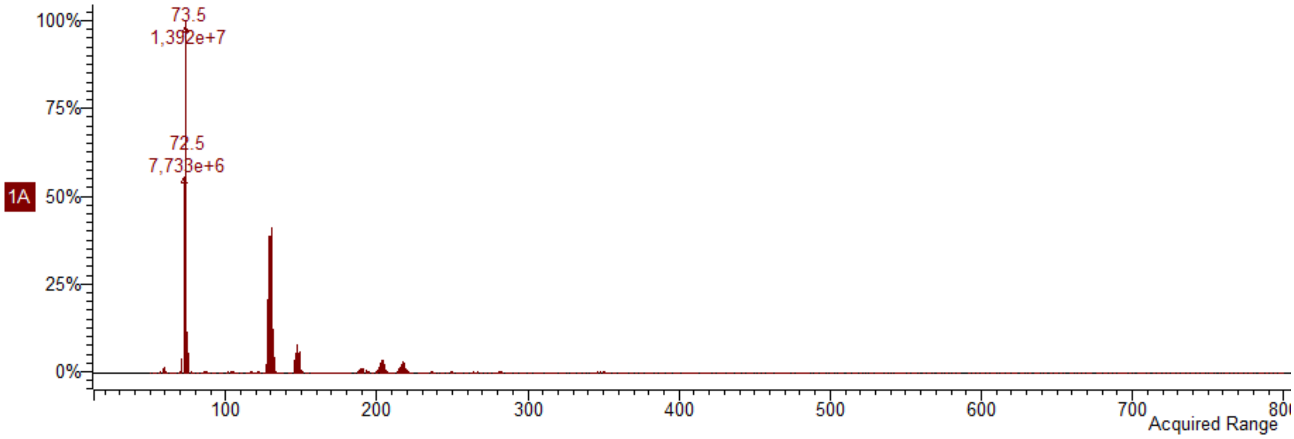

γ-NH_2-butyric ac.[(c)] (**19**)

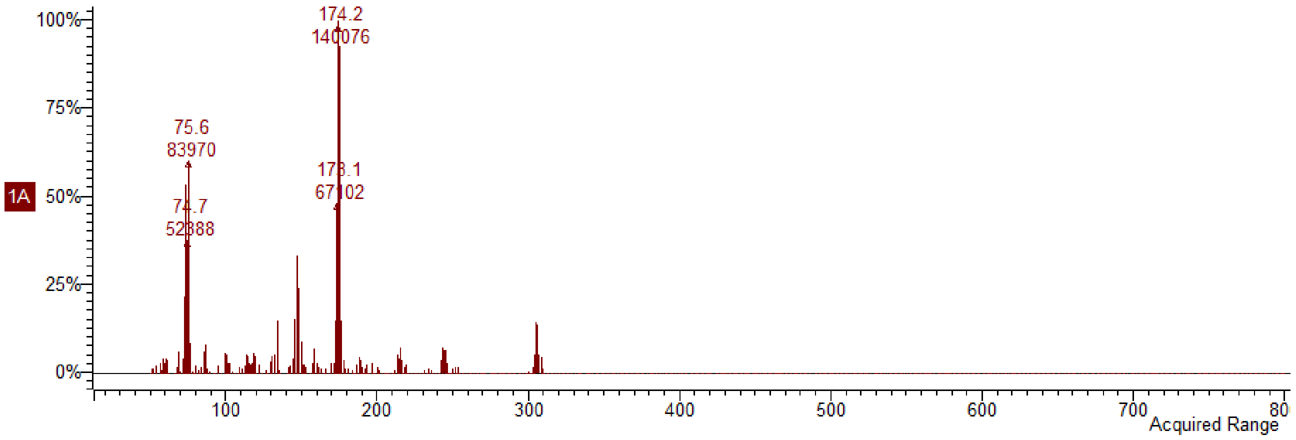

N-fGlycine [(b)] (**20**)

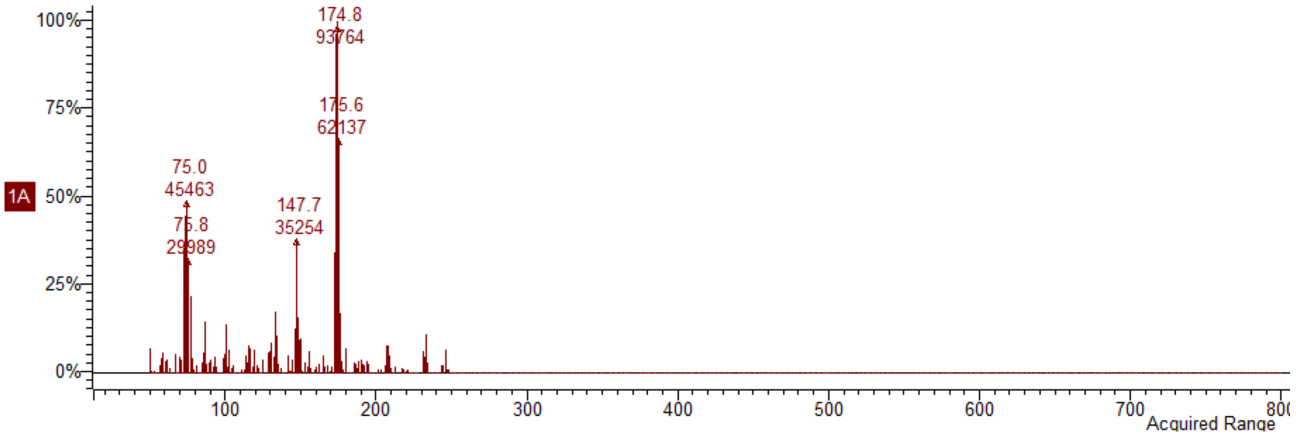

N-fLeucine [(a)] (**21**)

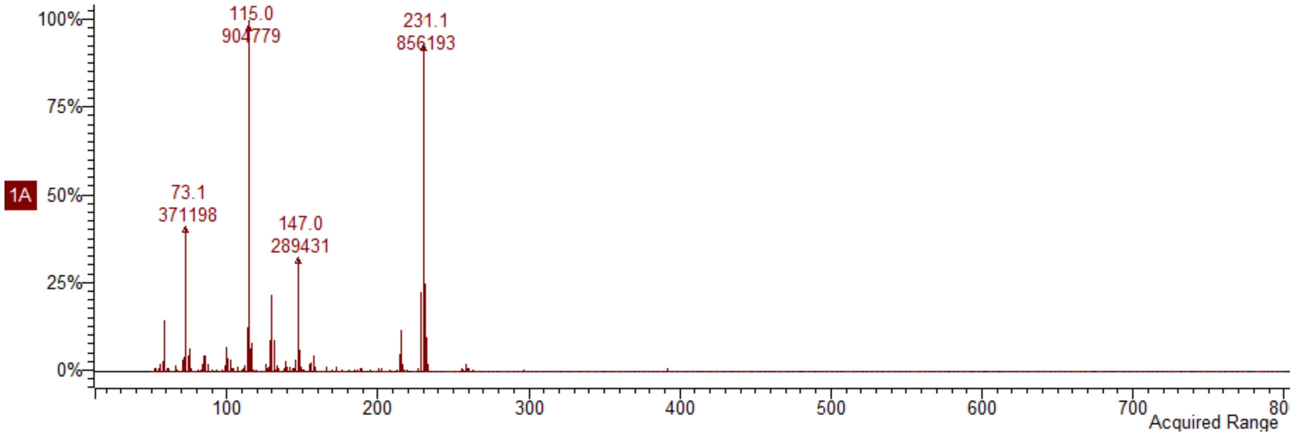

Glycylglycine[(a)] (**22**)

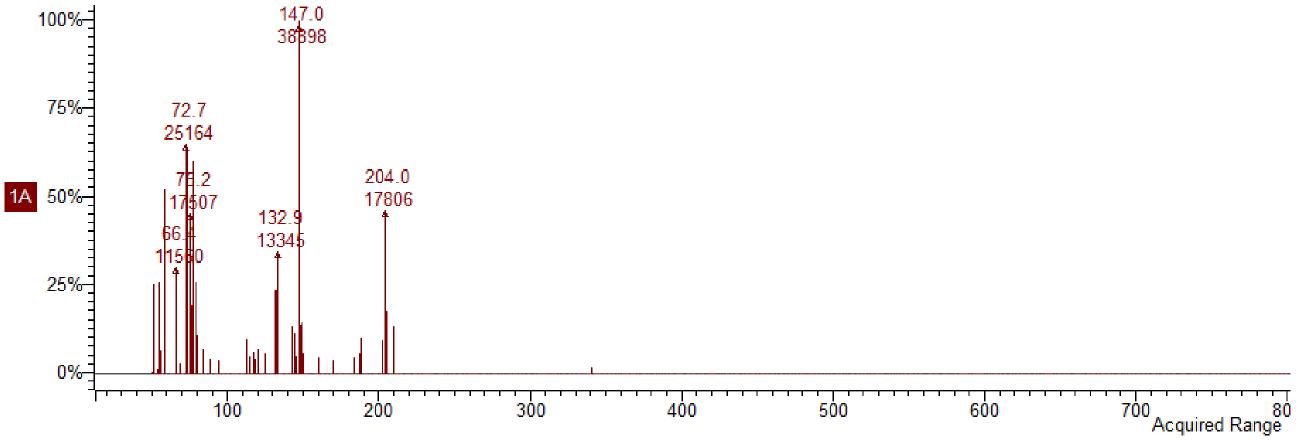

1-Butanamine (**23**)

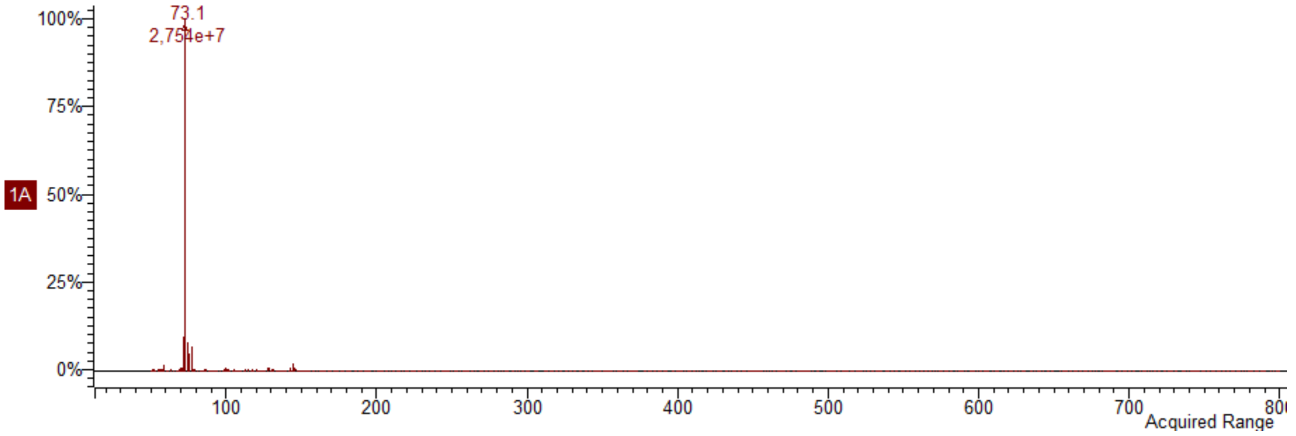

Isobutylamine (**24**)

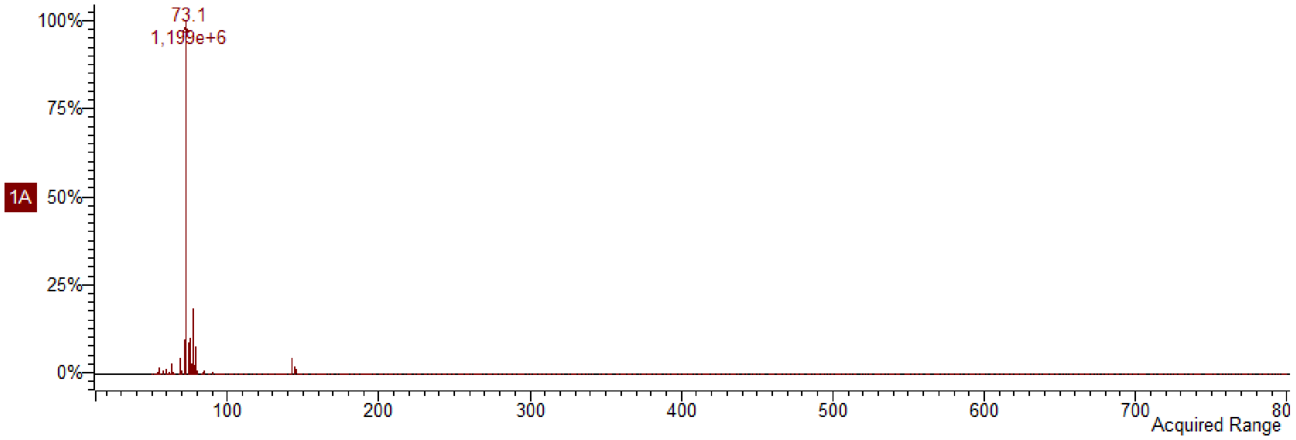

Glycolic ac.(b) (**25**)

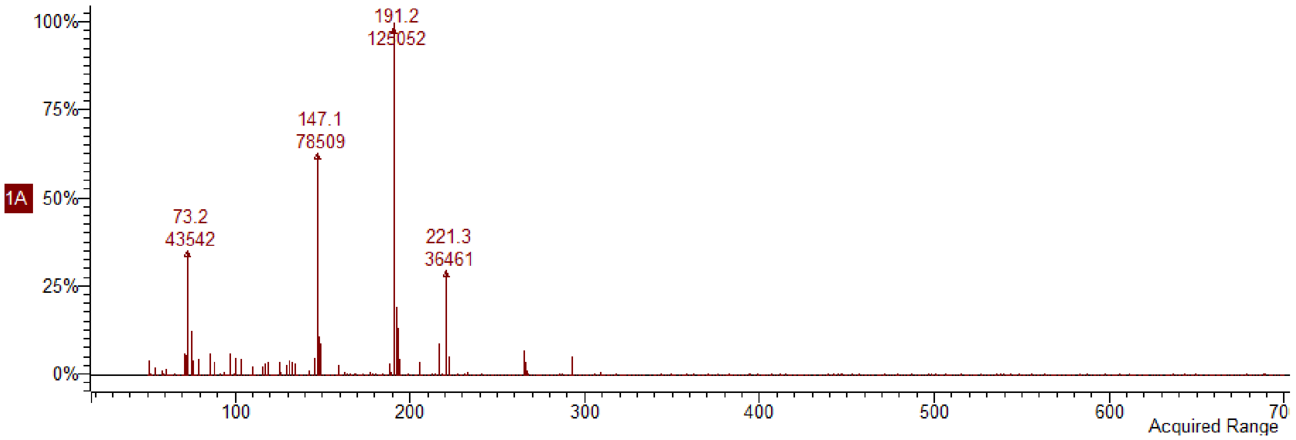

Oxalic ac.(b) (**26**)

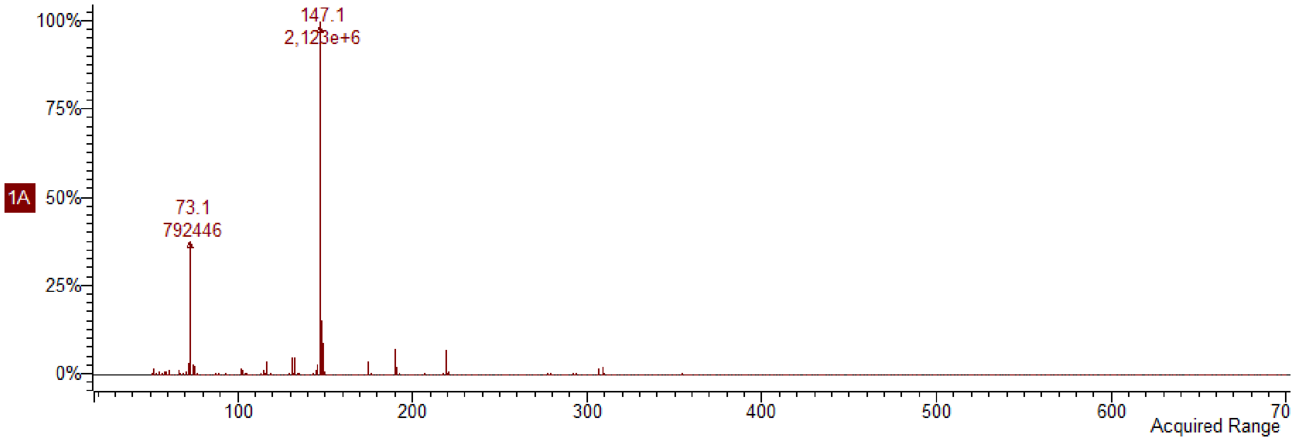

Pyruvic ac.(b) (**27**)

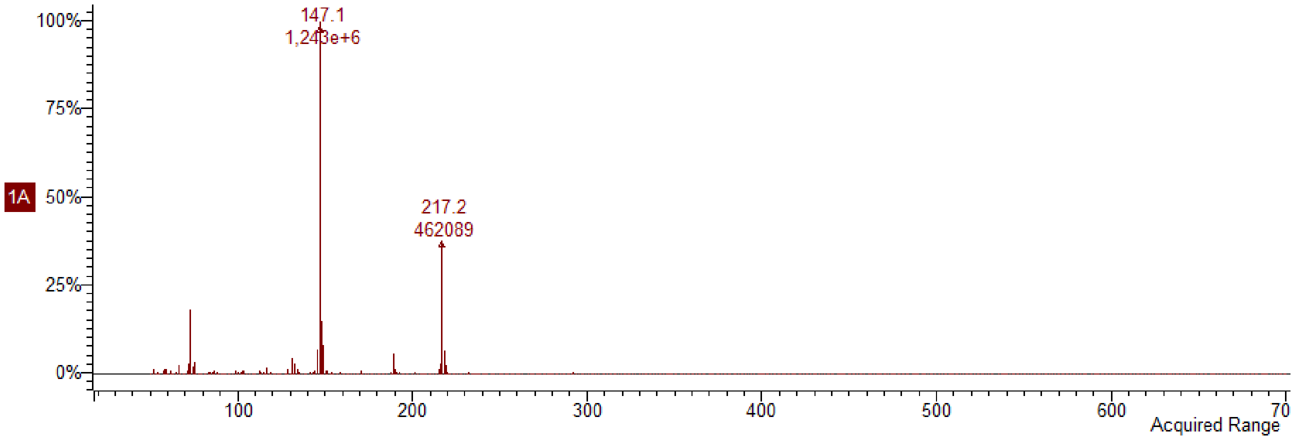

Lactic ac.[c] (**28**)

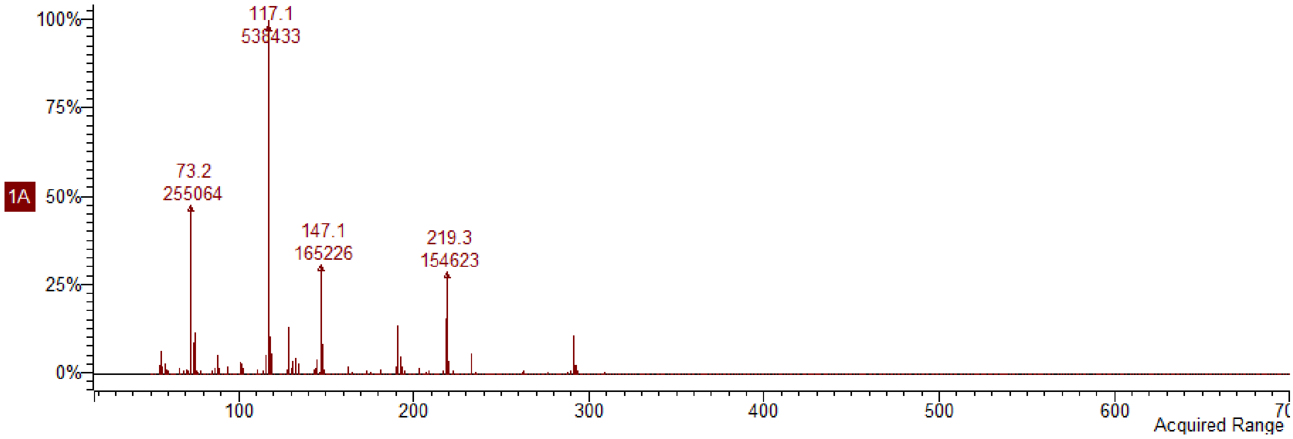

Maleic ac.[b] (**29**)

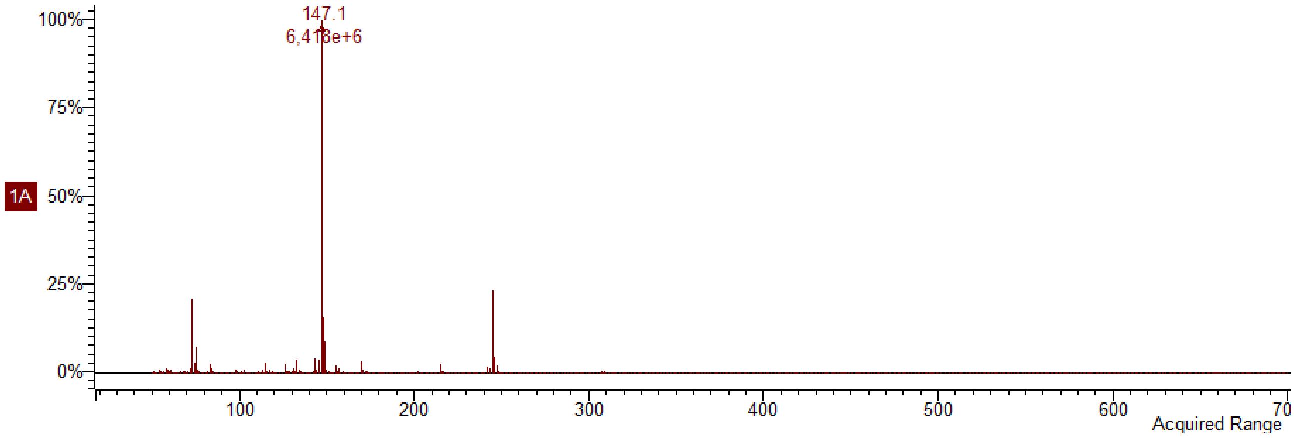

Malic ac.[c] (**30**)

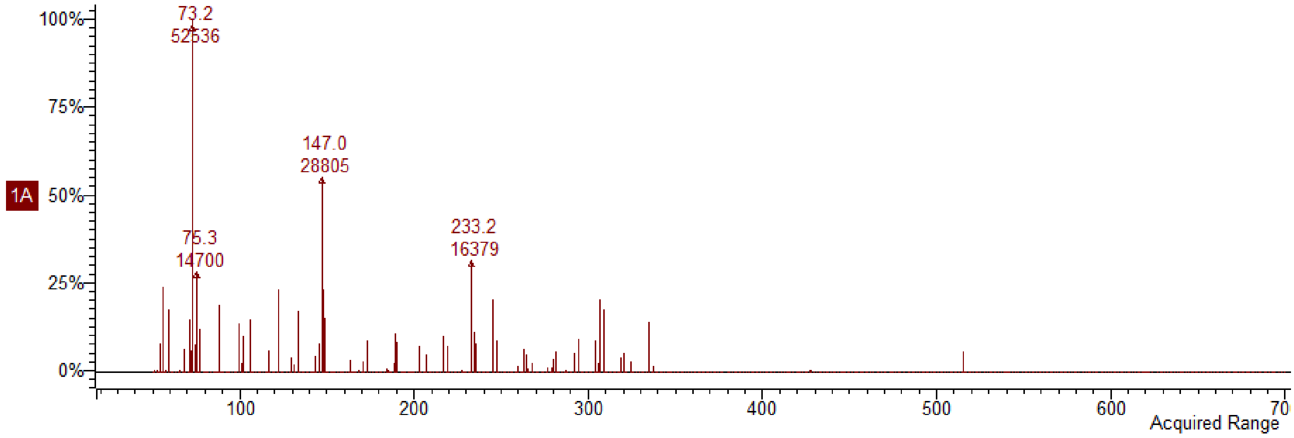

Oxaloacetic ac.[c] (**31**)

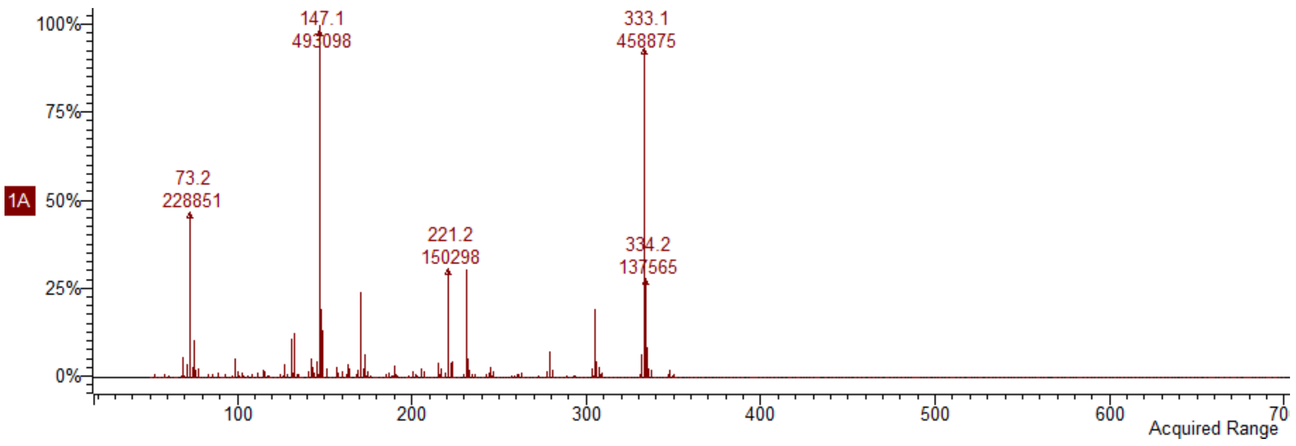

2-Ketoglutaric ac.[c] (**32**)

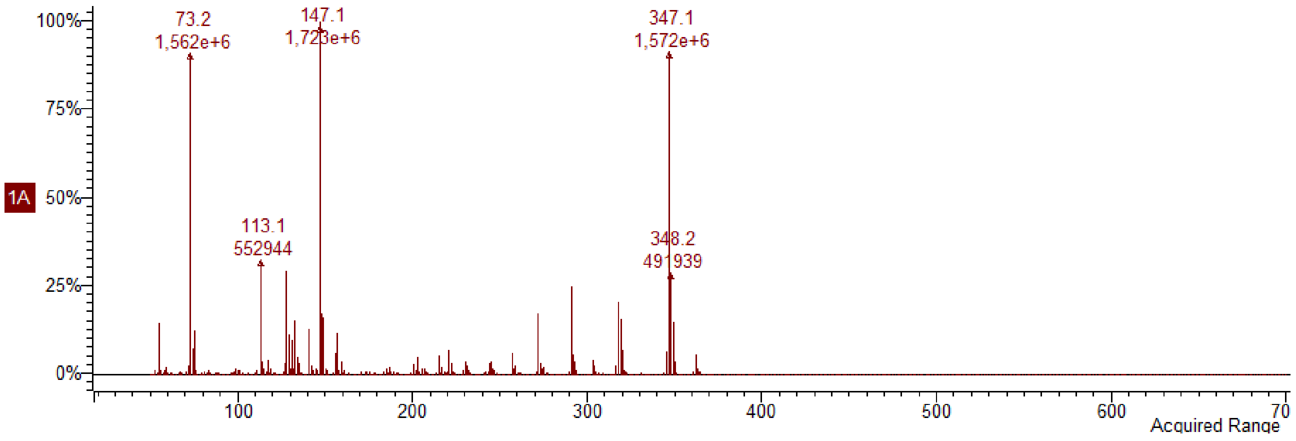

Hexanoic ac.[a] (**33**)

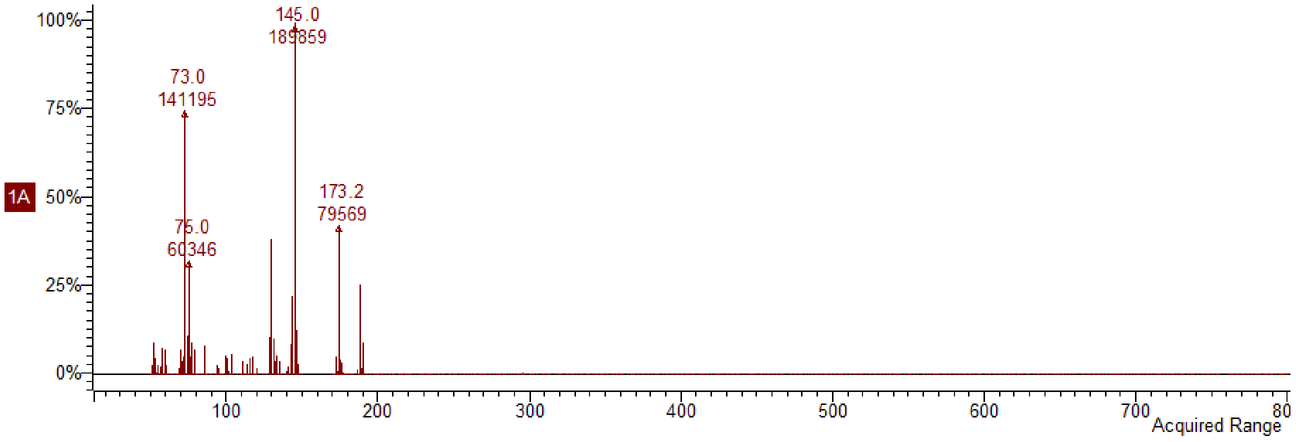

Nonanoic ac.[(a)] (**34**)

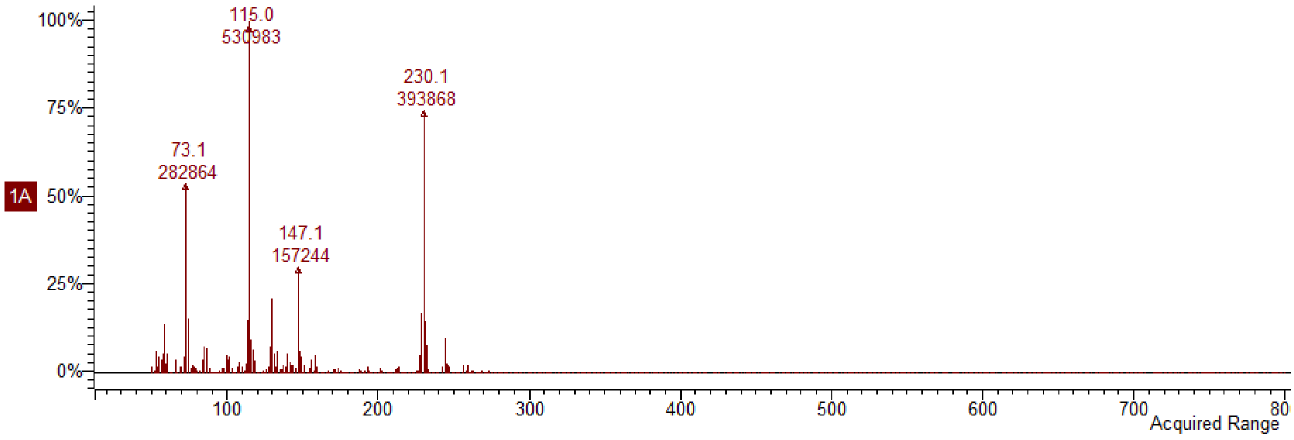

Gentisic ac.[(c)] (**35**)

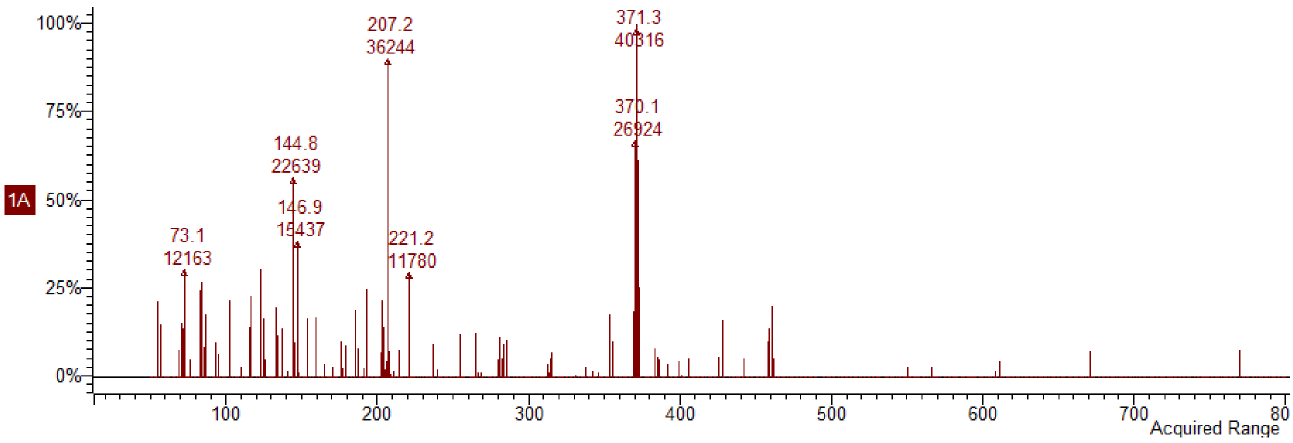

Adenine[(a)] (**36**)

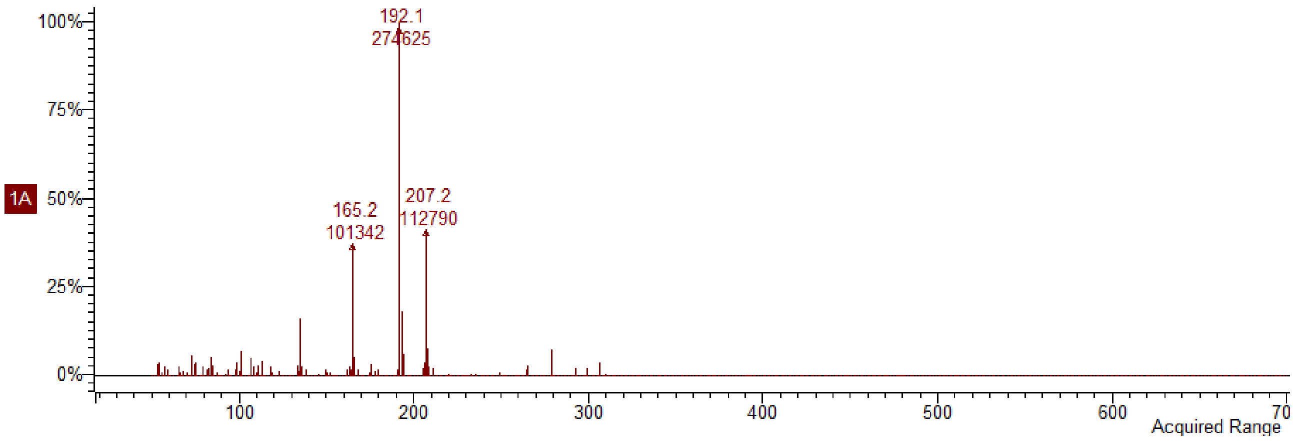

Guanine[c] (**37**)

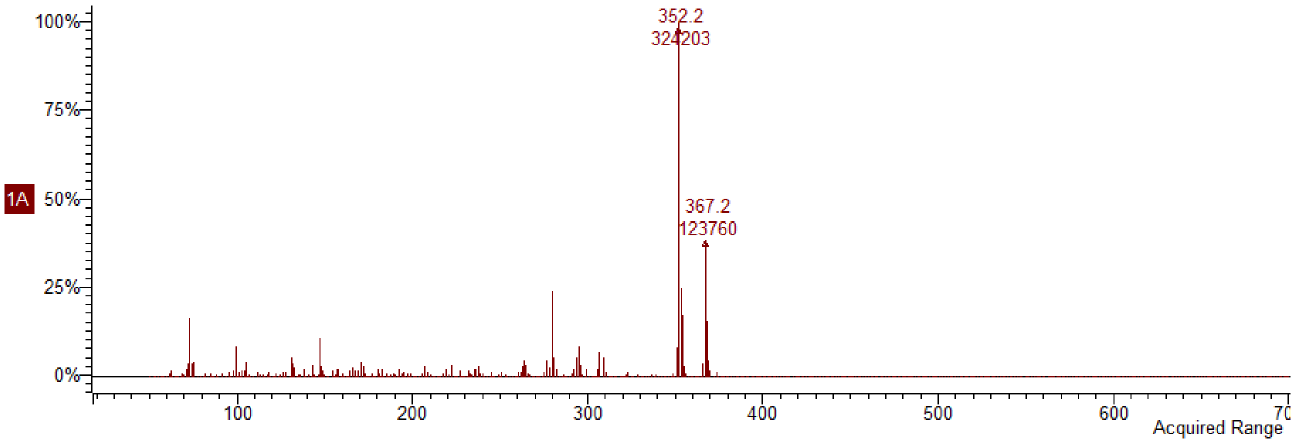

Uracil[b] (**38**)

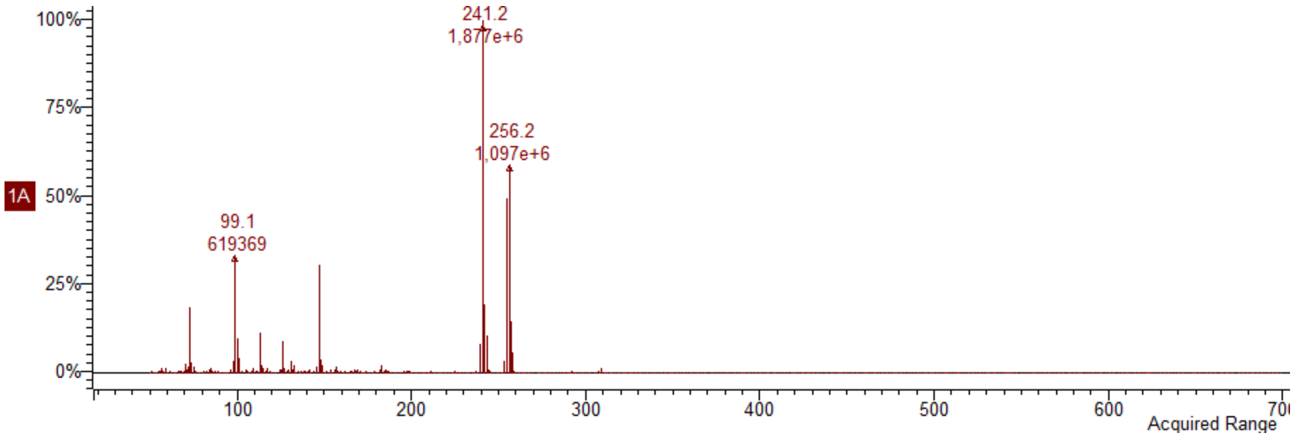

Cytosine[a] (**39**)

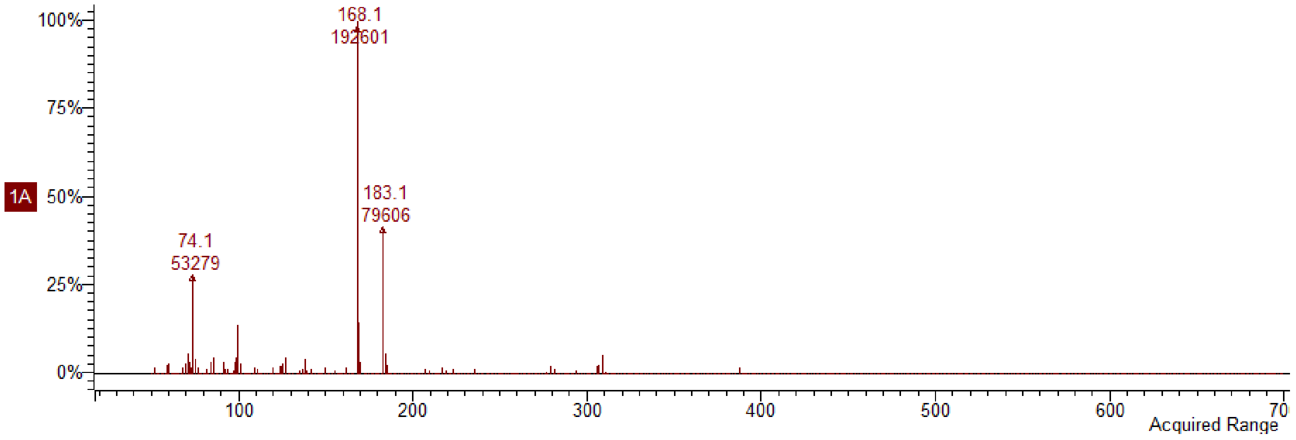

Thymine[b] (**40**)

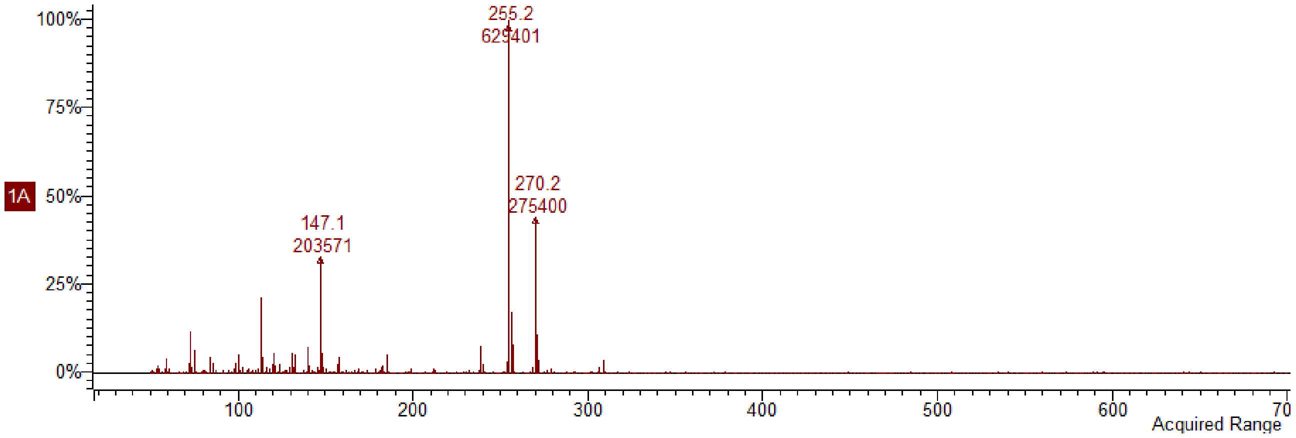

Parabanic ac.(b) (**41**)

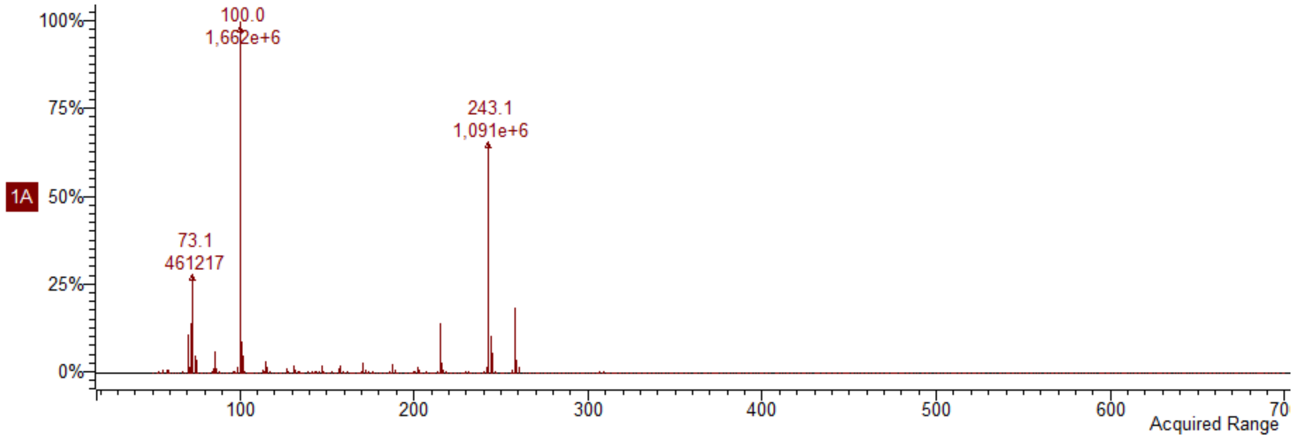

3,5-diNH$_2$-1,2,4-triazole(c) (**42**)

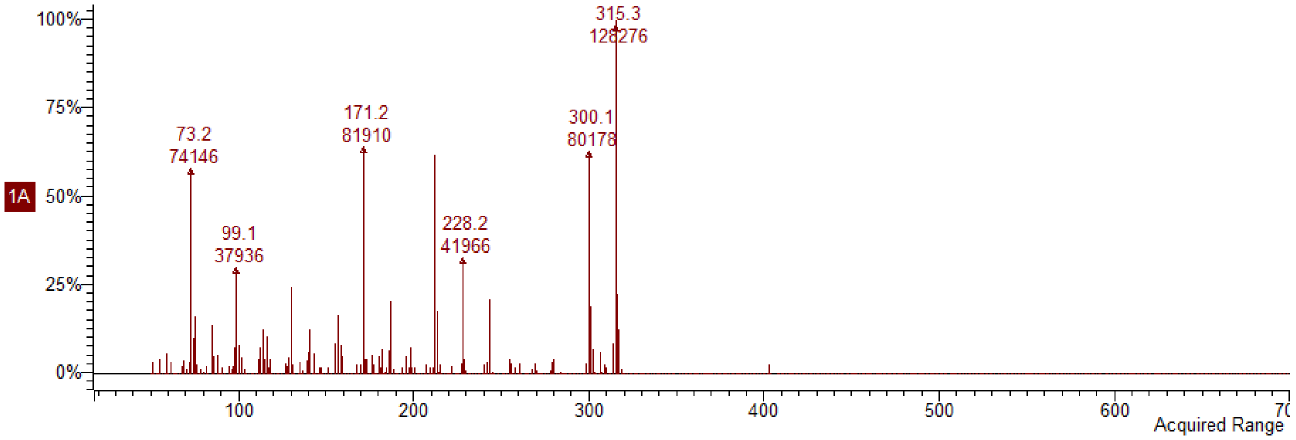

1H-Indole-3-methanamine (**43**)

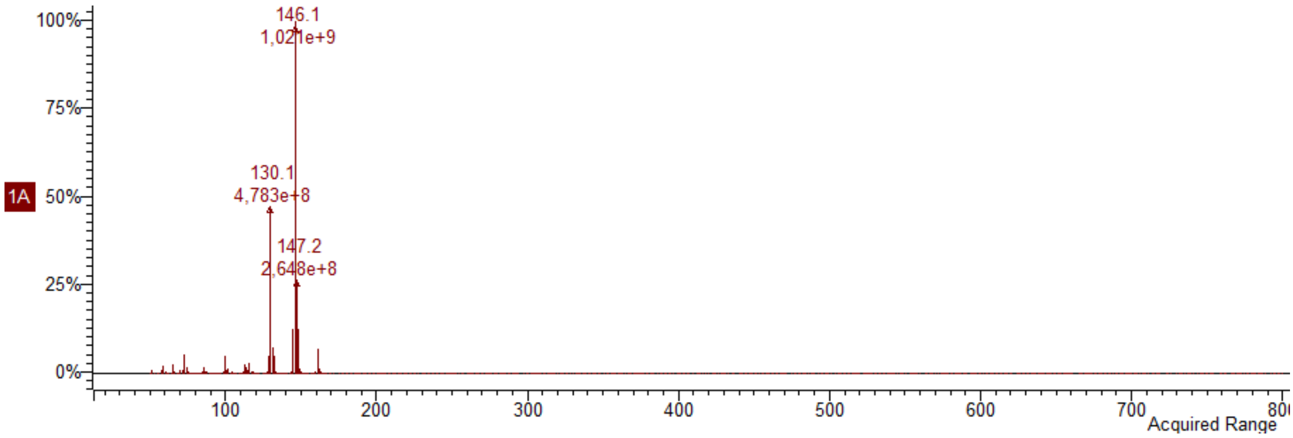

9-Acridinamine (**44**)

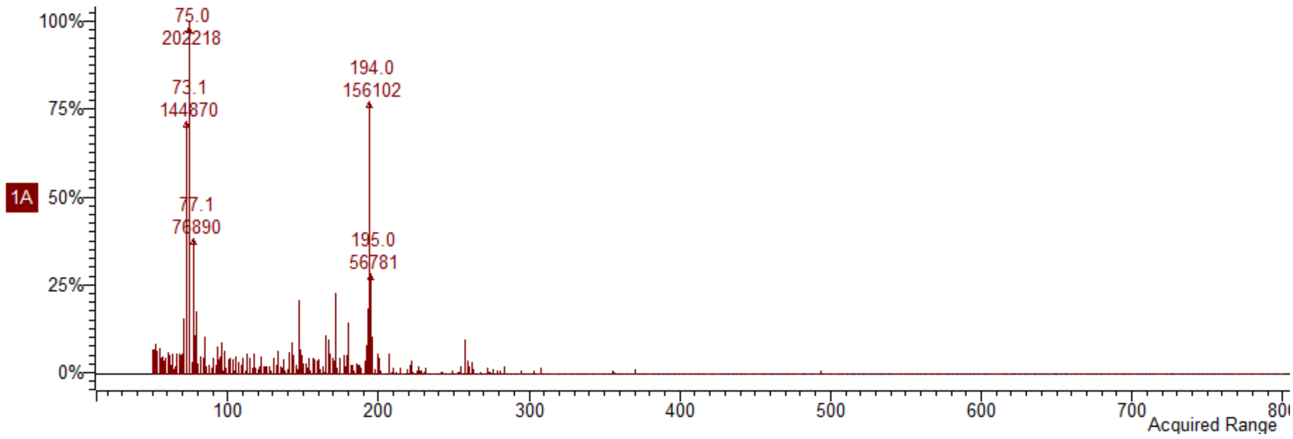

Hydroxy-naphthalene[(a)] (**45**)

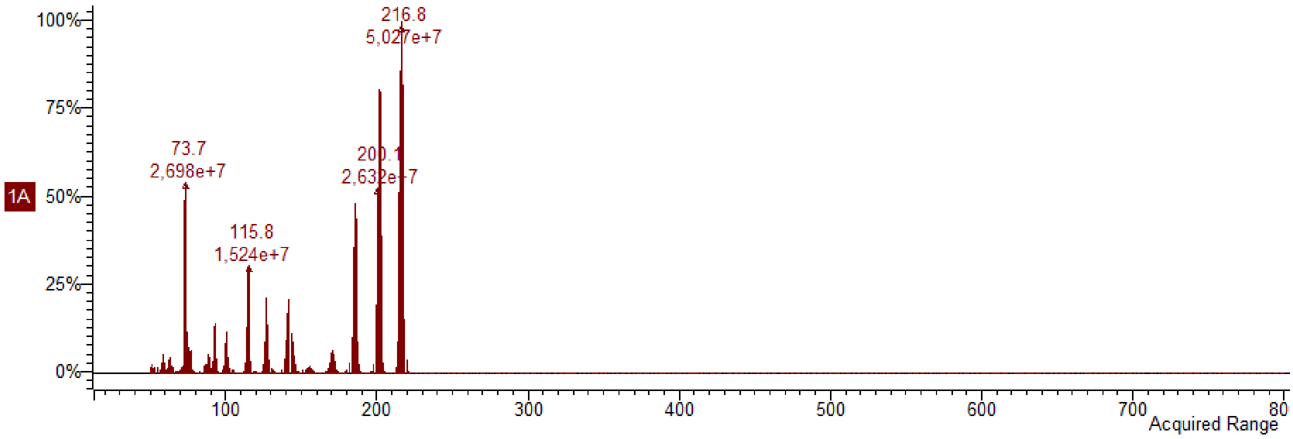

1,8-Dihydroxy-naphthalene[(b)] (**46**)

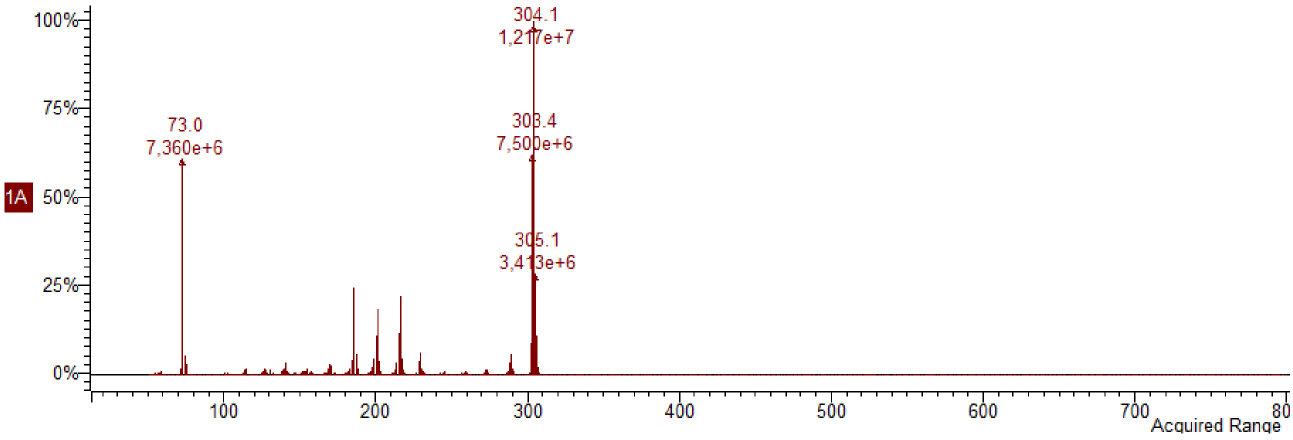

Methyl-naphtalene (**47**)

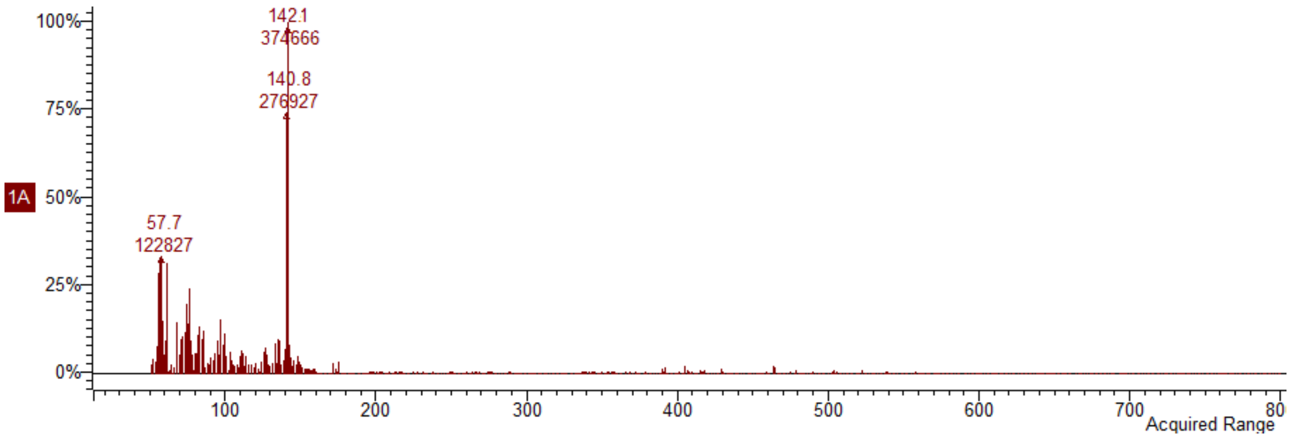

Acenaphthylene (**48**)

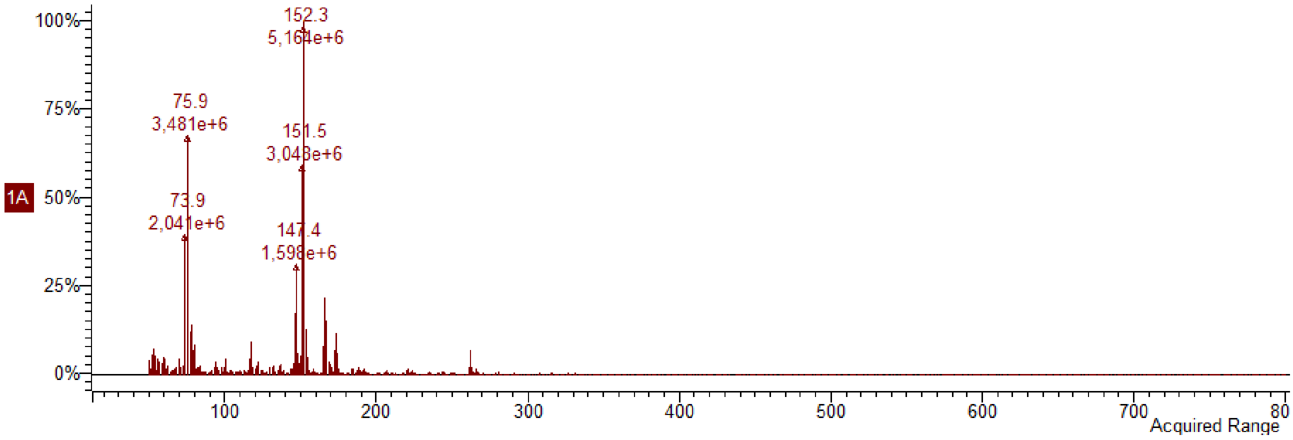

Selected spectra of standards

Formamide[b]

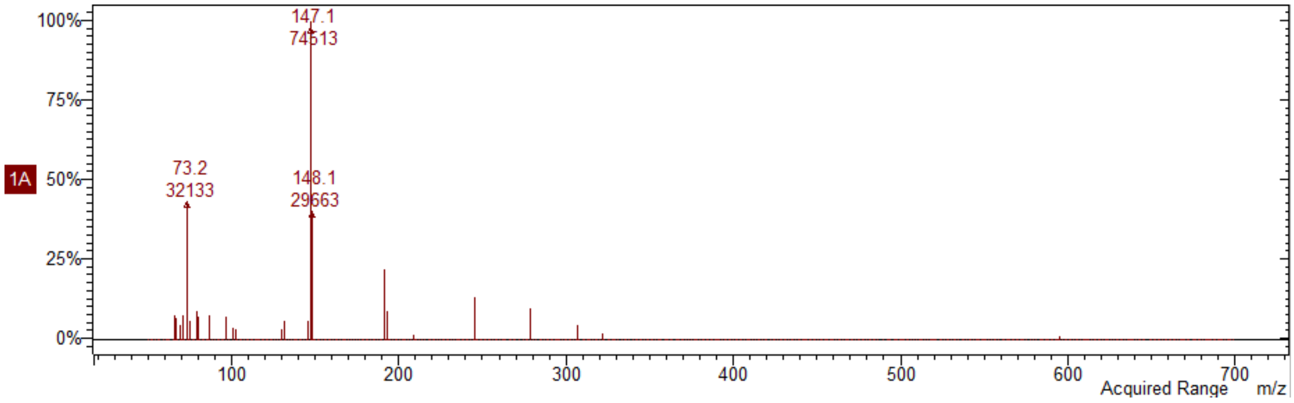

Glycine[b]

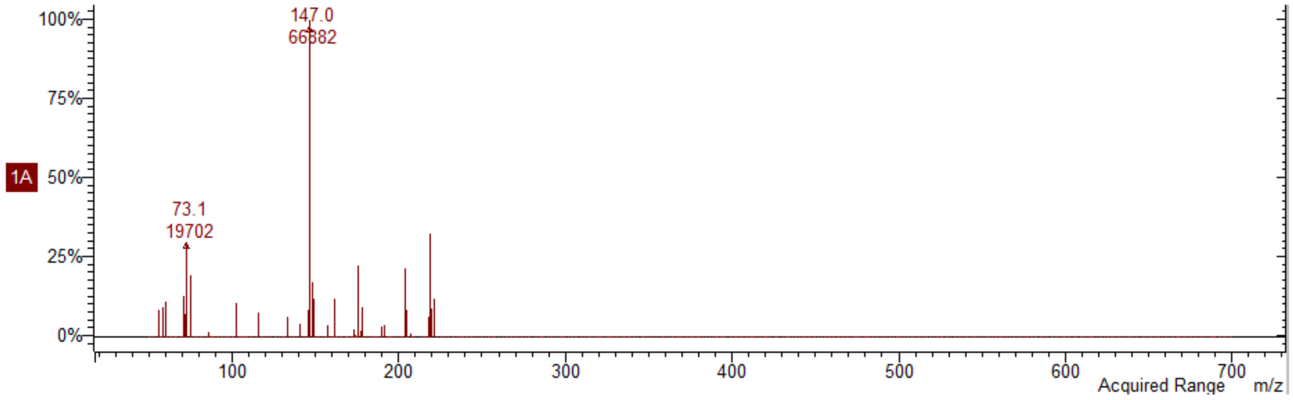

Pyruvic acid[b]

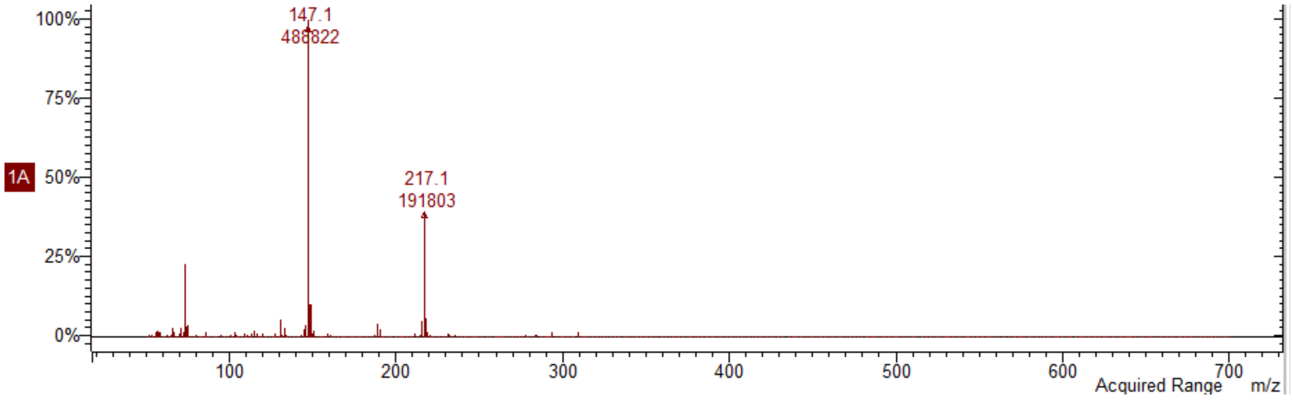

Adenine[a]

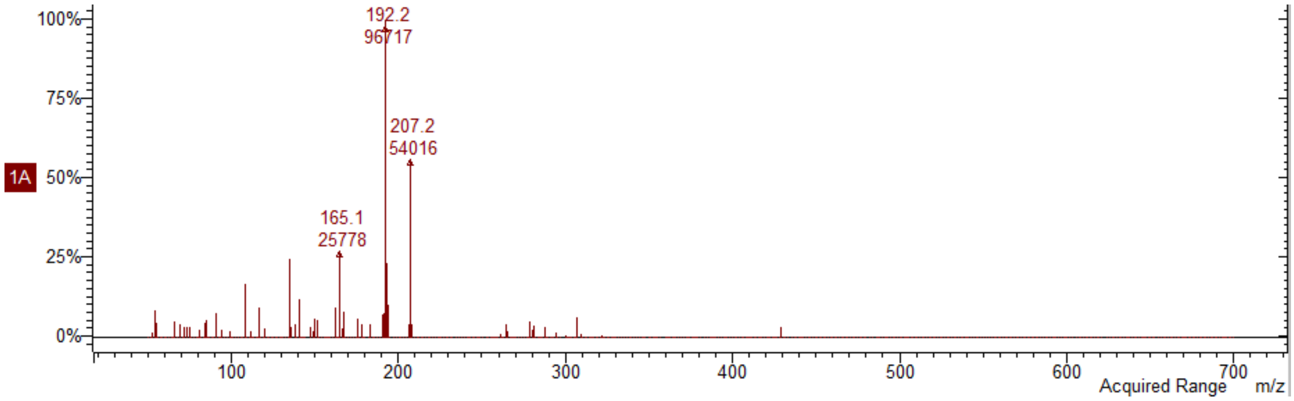

Parabanic acid [b]

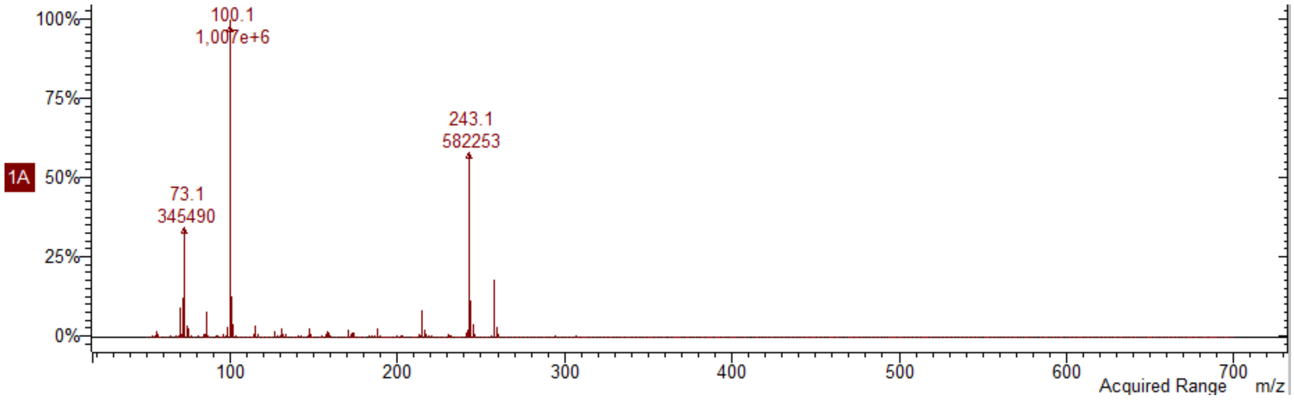

SI #5. Supplementary text.

Glycine and alanine were generally obtained as the most abundant amino acids (Table S2 and Table S3, entries 5-6 and 20 versus entries 7-15). We observed a different effect of NH_4Cl in the selectivity of the reaction depending from the composition of the flask. Hydrophobic amino acids **5-9** and **16-21** were obtained in highest yield in borosilicate under buffered conditions (Table S2), while polar amino acids **10-15** become the major reaction products in absence of NH_4Cl (Table S3). The effect of NH_4Cl in the synthesis of amino acids in the electric discharge of a reducing atmosphere containing CH_4, CO, and CO_2 was previously reported [29]. Several mechanisms could account for the formation of amino acids. These include the Strecker and Bucherer–Bergs condensations[30]. As an alternative, the formation of amino acids from formamide by barrierless condensation with formate ion cannot

be completely ruled out, as inferred by theoretical ab initio simulation of the Miller-Urey experiment [20]. Under the experimental conditions tested here, the higher the amount of formamide and of DAMN, the higher the yield of amino acids (Tables S2-S3), suggesting the possibility of a synergy between HCN and formamide chemistry. Carboxylic acids **27** and **29-32** are the key intermediates of the tricarboxylic acids (rTCA) cycle, which in its reductive version (counterclockwise turn) represents the possible prebiotic ‘‘core’’ of the primitive pre-metabolism[31]. Even if the formation of complex carboxylic acids is expected to occur in the Miller-Urey experiment, a detailed analysis of their structural variety is not available. The simplest carboxylic acids (from C-1 to C-3) prevailed with respect to the higher molecular weight counterpart (Tables S2-S3, entries 25-27 versus entries 28-35). As a general trend, the total yield of carboxylic acids increased in the absence of the buffer, in which case a major molecular complexity, represented by larger amount of α-ketoglutaric acid, hexanoic acid, gentisic acid and nonanoic acid, was observed. The involvement of DAMN in the prebiotic synthesis of carboxylic acids was reported in a large variety of experimental conditions. For example, oxaloacetic acid is produced from DAMN by a cascade of hydrolysis and redox processes. The successive decarboxylation and disproportionation of oxaloacetic acid yields pyruvic acid and malic acid, from which fumaric acid is obtained by simple dehydration and succinic acid by reductive dihydroxylation[32]. Carboxylic acid derivatives are also synthesized from formamide in a large panel of energy and environmental conditions, and Fischer-Tropsch like-processes are most probably responsible for the formation of the high molecular weight monocarboxylic acid derivatives [13,14]. Few examples are available for the synthesis of nucleic acid bases in electric-discharge reactions. Trace amounts of adenine, guanine and isocytosine have been detected after electric-discharge of NH_3, CH_4, ethane and H_2O[33]. More recently, RNA and DNA nucleobases were obtained in the electric-discharge of the reducing NH_3, CO, and H_2O atmosphere in unbuffered conditions[21]. In this latter case, theoretical calculations confirmed the key role played by formamide and DAMN in the formation of purine and pyrimidine nucleobases, as a consequence of energy favored processes including 4-amino-5-cyanoimidazole (AlCN) and 4-amino-5-carboxyamideimidazole (AICA) as intermediates. This reaction pathway is in agreement with the higher amount of formamide and DAMN detected in the borosilicate systems in the absence of the buffer, which are the optimal experimental conditions for the formation of nucleobases. Among the miscellanea of aromatic derivatives, compounds **45** and **46**, never previously detected in the Miller-Urey discharge-like experiments, behave as chemical precursors of oxygenated derivatives of PAH (oxy-PAH). These compounds are more plausible prebiotic candidates than simple PAHs because of their higher reactivity and polarity, ensuring stronger molecular recognition processes and higher facility to give degradation and polymerization reactions[34]. In addition, oxy-PAHs may be associated to the Insoluble Organic Matter (IOM), the

major identified carbonaceous component in meteorites of the chondrite type. The selectivity in the synthesis of aromatic derivatives was strictly dependent from the experimental conditions. They largely prevailed in the borosilicate systems in the presence of the buffer, while these compounds were obtained in comparable yield under unbuffered conditions in both borosilicate and Teflon flasks (Tables S2-S3). In addition, 1,8-dihydroxynaphtalene and methylnaphthalene were produced only in the presence of the buffer. The hydrogen-abstraction/acetylene addition (HACA) mechanism, described for the formation of naphthalene in high energy radical conditions, may take account for the formation of PAHs and oxy-PAHs during the electric discharge[35].

SI #6. Analysis of insoluble material

GC-MS. The sample was analyzed as follows: a) analysis after derivatization with BSTFA and TMCS (Condition A); and b) analysis after acidic hydrolysis (28) followed by derivatization with BSTFA and TMCS (Condition B). Briefly, the sample (5.0 mg) was suspended in HCl 6.0 N (1.0 mL) and heated at 110°C for 24 h. Thereafter, the soluble fraction was removed by centrifugation and freeze dried, while the dark insoluble residue was not further analyzed. The derivatization on the soluble fraction was performed as described in SI#1. The GC-MS chromatographic profiles of samples A and B are reported in Figure S5 and S6, respectively, while Table S5 and Scheme A describe the yield and retention time (min) of the main identified products. Finally, original m/z fragmentation spectra, ion abundance and MS fragmentation profiles of novel compounds are reported in figure S7 and Table S6, respectively. Structural data were in accordance with that of commercially available standard compounds. Note that, with a few exceptions, samples A and B showed a similar GC-MS behavior irrespective from the hydrolytic treatment, suggesting that only a few number of compounds was delivered by the acidic treatment. In accordance with data previously reported, the large cluster of peaks comprised in the retention time range between 24 min and 30 min and characterized by repetitive mass-fragmentation values (m/z 149.4, 94.7 and 58.7) (figure S7), can be associated to the presence of isomeric HCN oligomers (Origins of life 1975, 6, 513-525). In addition, the following compounds have been identified in lower amount: urea **3**, glycine **5**, lactic acid **28**, adenine **36**, cytosine **39**, guanidine **49**, succinic acid **50**, 2,4-diamino-6-hydroxypyrimidine **51**, hypoxanthine **52**, and four polycyclic aromatic hydrocarbons (PAHs), namely anthracene **53,** crysene **54**, pyrene **55**, and dibenz(*a,h*)anthracene **56** (Scheme A).

Most probably these compounds were originally embedded in the solid matrix and successively extracted after the derivatization procedure. Among them, **49-56** were not previously recovered from the liquid fraction of the Miller-like discharge experiments. In addition, crysene **54** and succinic acid **50** were isolated only after the hydrolytic treatment. As a general trend, the yield of isolated compounds was found to be increased after the acid hydrolysis, highlighting the possibility that the treatment favored the extraction of the compounds from the solid matrix (Table S5; sample A vs sample B).

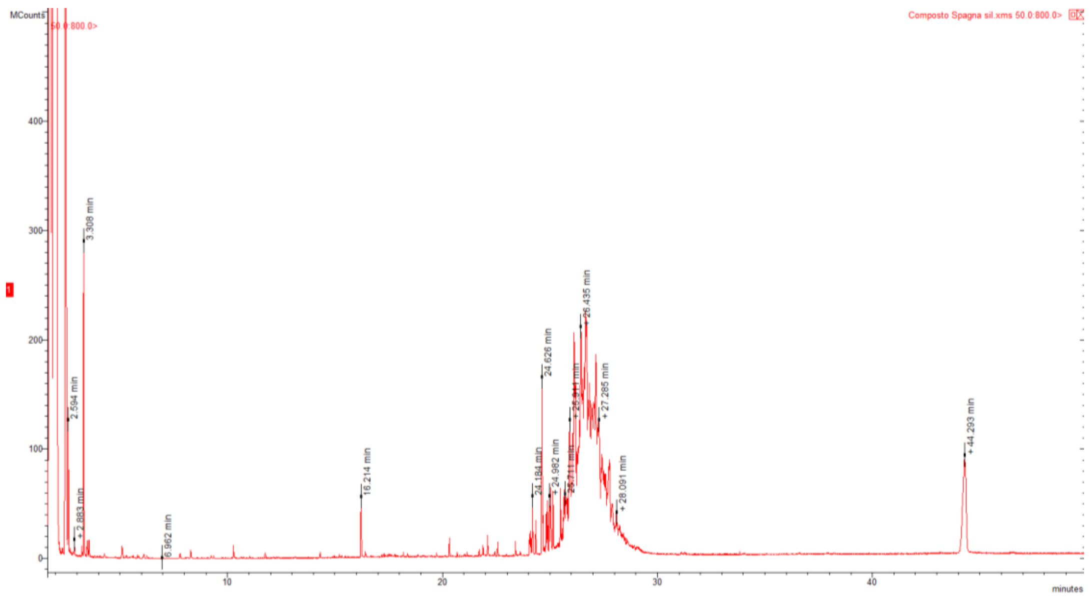

Figure S5. GC-MS chromatogram of sample A

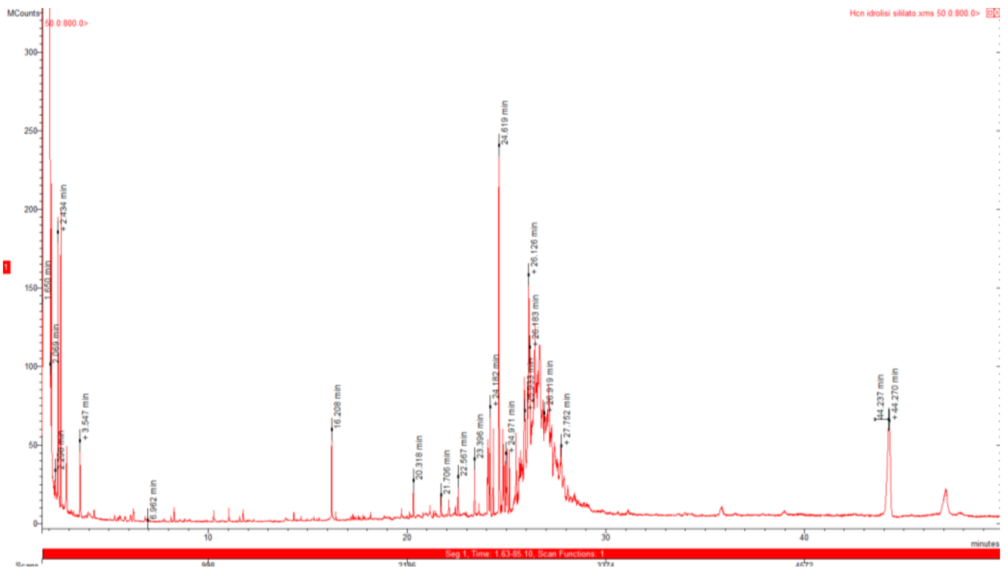

Figure S6. GC-MS chromatogram of sample B

Table S5. Identified compounds after releasing from insoluble matter:

Entry	Compound	Not hydrolyzed insoluble matter Condition A		Hydrolyzed insoluble matter Condition B	
		Rt (min)	Yield	Rt (min)	Yield
1	Urea (**3**)	9.226[b]	1,91(f)	-	-
2	Glycine (**5**)	3.473[c]	13,40(f)	-	-
3	Lactic acid (**28**)	5.799[c]	0,81(f)	5.799[c]	1,04(f)
4	Adenine (**36**)	5.299[a] 14.967[b]	3,53(f)	5.299[a] 14.967[b]	7,15(f)
5	Cytosine (**39**)	4.265[a]	3,39(f)	4.265[a]	7,88(f)
6	Guanidine (**49**)	3.552[b]	12,14(f)	3.552	49,78(e)

7	Succinic acid (**50**)	-	-	11.570	3,87(f)
8	2,4-Diamino-6-hydroxypyrimidine (**51**)	11.761[c]	4,32(f)	11.761[c]	8,00(f)
9	Hypoxanthine (**52**)	4.071[b]	1,69(f)	4.071[b]	2,37(f)
10	Anthracene (**53**)	10.278	8,57(f)	10.278	7,97(f)
11	Chrysene (**54**)	-	-	6.826	4,54(f)
12	Pyrene (**55**)	7.790	4,29(f)	7.790	2,30(f)
13	Dibenz[*a,h*]anthracene (**56**)	24.619	117,15(e)	24.619	225,34(e)

The yield is defined as μg of product per 1.0 mg of the crude. Rt retention time (min). Products have been detected with a different degree of silylation: [a] mono-silyl derivative; [b] di-silyl derivative; [c] tri-silyl derivative. (e) Data are the mean values of three experiments with SD of ± 0.05. (f) Data are the mean values of three experiments with SD of ± 0.01.

Table S6. Ion abundance and MS fragmentation profiles of compounds **49-56**

Product	m/z
Guanidine (**49**)	190 [M+2 TMS-Me] (50), 147 [M+2 TMS-4Me] (100)
Succinic acid (**50**)	247 [M+2 TMS-Me](25)
2,4-Diamino-6-hydroxypyrimidine (**51**)	270 [M+2 TMS] (45), 255 [M+2 TMS-Me] (100)
Hypoxanthine (**52**)	280 [M+2 TMS] (40), 265 [M+2 TMS-Me] (100)
Anthracene (**53**)	179 [M+1] (100)
Chrysene (**54**)	229 [M+1] (48), 74 (100)
Pyrene (**55**)	203 [M+1] (30), 188 [M-Me] (75), 74 (100)
Dibenz[*a,h*]anthracene (**56**)	279 [M+1] (17), 149 (100)

Figure S7. Original m/z fragmentation spectra of compounds **49-56** and HCN oligomers

Guanidine (**49**)

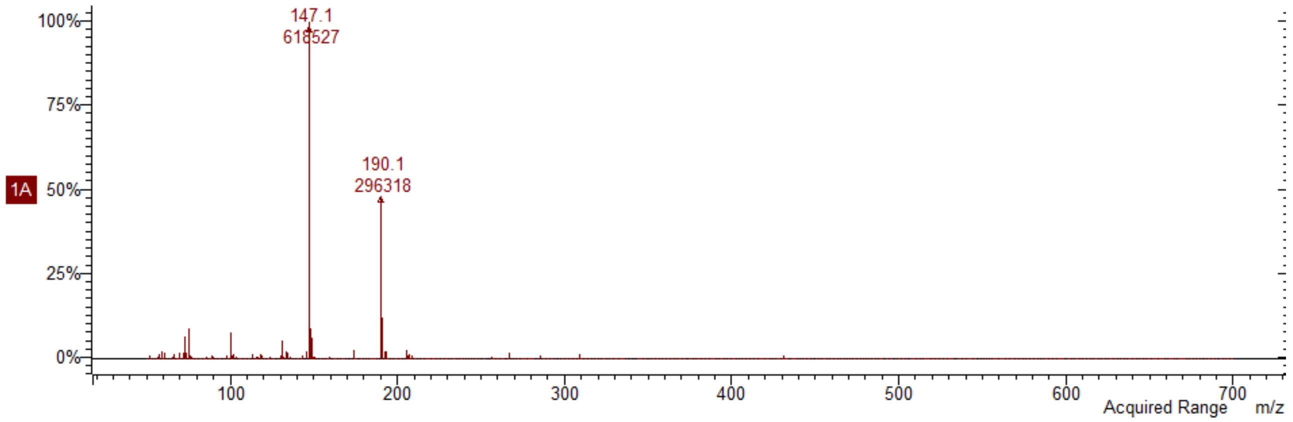

Succinic acid (**50**)

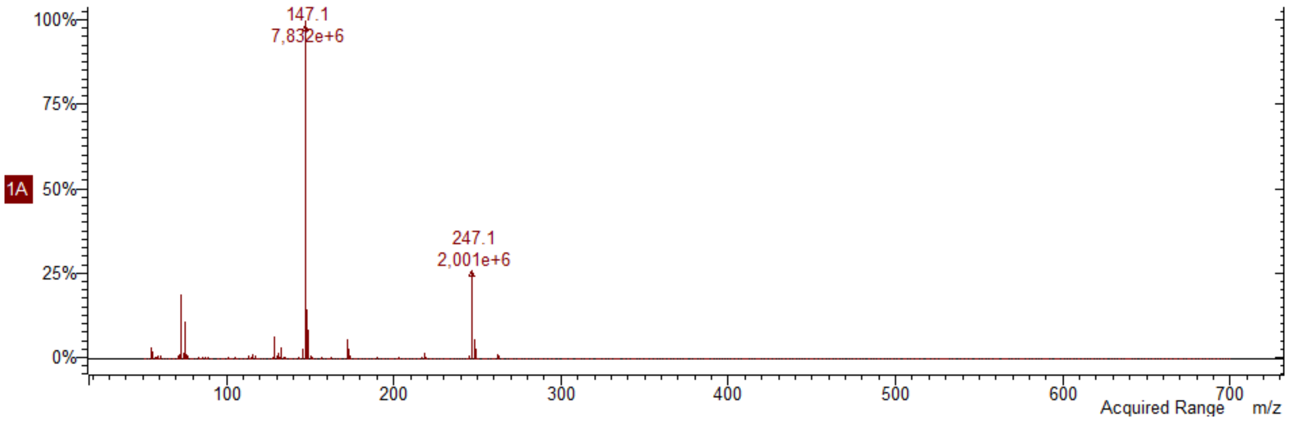

2,4-diamino-6-hydroxypyrimidine (**51**)

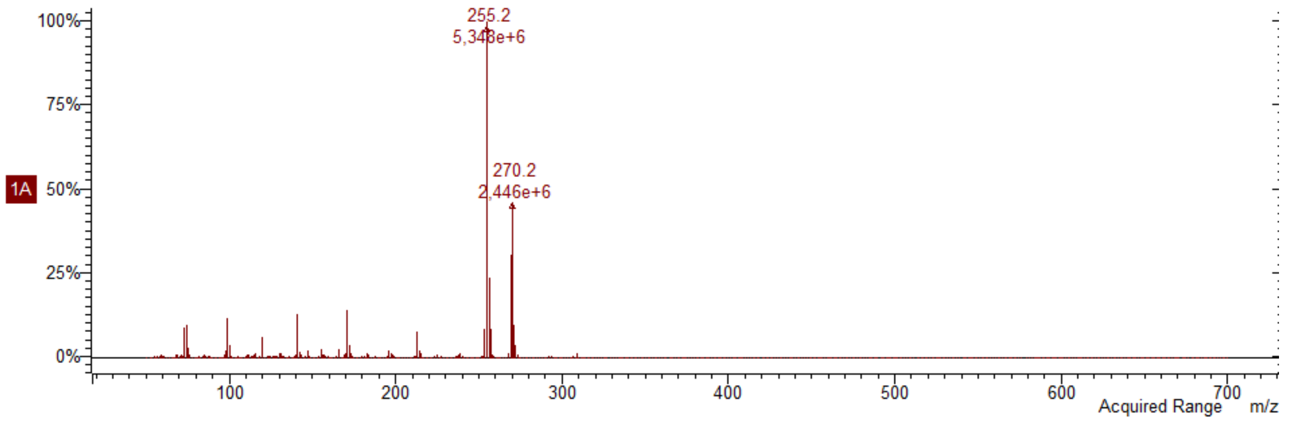

Hypoxanthine (**52**)

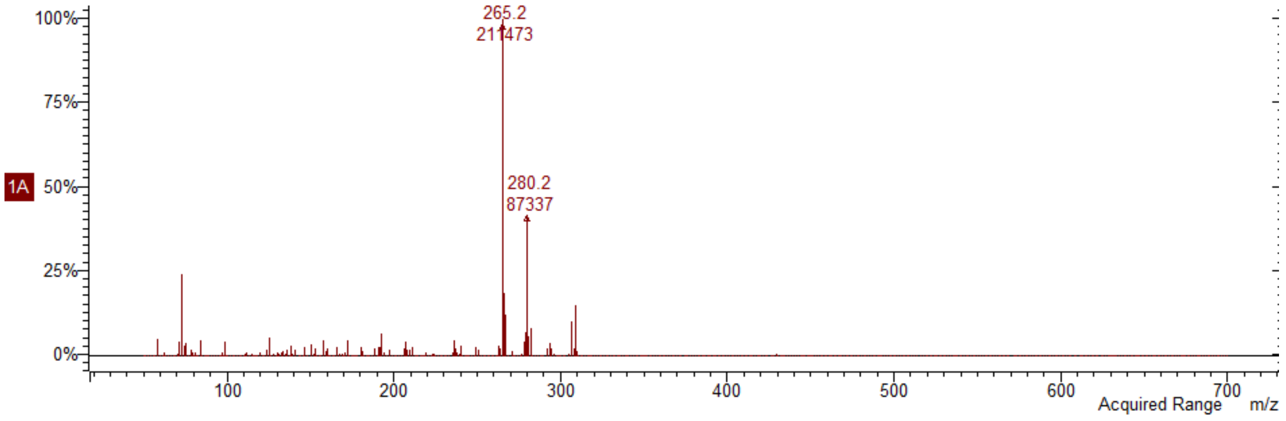

Anthracene (**53**)

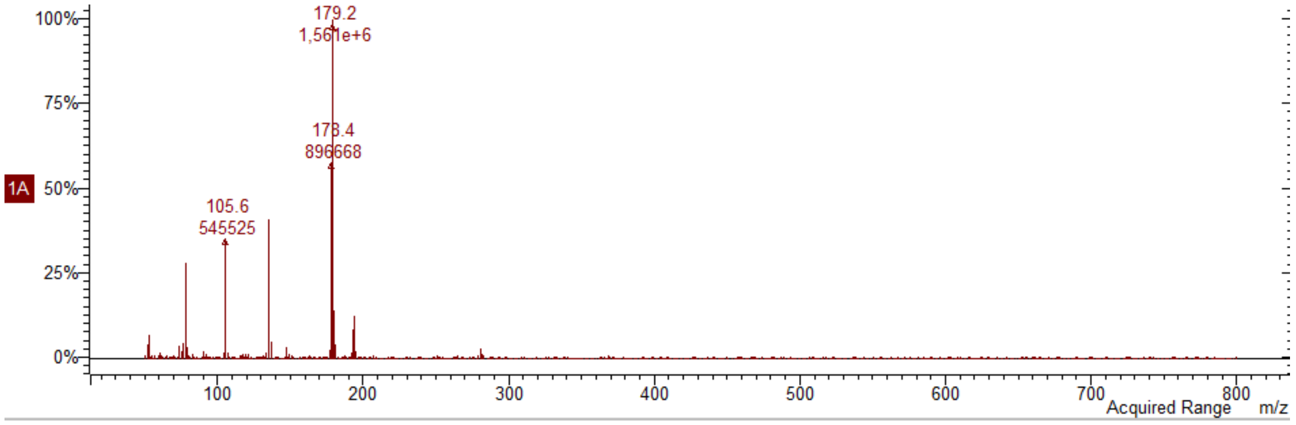

Crysene (**54**)

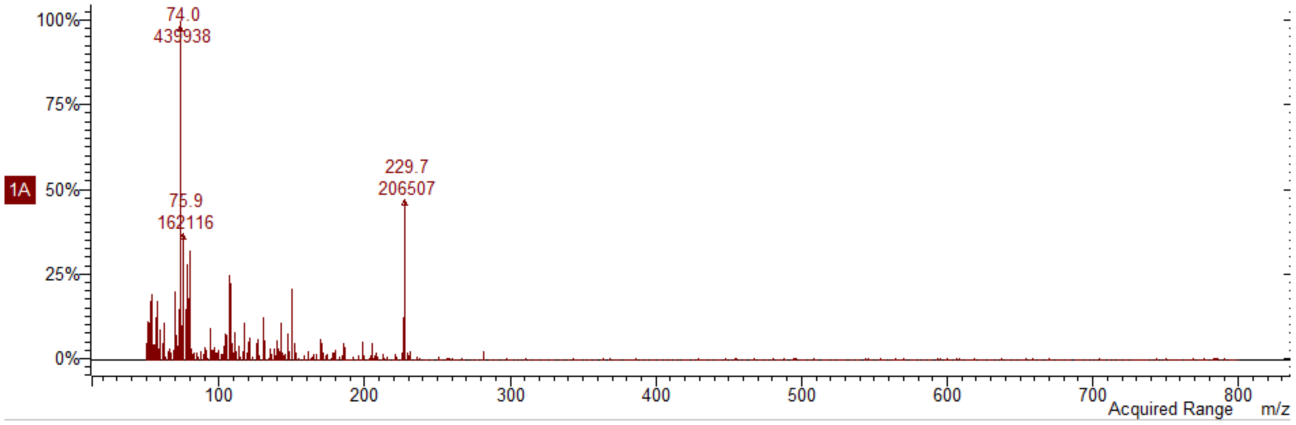

Pyrene (**55**)

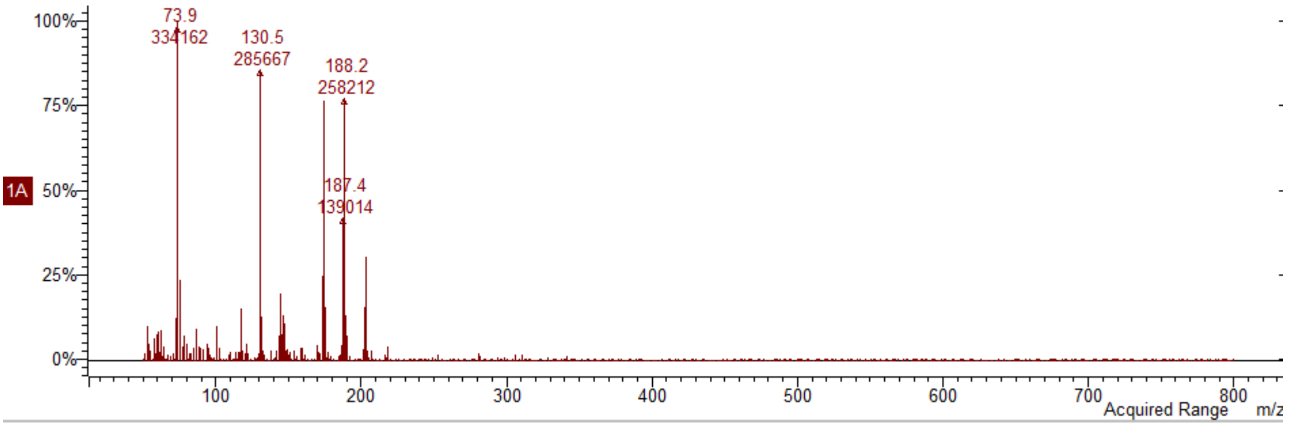

Dibenz(a,h)anthracene (**56**)

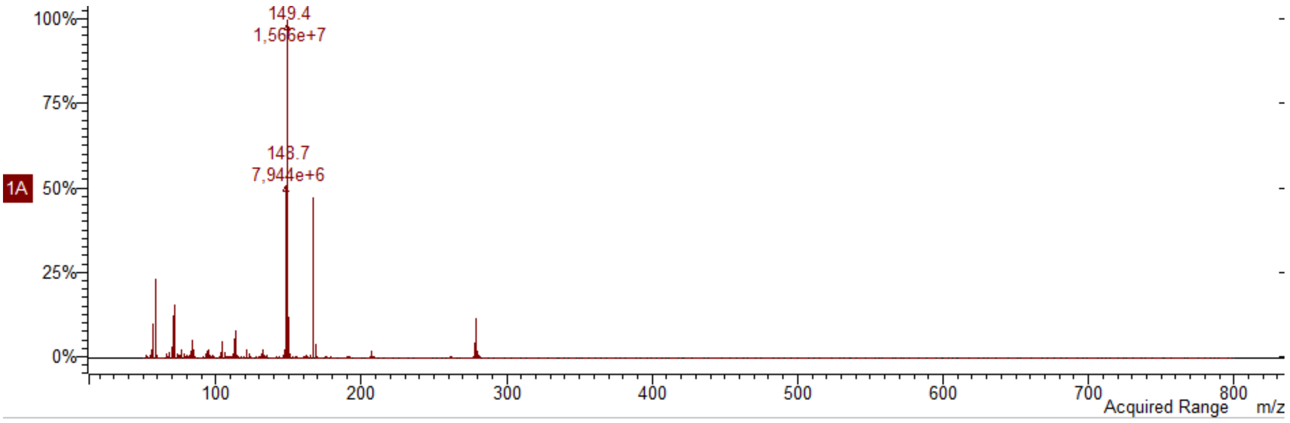

HCN oligomers

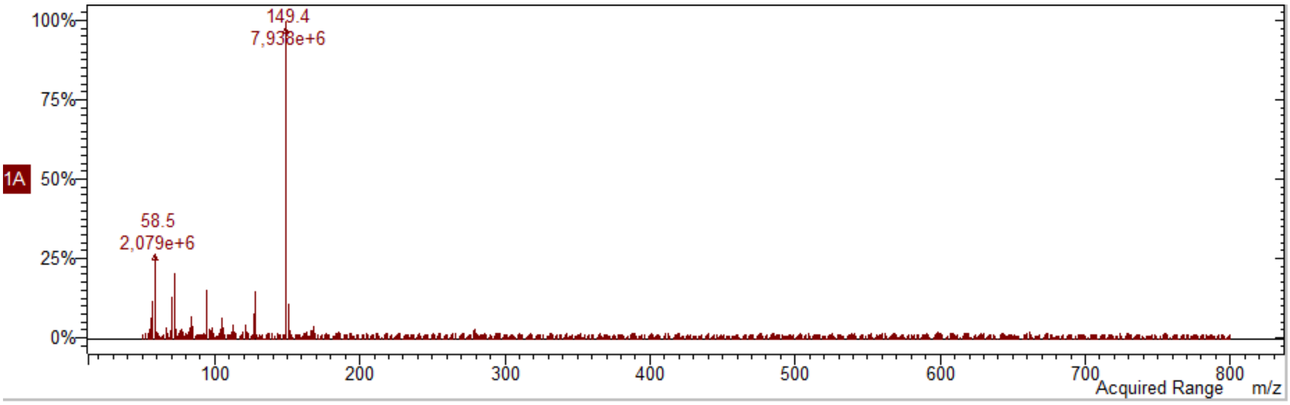

Figure S8. Electron micrograph of a particle included into the insoluble material (top-left). Energy Dispersive X-Ray Scattering of the particle (top-right). Deconvolution of the spectra due to Tungsten (bottom-left). Deconvolution of the spectra due to Silicon (bottom-right).

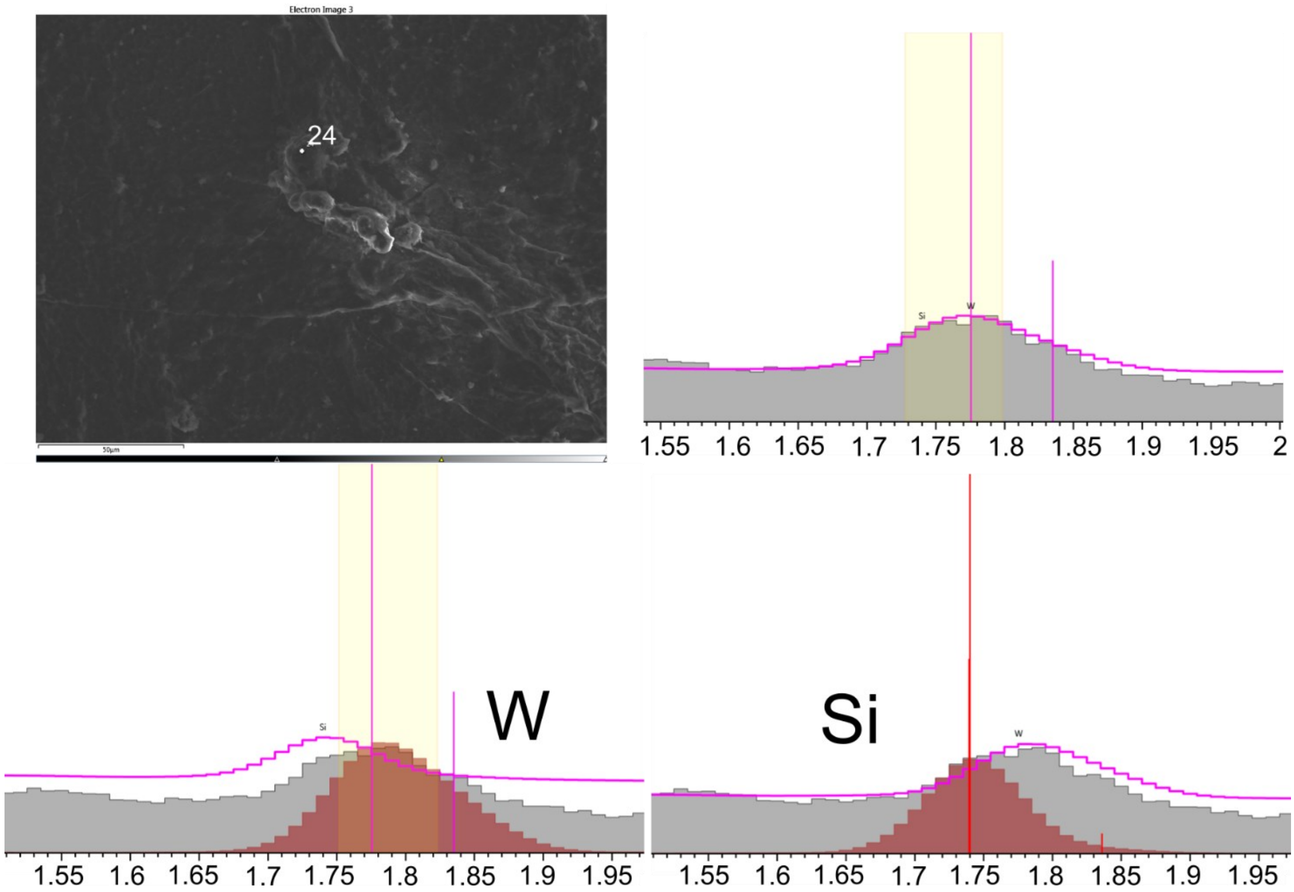

SI #7. Calibration line procedure and internal standard method.

Calibration line procedure

Five solutions of the appropriate standard compound to be determined were prepared at different known concentration and injected in the GC-MS apparatus after the previously reported derivatization procedure. The area of chromatographic peak versus concentration was enregistered by the apparatus, and the calibration line automatically builded by a regression procedure. The calibration line was stored by the apparatus and successively applied in the quantification of the sample (https://www.jove.com/v/10188/calibration-curves).

Internal standard method

The appropriate crude and betulinic acid 3β-hydroxy- 20(29)-lupaene-oic acid (0.2 mg, 0,00045 mmol) were dissolved in a round bottom flask in the presence of *N*,*N*-bis-trimethylsilyl trifluoroacetamide (420 µL; Merck >99%) and pyridine (200 µL; Merck >99%). The mixture was left under magnetic stirring at 90 °C for 4 h. Thereafter the solution was cooled to 25 °C and 2.0 µl

of the solution were used for the GC-MS analysis. The ratio of the peak areas of the selected compound versus internal standard was used by the software MS Workstation to obtain the quantitative data.
